# Cyclophosphamide enhances the antitumor potency of GITR engagement by increasing oligoclonal cytotoxic T cell fitness

**DOI:** 10.1172/jci.insight.151035

**Published:** 2021-10-22

**Authors:** Daniel Hirschhorn, Allison Betof Warner, Rachana Maniyar, Andrew Chow, Levi M.B. Mangarin, Adam D. Cohen, Linda Hamadene, Gabrielle A. Rizzuto, Sadna Budhu, Nathan Suek, Cailian Liu, Alan N. Houghton, Taha Merghoub, Jedd D. Wolchok

**Affiliations:** 1 Swim Across America and Ludwig Collaborative Laboratory, Immunology Program, Parker Institute for Cancer Immunotherapy, and; 2 Human Oncology and Pathogenesis Program, Memorial Sloan Kettering Cancer Center (MSKCC), New York, New York, USA.; 3 Weill Cornell Medical College, New York, New York, USA.; 4 Department of Pathology, University of California, San Francisco, San Francisco, California, USA.

**Keywords:** Immunology, Oncology, Cancer immunotherapy, Melanoma, T cells

## Abstract

Only a subset of cancer patients responds to checkpoint blockade inhibition in the clinic. Strategies to overcome resistance are promising areas of investigation. Targeting glucocorticoid-induced tumor necrosis factor receptor–related protein (GITR) has shown efficacy in preclinical models, but GITR engagement is ineffective in controlling advanced, poorly immunogenic tumors, such as B16 melanoma, and has not yielded benefit in clinical trials. The alkylating agent cyclophosphamide (CTX) depletes regulatory T cells (Tregs), expands tumor-specific effector T cells (Teffs) via homeostatic proliferation, and induces immunogenic cell death. GITR agonism has an inhibitory effect on Tregs and activates Teffs. We therefore hypothesized that CTX and GITR agonism would promote effective antitumor immunity. Here we show that the combination of CTX and GITR agonism controlled tumor growth in clinically relevant mouse models. Mechanistically, we show that the combination therapy caused tumor cell death, clonal expansion of highly active CD8^+^ T cells, and depletion of Tregs by activation-induced cell death. Control of tumor growth was associated with the presence of an expanded population of highly activated, tumor-infiltrating, oligoclonal CD8^+^ T cells that led to a diminished TCR repertoire. Our studies show that the combination of CTX and GITR agonism is a rational chemoimmunotherapeutic approach that warrants further clinical investigation.

## Graphical abstract

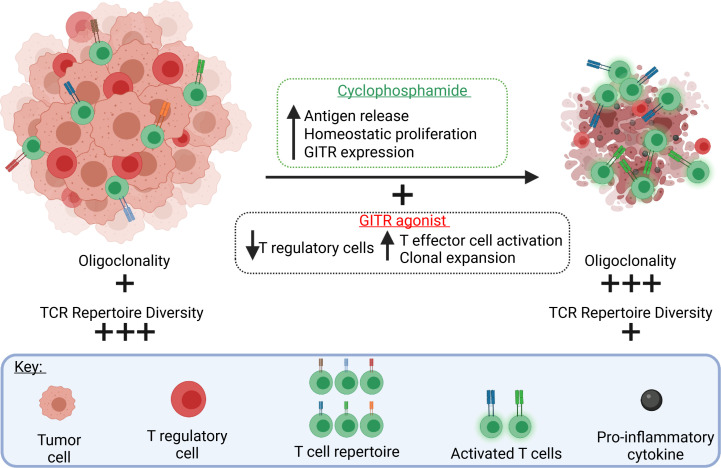

## Introduction

Immune checkpoint blockade is increasingly used to treat a variety of malignancies, but primary and secondary resistance to the FDA-approved cytotoxic T lymphocyte–associated protein 4 (CTLA-4) and programmed cell death protein/ligand 1 (PD-1/PD-L1) inhibitors limit the clinical benefit of these agents (1, 2). One mechanism of both primary and secondary resistance is the presence of CD4^+^Foxp3^+^ regulatory T cells (Tregs) in the tumor microenvironment (3). In healthy individuals, Tregs promote self-tolerance and maintain immune homeostasis during stimulation by foreign antigens. Tumors co-opt this mechanism and recruit Tregs that suppress antitumor immune responses in mice (4). Extensive Treg infiltration is also associated with poor prognosis in solid human tumors (5). It is thus reasonable to deduce that therapeutic depletion of Tregs while simultaneously maintaining or enhancing the presence of CD8^+^ effector T cells (Teffs) via engagement of costimulatory receptors can overcome resistance to checkpoint blockade.

Glucocorticoid-induced tumor necrosis factor receptor–related protein (GITR), a member of the tumor necrosis factor receptor family, is constitutively expressed at high levels by Tregs and is upregulated when Teffs are activated (6). Engagement of GITR on Tregs inhibits the Foxp3-dependent suppressive lineage commitment, thereby limiting Treg functionality (7, 8). GITR engagement provides a costimulatory signal that expands and activates conventional Teffs (9). Thus, GITR agonism increases the ratio of Teff/Treg and promotes antitumor immunity in murine models (6, 7, 10). We have previously demonstrated that a monoclonal agonist GITR antibody promotes tumor rejection in preclinical models (11–14). However, in clinical trials, GITR agonism as a monotherapy has failed to effectively control tumor growth, likely due to insufficient Treg modulation and exhaustion of Teffs (15, 16). To address T cell exhaustion, previous work by our group combined GITR agonist antibodies with anti–PD-1 in the B16 melanoma model, demonstrating effective tumor control (16). ClinicalTrials.gov NCT02628574 was designed to evaluate GITR agonism alone or in combination with anti–PD-1 therapy or gemcitabine, but data are not yet available.

Another active area of investigation is the strategy of combining chemotherapy and immune checkpoint blockade, in part due to the potentially additive or even synergistic effects of these agents (17). Cytotoxic agents can stimulate antitumor responses by killing tumor cells to release potentially antigenic debris, increasing immune infiltration, depleting Tregs and myeloid-derived suppressor cells, and enabling homeostatic proliferation of antigen-specific Teffs (18–22).

Cyclophosphamide (CTX) is an alkylating agent with direct antitumor activity that can increase antigen cross-presentation and cross-priming (23) and also suppress Tregs (24–26). Lymphodepletion induced by CTX is followed by a recovery phase, allowing for expansion of tumor-specific Teffs (22). During the recovery phase, proinflammatory cytokines, including interferons, are upregulated and can promote antitumor activity (27, 28).

Cytotoxic agents such as CTX cause DNA damage and immunogenic cell death of tumor cells (29, 30). The surviving cells can accumulate mutations promoting the development of neoantigens, which can trigger antitumor T cells. One report demonstrates that treating poor responders to immune checkpoint inhibitors with cytotoxic chemotherapy induces subclonal neoantigens (31). Additionally, gliomas that recur after treatment with temozolomide (a DNA-alkylating agent) contain several mutations with neoantigen potential (32, 33). Therefore, CTX could improve an antitumor response by promoting the development of neoantigens.

Effective immune control of tumor growth is likely a result of Treg suppression coupled with expansion, infiltration, and activation of CD8^+^ Teffs, which have the capacity to kill tumor cells upon recognition of specific antigens by the T cell receptor (TCR). High-throughput TCR sequencing allows quantification of T cell diversity and identification of tumor-reactive T cell clonotypes (34). High TCR clonality correlates with oligoclonal expansion and improved immunotherapy efficacy in melanoma and other solid tumors (35–38). T cell clonality both in the periphery and within the tumor might certainly be altered as T cells recover after CTX-induced lymphodepletion.

In this study we hypothesize that administration of CTX prior to agonist anti-GITR antibodies would generate a potent antitumor response because the GITR activating signal would be delivered to Teffs during the recovery phase from lymphodepletion.

We demonstrate that CTX, in combination with anti-GITR, promoted durable antitumor responses in clinically relevant tumor models, such as MPC-11 plasmacytoma and the poorly immunogenic B16 melanoma. This combination induced tumor cell death and potently suppressed Tregs, thereby resulting in highly activated CD8^+^ Teffs and increased oligoclonal cytotoxic T cell fitness.

## Results

### Dose-dependent modulation of GITR expression in T cells by CTX.

**Figure 1:**
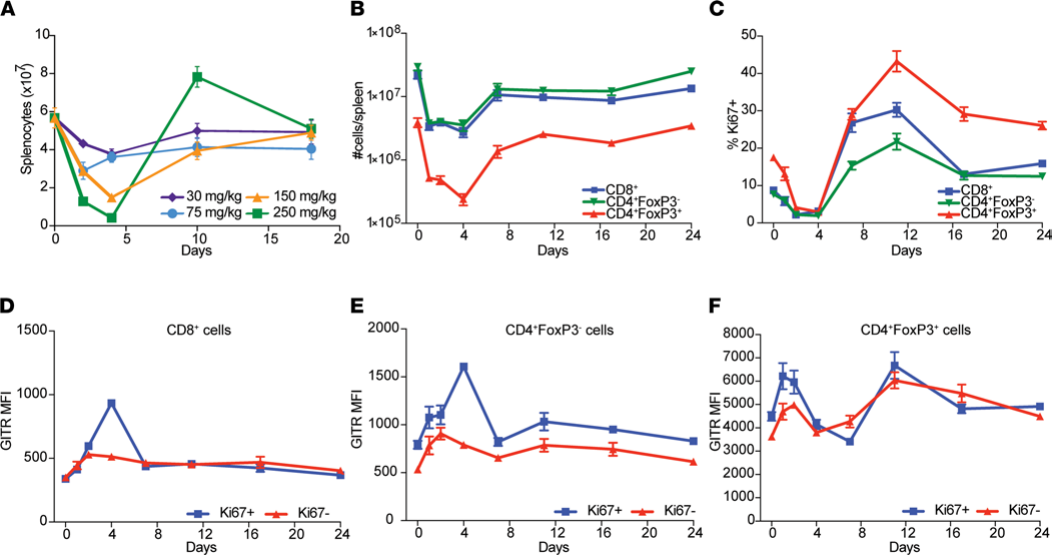
CTX modulates GITR expression in T cell populations in a dose-dependent manner. (**A**) Cohorts of 5 mice were treated with 30, 75, 150, and 250 mg/kg CTX. Single-cell suspensions were prepared from spleens at the depicted days and analyzed for total cellularity by flow cytometry. Number of cells plotted against day post-CTX. (**B**–**F**) Cohorts of 5 mice were treated with 250 mg/kg of CTX. Single-cell suspensions were prepared from the spleens at depicted days and analyzed by flow cytometry. (**B**) Number of splenic CD8^+^, CD4^+^FoxP3^–^, and CD4^+^FoxP3^+^ T cells show nadir at day 4 with a homeostatic recovery at day 11. (**C**) Percentages of Ki67^+^ CD8^+^, CD4^+^FoxP3^–^, and CD4^+^FoxP3^+^ T cells peak at 11 days. (**D**) GITR MFI on Ki67^+^ and Ki67^–^ CD8^+^ gate. (**E**) GITR MFI on Ki67^+^ and Ki67^–^ CD4^+^FoxP3^–^ gate. (**F**) GITR MFI on Ki67^+^ and Ki67^–^ CD4^+^FoxP3^+^ gate. Symbols represent the average of 5 mice at a given day ± SEM. This experiment was repeated at least 3 times with similar results.

CTX causes acute lymphodepletion followed by homeostatic proliferation. We administered increasing doses of CTX ranging from 30–250 mg/kg intraperitoneally to mice and tested overall cellular composition in the spleen at different time points (Figure 1A). Low doses of CTX (30 and 75 mg/kg) induced mild splenocyte depletion, whereas higher doses (150 and 250 mg/kg) promoted robust depletion of splenocytes, with a nadir at day 4 after treatment. Interestingly, at the 250 mg/kg dose, we observed an increased rebound in total splenic cellularity with approximately 50% more cells than pretreatment at day 10. We chose the 250 mg/kg dose for further experimentation because of this most profound change in splenic cellularity. At the 250 mg/kg dose, T cells in the spleen (CD8^+^ CD4^+^Foxp3^–^ conventional T [Tconv] cells and CD4^+^Foxp3^+^ Tregs) showed similar kinetics to total splenocytes with a nadir at day 4 and recovery to baseline numbers at day 7 (Figure 1B). Increased levels of the proliferation marker Ki67 were found in all T cell subsets tested on day 11, about 1 week after the nadir, indicating extensive homeostatic proliferation (Figure 1C). Given that homeostatic proliferation promotes T cell activation, we next tested whether CTX administration modulates the expression of activation markers, such as GITR. A modest modulation of GITR expression on CD4^+^ Foxp3^–^ Tconv and CD8^+^ T cells was observed, with greater effects on CD4^+^ Foxp3^+^ Tregs (Figure 1, D–F). Closer examination of Tconv cells revealed that GITR was upregulated predominantly by the Ki67^+^ fraction of CD8^+^ and CD4^+^ Tconv cells, consistent with an activated phenotype in the homeostatically proliferating cells (Figure 1, D and E). Both the Ki67^+^ and Ki67^–^ fractions of Tregs showed similar fluctuations in GITR expression after CTX treatment (Figure 1F). In summary, a CTX dose of 250 mg/kg caused lymphopenia that was followed by homeostatic proliferation and changes in the level of GITR expression on Tconv cells and Tregs.

### The combination of CTX and anti-GITR effectively controls B16 melanoma progression.

**Figure 2:**
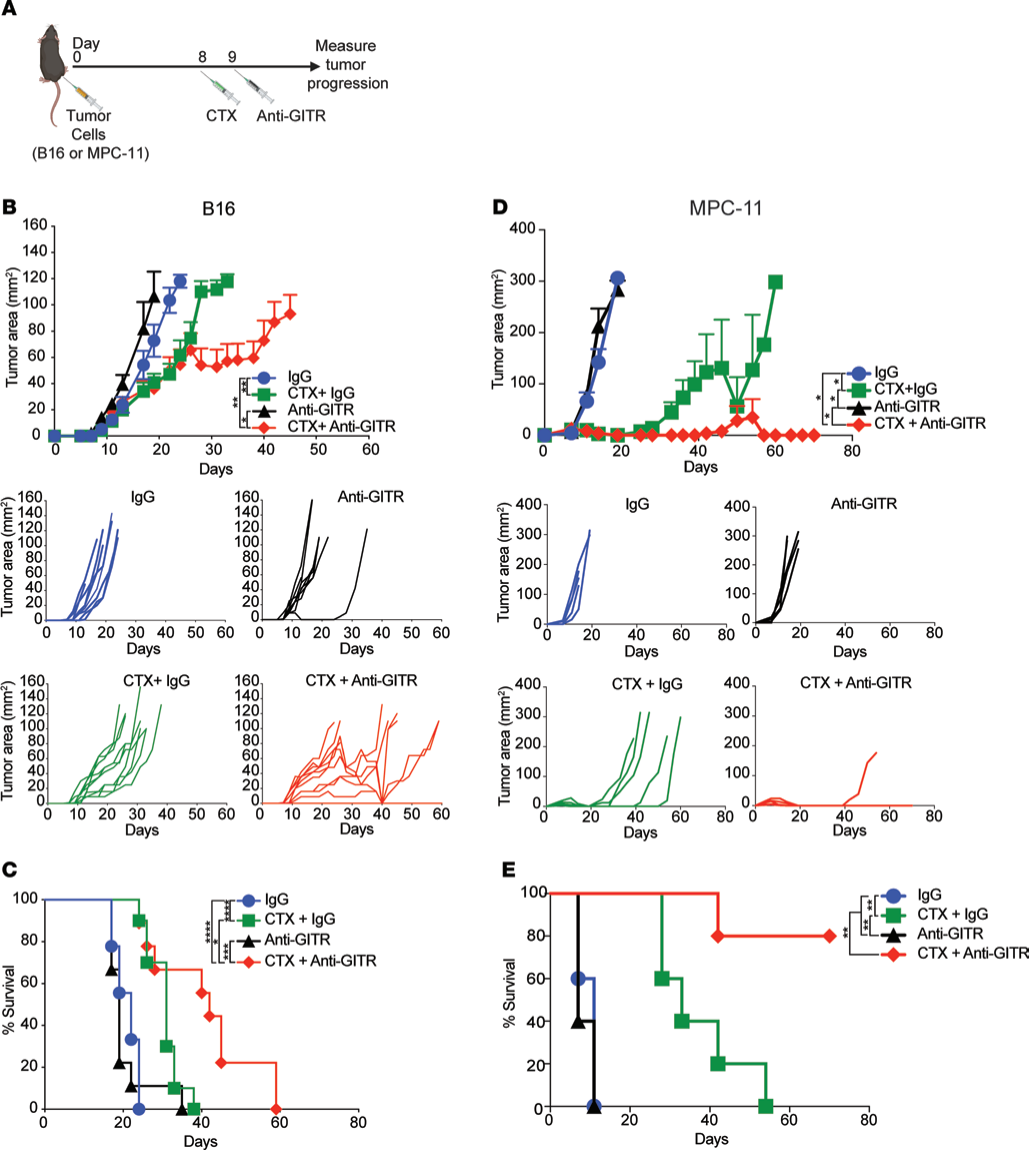
CTX synergizes with anti-GITR to promote potent tumor immunity. (**A**) Experiment schema: Cohorts of 10 mice were implanted intradermally in the flank with tumor cells (**B** and **C**) B16 melanoma and (**D** and **E**) MPC-11 plasmacytoma. On day 8, once tumors were palpable, CTX or PBS was injected. On day 9, mice were treated with anti-GITR antibody or rat IgG. Tumor size was measured 3 times weekly. (**B**) B16 melanoma tumor growth curves (top); growth curves of individual mice over time by treatment group (bottom). Only groups treated with combination therapy demonstrated regression. (**C**) Kaplan-Meier survival plot demonstrating improved overall survival in the B16-bearing mice treated with CTX + anti-GITR. (**D**) MPC-11 plasmacytoma tumor growth curves (top); growth curves of individual mice over time by treatment group (bottom). (**E**) Kaplan-Meier survival plot demonstrating improved overall survival in the MPC-11–bearing mice treated with CTX + anti-GITR. Symbols represent average tumor area ± SEM. Two-way ANOVA was used followed by a Tukey’s multiple-comparison test for tumor growth curve comparisons. Log-rank (Mantel-Cox) test was used for Kaplan-Meier survival curve comparisons. *P < 0.05, **P < 0.01, ***P < 0.001, ****P < 0.0001. Experiments were repeated twice with similar responses.

Given that CTX modulates GITR expression on T cell subsets, promotes homeostatic proliferation of T cells, and depletes Tregs, we hypothesized that CTX administration could enhance the antitumor properties of GITR agonist antibodies. We treated established B16 melanoma tumors with 250 mg/kg CTX on day 8 followed by the GITR agonist antibody DTA-1 on day 9 (Figure 2A). Tumor growth of B16 melanoma was significantly delayed with CTX + anti-GITR agonist combination therapy, and several mice experienced tumor regression (Figure 2B). The combination therapy significantly prolonged survival in this model (Figure 2C). To further demonstrate the versatility of the combination therapy, we treated mice bearing MPC-11 plasmacytoma and observed similar effects (Figure 2, D and E). While CTX showed partial antitumor effects in the models, only the combination therapy with the GITR agonist antibody led to significant tumor regression.

### Superior synergy of GITR agonism and CTX compared with other cytotoxic agents.

**Figure 3:**
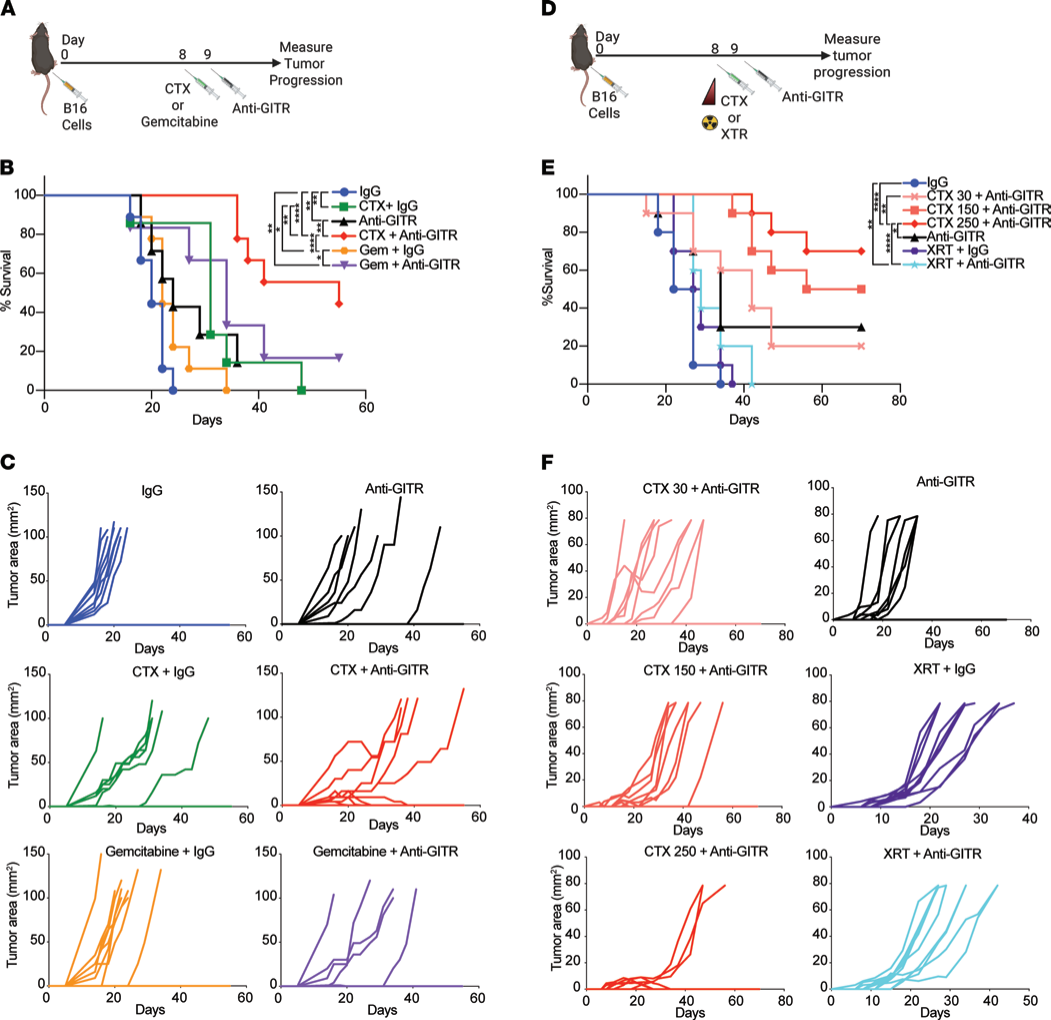
Superior synergy of GITR agonism and CTX compared with other cytotoxic agents. (**A**) Experiment schema for **B** and **C**. Cohorts of 10 mice were implanted intradermally in the flank with B16 melanoma. On day 8, CTX or gemcitabine (Gem) was injected i.p. On day 9, mice were injected with DTA-1 (anti-GITR) or rat IgG. (**B**) Kaplan-Meier survival curves showing improved survival in the CTX + anti-GITR combination treatment groups. (**C**) Tumor growth curves of individual mice per treatment group. (**D**) Experiment schema for **E** and **F**. Cohorts of 10 mice were implanted intradermally with B16 melanoma. On day 8, 30, 150, or 250 mg/kg CTX was injected i.p., or mice received 6 Gy of total-body irradiation. On day 9, mice were treated with DTA-1 (anti-GITR) or rat IgG. (**E**) Kaplan-Meier survival curves showing improved survival only in mice treated with a combination of CTX and anti-GITR. (**F**) Tumor growth curves of individual mice per treatment. Log-rank (Mantel-Cox) test was used for Kaplan-Meier survival curve comparisons. *P < 0.05, **P < 0.01, ****P < 0.0001. Experiments were repeated at least twice with similar results.

Once we established that the antitumor activity of GITR engagement was enhanced after a single dose of 250 mg/kg CTX, we evaluated whether other cytotoxic agents have similar effects (Figure 3A). Gemcitabine (gem) was selected because of its immunomodulatory properties, particularly on myeloid-derived suppressor cells (21). Similar to CTX, gem causes an initial delay in tumor growth, but unlike CTX, administration of gem did not increase the efficacy of GITR agonism (Figure 3, B and C). Whole-body irradiation induces lymphopenia (39) but does not directly affect the growth of radioresistant B16 (40). We then investigated the effects of total-body irradiation, or lower dose CTX, in combination with anti-GITR (Figure 3D). Tumor rejection was only observed with the combination treatment of anti-GITR and high-dose CTX (Figure 3, E and F). Therefore, alkylating agents are conducive to enhancing the antitumor properties of anti-GITR antibodies over other cytolytic agents tested.

### High-dose CTX causes tumor cell death and induces in situ vaccination.

**Figure 4:**
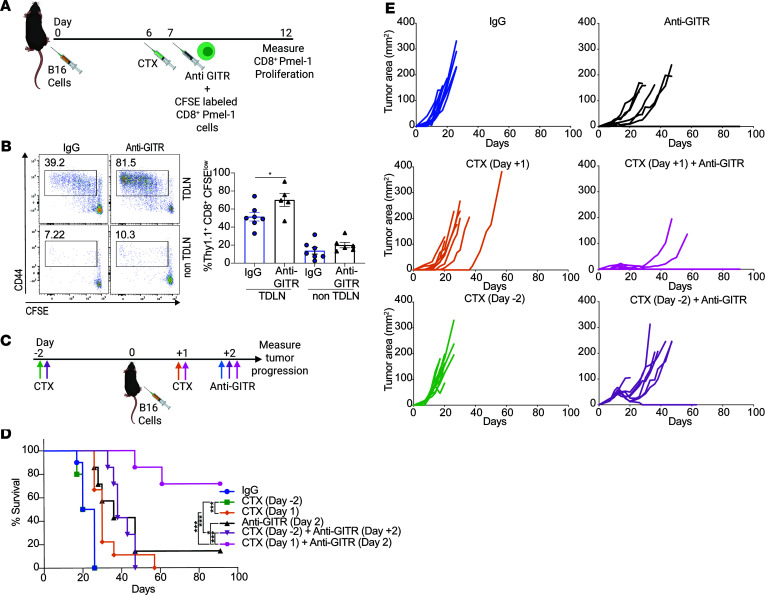
High-dose CTX causes tumor cell death and induces in situ vaccination. (**A**) Experiment schema: Thy1.2 mice were injected with B16. On day 6, groups mice (*n* ≥ 5) were treated with CTX or PBS. On day 7, all mice received CFSE-labeled CD8^+^pmel-1 T cells (Thy1.1) and anti-GITR or rat IgG. On day 12, mice were sacrificed, a single-cell suspension was prepared from inguinal tumor-draining lymph nodes (TDLNs) or contralateral lymph nodes (non-TDLNs), and proliferation was analyzed by flow cytometry. (**B**) Representative flow plot (left) of proliferating antigen-experienced cells gated on DAPI^−^, CD8^+^, and Thy1.1^+^CFSE^lo^. Bars (right) represent the average of 5–7 mice ± SEM. Symbols represent individual mice and lines represent averages ± SEM. Unpaired 2-tailed Student’s *t* test was used to compare IgG and anti-GITR groups. *P < 0.05. (**C**) Experiment schema: Cohorts of mice (*n* = 10) were injected with CTX at day –2 or +1 relative to B16 tumor implant. Some cohorts were injected with DTA-1 at day 2 after tumor challenge. (**D**) Kaplan-Meier survival curves demonstrating improved survival in the group treated with a combination therapy after tumor implant. Log-rank (Mantel-Cox) test was used to compare Kaplan-Meier survival curves. (**E**) Tumor growth curves of individual mice per treatment group demonstrating a regression only when treated with CTX post tumor implant in combination with anti-GITR. *P < 0.05, ***P < 0.001. Experiments were repeated at least twice with similar results.

Once we established that CTX was a superior combination agent, we hypothesized that CTX-induced tumor cell death was contributing to the efficacy of anti-GITR and high-dose CTX. To test this, we measured the proliferation of CD8^+^ T cells by transferring CFSE-labeled CD8^+^ T cells recognizing the melanoma antigen Pmel (41), after CTX administration (Figure 4A). We observed substantial proliferation of tumor-specific T cells in the lymph nodes draining the tumor (TDLNs) but not in the contralateral non-TDLNs. Moreover, further increased proliferation of Pmel-1 T cells was observed in TDLNs compared with non-TDLNs when anti-GITR was administered (Figure 4B). To demonstrate whether the effect was due to CTX alone or the tumor must be present to prime the immune response, we administered CTX either before or after tumor implantation (Figure 4C). Only CTX administration after tumor challenge synergized with GITR agonism in promoting tumor control and improving overall survival (Figure 4, D and E), indicating that tumor cells were essential for the observed response. Overall, these results demonstrate that high-dose CTX combined with anti-GITR antibodies regresses tumors, and this is associated with antigen-specific CD8^+^ T cell proliferation in the TDLNs. This result warrants further investigation into the effects of additional cytotoxic agents in combination with GITR agonism and whether they phenocopy the effects observed with CTX.

### The combination of CTX and anti-GITR decreases intratumor Tregs and increases the ratio of CD8^+^ T cells to Tregs.

**Figure 5:**
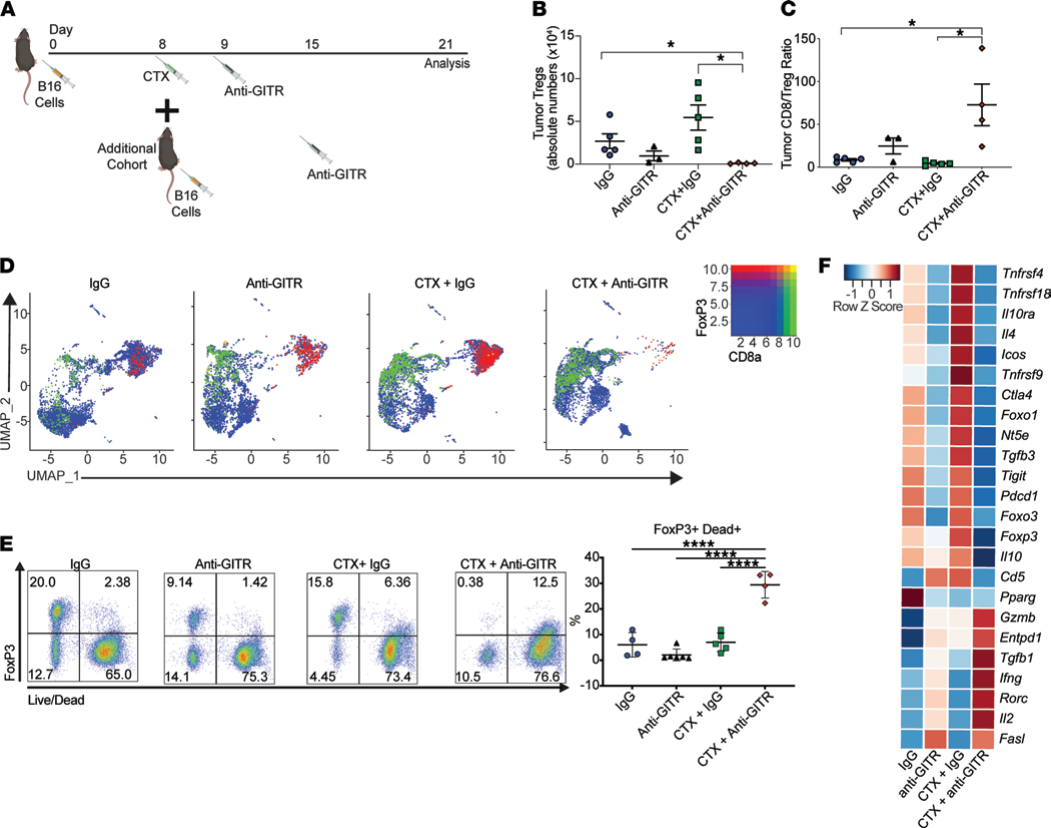
Combination therapy decreases intratumor Tregs and increases CD8/Treg ratio. Mice were implanted with B16 on day 0 and then treated with CTX on day 8 and anti-GITR or control IgG on day 9. Additional cohorts of mice were implanted with B16 on day 8; on day 15 mice were treated with anti-GITR or IgG. After 1 week, all cohorts of mice were sacrificed, and single-cell suspensions were prepared from tumors. Asynchronous B16 challenge was needed for the CTX groups a week apart given the lack of immune infiltrates caused by chemotherapy treatment. (**A**) Experiment schema. (**B**) Absolute number of Tregs per gram of tumor per treatment group demonstrating a reduction in Tregs in the combination treatment group. (**C**) Ratio of total CD8^+^ T cells to Tregs measured by flow cytometry demonstrating an increase in the combination treatment group. (**D**) scRNA-Seq of CD5^+^ sorted T cells pooled from 5 mice for each treatment. UMAP plots for each treatment are shown for the expression of CD8a and Foxp3, demonstrating a reduction in Foxp3^+^ cells in the combination treated group. (**E**) Representative flow cytometry plots of Foxp3 versus Live/Dead viability dye from tumor cells. Cells were pregated on CD4^+^ T cells (left). Percentages of Foxp3^+^ that are Dead^+^ (right). (**F**) Differential analysis of gene expression by Tregs on selected groups analyzed by single-cell sequencing. Ordinary 1-way ANOVA with a Tukey’s multiple-comparison test was used. Symbols represent individual mice and lines represent averages ± SEM. *P < 0.05, ****P < 0.0001. Flow cytometry experiments were repeated at least twice with similar results. Gzmb, Granzyme B; FasL, Fas ligand.

Recruitment of CD4^+^Foxp3^+^ Tregs to the tumor bed is an important mechanism by which tumors evade immune surveillance (4). GITR engagement can downregulate the quantity and function of Tregs in tumors (12, 14, 16, 42). Moreover, CTX administration suppresses Tregs in tumors and the periphery (26). To determine whether the combination of CTX and GITR agonism affects intratumor Tregs, we first performed flow cytometry (Figure 5A) and observed reduced absolute numbers of Tregs in mice treated with the combination therapy compared with controls (Figure 5B). It is known that an elevated ratio of CD8^+^ T cells/Tregs is predictive of immunotherapy efficacy (43). Upon closer examination, we found that the ratio of CD8^+^ T cells/Tregs in the tumors was several-fold higher with the combination therapy than with either monotherapy (Figure 5C).

We further evaluated intratumor T cells by single-cell RNA sequencing (scRNA-Seq). T cells were isolated based on CD5 expression by FACS from B16 tumors of treated mice or controls. After appropriate quality controls, the cells were subjected to single-cell sequencing using the 10x Genomics platform coupled with TCR sequencing. We pooled intratumor T cells from mice treated with IgG, anti-GITR, CTX IgG, and CTX + anti-GITR. We included “hashtag” antibodies to determine intermouse variation. The uniform manifold approximation and projection for dimension reduction (UMAP) plots demonstrate that the combination therapy substantially decreased the number of Tregs and increased CD8^+^ T cells (Figure 5D and Supplemental Figure 1; supplemental material available online with this article; https://doi.org/10.1172/jci.insight.151035DS1). These data further confirm the changes observed by flow cytometry.

Next, we investigated how the combination treatment might lead to fewer intratumor Tregs. Immunotherapeutic interventions can reduce the number of intratumor Tregs by a variety of mechanisms, including activation-induced cell death (41). Using flow cytometry to characterize tumor-infiltrating Tregs, we observed that significantly more Tregs underwent cell death in the combination group versus control mice (Figure 5E). These findings indicate that Treg cell death might contribute to the overall efficacy of the combination treatment.

Our laboratory has previously shown that GITR engagement destabilizes Foxp3 and the suppressive Treg lineage (7). Using our single-cell sequencing data, clustering was performed to identify the intratumor Tregs in different treatment groups, and we ran differential expression analyses for selective suppressive and effector genes. As shown in Figure 5F, consistent with our previous observations, GITR agonism decreased the suppressive profile, endowing a T effector–like signature to Tregs characterized by upregulation of proinflammatory cytokines (IL-2 and IFN-γ) and cytolytic molecules (Gzmb and FasL). Interestingly, CTX alone induced Tregs that showed increased expression of tumor necrosis factor receptor superfamily members, Tnfrsf4 (OX40), Tnfrsf9 (4-1BB), Tnfrsf18 (GITR), and inducible T cell costimulatory molecule, which were recently described as activated Tregs that are selectively associated with poor outcomes in non-small cell lung cancer (44). Importantly, the addition of GITR agonist antibody to CTX profoundly abrogated this activated Treg phenotype. Furthermore, the remaining cells acquired an even higher cytolytic Teff-like phenotype.

### Combination of CTX and GITR agonism promotes cytotoxic terminally differentiated CD8^+^ T cells.

**Figure 6:**
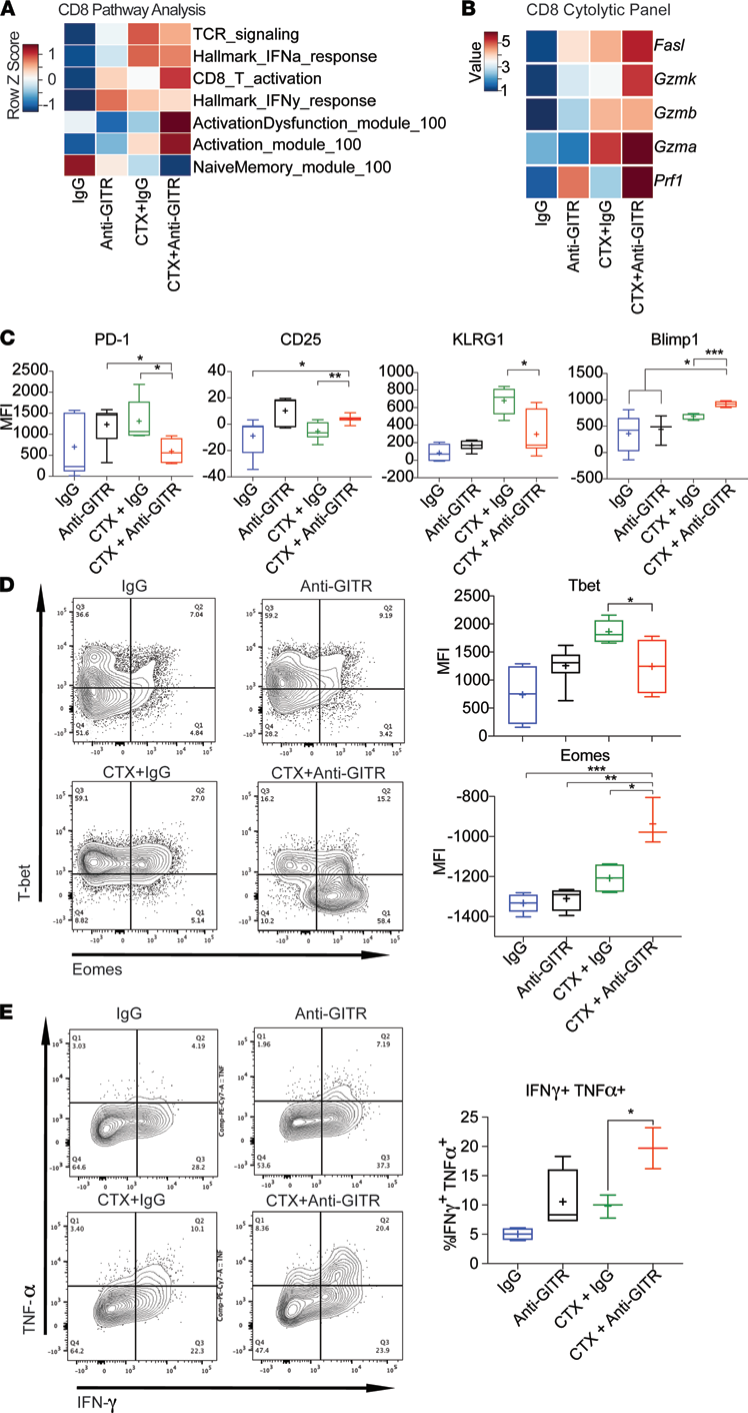
Combination of CTX and anti-GITR promotes effector CD8^+^ T cell fitness. Cohorts of mice were implanted with B16. On day 8, CTX was injected. The next day, mice were treated with anti-GITR antibody or IgG. Additional mice were implanted with B16 on day 8; on day 15 mice were treated with anti-GITR or IgG. After 1 week, all cohorts of mice were sacrificed, and single-cell suspensions were prepared. Single-cell suspensions were stained with anti-CD5 T cells and hashtag multiplex antibodies. The cells were FACS sorted for CD5^+^ and next-generation TCR coupled with 10x Genomics sequencing. (**A**) Pearson correlations of CD8^+^ cells from scRNA-Seq between treatments (columns) and expression of predefined gene signatures demonstrating increased T cell activation in the combination-treated groups. (**B**) Heatmap of selected cytolytic genes on CD8^+^ T cells. (**C** and **D**) Mice were treated as described above, and 10 days after anti-GITR treatment single-cell suspensions were prepared from tumors. Cells were analyzed by flow cytometry. (**C**) MFI of PD-1, CD25, KLRG1, and Blimp-1 gated on CD8^+^ T cells. (**D**) Representative flow cytometry plots of T-bet versus Eomes on a CD8^+^ T cell gate (left); average MFI of T-bet and Eomes per treatment group (right). (**E**) Mice were treated as shown above and single-cell suspensions from tumors of mice were stimulated with PMA/ionomycin. After 6 hours, intracellular cytokine staining analysis was performed by flow cytometry. Representative flow plots (left). Percentage of TNF^+^IFN-γ^+^ per treatment (right). Box-and-whisker plots of 3–6 mice/group are shown where bounds of the box represent the 25th and 75th percentiles, the line represents the median, the plus sign represents the mean, and whiskers represent the minimum and maximum values. *P < 0.05, **P < 0.01, ***P < 0.001. Flow cytometry experiments were repeated at least twice with similar results.

Given that the combination of CTX and GITR agonist antibody decreased intratumor Tregs and decreased Treg functionality, we next examined whether the combination increased functionality of tumor-infiltrating CD8^+^ T cells. First, we examined the overall landscape of intratumor CD8^+^ T cells by pathway analysis from the scRNA-Seq, described in detail in Methods. We used unsupervised clustering and then applied a predefined set of genes based on biological priors (see Methods) to define the CD8 cluster. We found that CD8^+^ T cells from the combination therapy group had a signature of higher activation (Figure 6A). Accordingly, scRNA-Seq showed increased transcription of a cytolytic program consisting of upregulation of granzymes, perforin, and FasL with the combination therapy, suggesting a highly activated state (Figure 6B and Supplemental Figure 2). Given the enhanced cytolytic profile with combination therapy, we further characterized the activation state of the CD8^+^ T cells at the protein level. Flow cytometry from single-cell suspensions prepared from tumors of treated mice demonstrated that intratumor CD8^+^ T cells from mice treated with combination therapy had an enhanced activation and terminal differentiation phenotype characterized by PD-1^lo^CD25^hi^KLRG1^lo^Blimp1^hi^ staining compared with treatment with CTX alone (Figure 6C) (45). Moreover, a subset of tumor-infiltrating CD8^+^ T cells were Eomes^hi^T-bet^lo^ (Figure 6D). Interestingly, this phenotype is associated with both an antigen-experienced, highly activated state as well as an exhausted/dysfunctional state of these cells (46, 47). To address this complexity, we stimulated single-cell suspensions, derived from tumors of treated mice, ex vivo with PMA/ionomycin and examined cytokine expression. The CD8^+^ T cells treated with combination therapy showed higher levels of TNF-α and IFN-γ, indicating that they were functional and not exhausted (Figure 6E). In conclusion, we found that the combination of CTX and GITR engagement promoted the expansion of differentiated, highly cytotoxic intratumor CD8^+^ T cells.

### Combination of CTX and anti-GITR constricts the TCR repertoire with expansion of highly activated CD8^+^ T cells.

**Figure 7:**
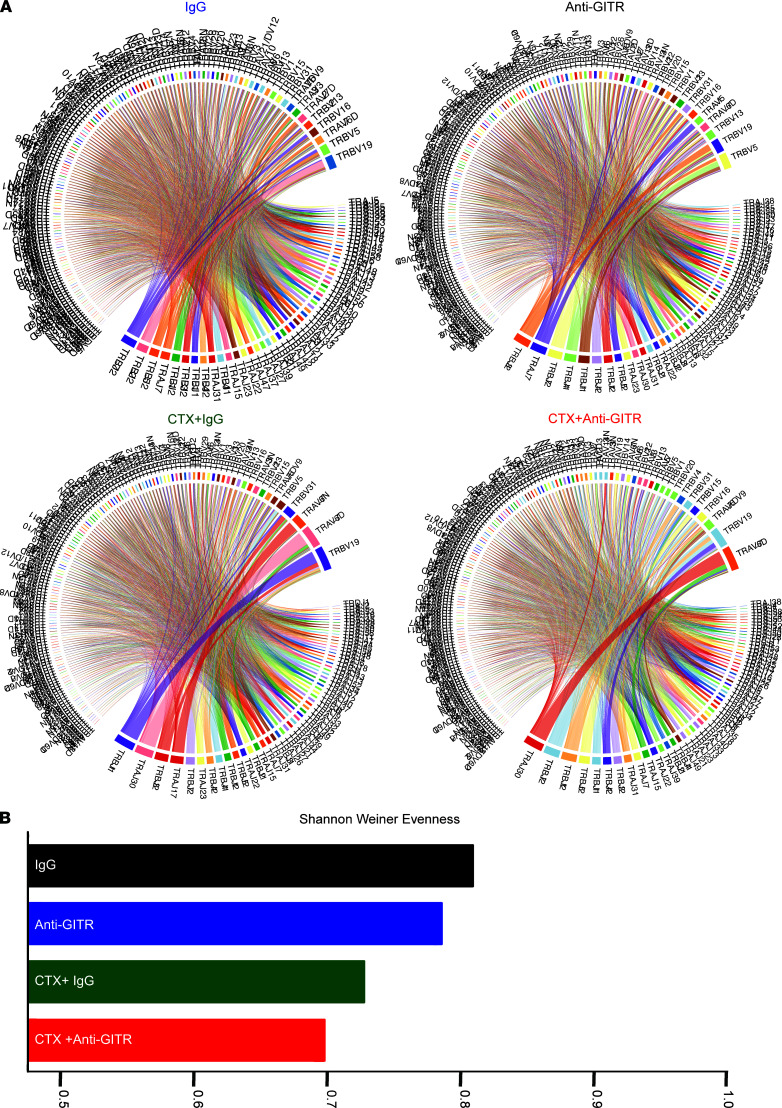
Combination therapy decreases the TCR repertoire. Cohorts of mice were implanted with B16. On day 8, CTX was injected. The next day, mice were treated with anti-GITR antibody or rat IgG. Additional cohorts of mice were injected with B16 on day 8; on day 15 mice were injected with anti-GITR or IgG. After 1 week, all cohorts of mice were sacrificed, and single-cell suspensions were prepared. Asynchronous B16 challenge was needed for the CTX groups a week apart given the lack of immune infiltrates caused by chemotherapy treatment. Single-cell suspensions were stained with anti-CD5 and hashtag multiplex antibodies. The cells were sorted by FACS based on CD5^+^, and next-generation TCR coupled with 10x Genomics sequencing was performed. (**A**) Circos plot displaying rearranged VDJ segments by treatment group, demonstrating an increased clonality in the combination-treated group. (**B**) Shannon-Weiner diversity evenness score for individual treatment groups.

**Figure 8:**
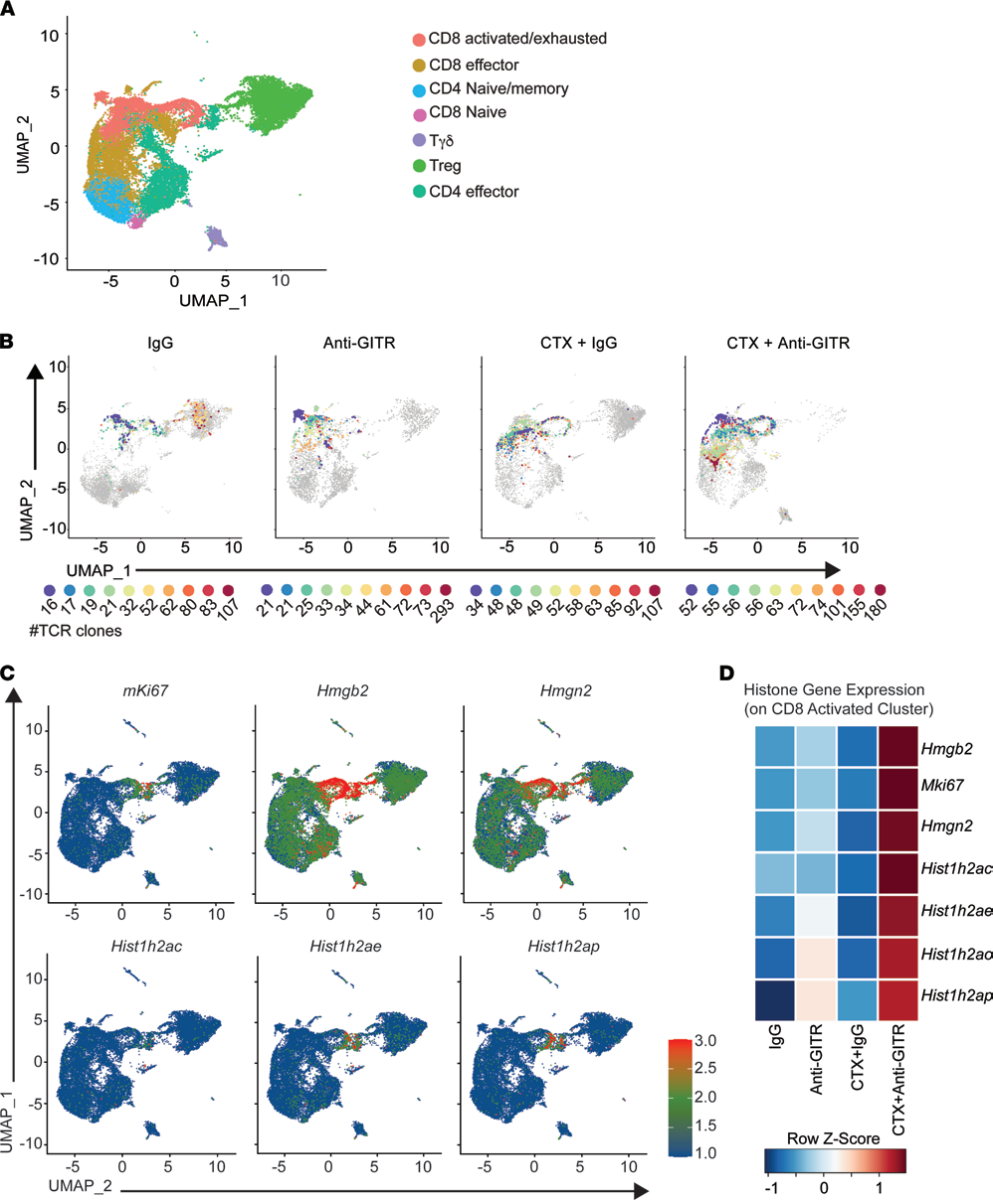
Combination therapy decreases the TCR repertoire by promoting clonal expansion of highly activated CD8^+^ T cells. Cohorts of mice were implanted with B16. On day 8, CTX was injected. The next day, mice were treated with anti-GITR antibody or rat IgG. Additional cohorts of mice were injected with B16 on day 8; on day 15 mice were injected with anti-GITR Ab or IgG. After 1 week, all cohorts of mice were sacrificed, and single-cell suspensions were prepared. Asynchronous B16 challenge was needed for the CTX groups a week apart given the lack of immune infiltrates caused by chemotherapy treatment. Single-cell suspensions were stained with anti-CD5 and hashtag multiplex antibodies. The cells were sorted by FACS based on CD5^+^, and next-generation TCR coupled with 10x Genomics sequencing was performed. (**A**) UMAP displaying different clusters. (For a list of genes included in each subclusters refer to Supplemental Table 2.) (**B**) UMAP showing gene expression of the top 10 clones present in each treatment. Number below each colored circle represents the total number of cells in each clone. (For clone sequences refer to Supplemental Table 1.) (**C**) UMAP on selected cell cycle and proliferation genes. (**D**) Heatmap of selected cell cycle and proliferation genes per condition.

The effects of novel immunotherapy regimens on the TCR repertoire and on the phenotype of tumor-reactive clones are still open questions. Immune-perturbing interventions may lead to diversification or clonal expansion of the TCR repertoire (48). To address this, we obtained paired TCR-sequencing data that could be linked to the aforementioned scRNA-Seq data. The advantage of this approach is that TCR identity and phenotype can be simultaneously studied at the single-cell level. First, we asked how the combination of CTX and GITR agonism affects the entire TCR repertoire. Circos plots of VDJ-rearranged TCR segments are shown in Figure 7A. As expected for settings where there is expansion of antigen-specific T cells, combination therapy yielded a population of CD8^+^ cells that showed reduced TCR diversity (i.e., increased clonal expansion). These results were supported by calculating a Shannon-Weiner evenness diversity score, which demonstrated increased clonal expansion with the combination therapy (Figure 7B). Examination of the top 10 most frequent clones in the CTX + anti-GITR treatment group showed a shift toward expansion of activated/exhausted CD8^+^ T cells in the defined UMAP (Figure 8, A and B; Supplemental Figure 3; and Supplemental Tables 1 and 2). We assigned a definition of T cell clusters supervised on lineage and functional markers on the aggregate (49, 50) (Figure 8A and Supplemental Figure 4; see Methods). Interestingly, many of the top 10 most frequent clones in the IgG and CTX plus IgG groups were Foxp3^+^ Tregs, indicating that an important role of anti-GITR therapy is eliminating the tumor- and CTX-associated expansion of Tregs.

Most of the expanded CD8^+^ T cell clones in the CTX + anti-GITR group clustered to a region in the UMAP visualized as a loop-like structure, which is indicative of active proliferation of these CD8^+^ T effectors. Unsupervised clustering of a population in the CD8 cluster of the CTX + anti-GITR group that included 10 of the most overrepresented clones revealed numerous histone- and chromatin-associated transcripts, which indicated vigorous cell cycling. Indeed, we observed that Ki67, histones, and chromatin-associated transcripts were strongly expressed. The selected genes mapped exclusively to the “loop” region rich in CD8^+^ T cells from the CTX + anti-GITR group (Figure 8C). To investigate this phenotype more closely, we generated a gene list and subsequent pathway analysis associated with the proliferation marker Ki67 and confirmed previous observations of highly cycling cell populations (Supplemental Figure 5 and Supplemental Table 3). Heatmap analysis of transcripts from cells derived from different treatments confirmed high gene expression levels exclusively in the CTX + anti-GITR group, indicating that combination treatment resulted in a highly proliferative CD8^+^ T cell burst (Figure 8D).

It is conceivable that the phenotype and clonal expansion of T cell subsets in a bulk analysis of a pooled cohort of mice could be driven by nonrepresentative outliers. To address this, we labeled cell suspensions from individual mice with a “hashtag” antibody before pooling and cell sorting (51). After sequencing and analysis, cells derived from each mouse can be segregated in silico. The majority of clones and phenotypes were consistent between mice that received equivalent treatment (Supplemental Figures 4 and 5 and Supplemental Table 4). The top 5 clones in the CTX + anti-GITR group were in the highly activated region of CD8^+^ T cells in all 5 mice. Overall, these experimental approaches identified TCRs with a highly activated/cytolytic phenotype in mice treated with CTX + anti-GITR. Future experiments will determine the specificity and avidity of the clones that promoted potent antitumor immunity, which could facilitate engineering of T cells that can be incorporated in adoptive T cell transfer protocols.

## Discussion

In this study, we rationally combined the alkylating chemotherapeutic CTX with GITR agonist antibodies, which led to potent and durable antitumor responses in several clinically relevant models, including MPC-11 plasmacytoma and the poorly immunogenic B16 melanoma. We observed that CTX treatment was associated with the upregulation of GITR on the surface of T cell subsets in a dose-dependent manner. This treatment was also associated with preferential Treg depletion and Teff expansion. The expanded Teffs consisted of highly activated, tumor-infiltrating, oligoclonal CD8^+^ T cells.

Chemotherapy plus immunotherapy combinations are becoming increasingly common in clinical oncology practice, following recent FDA approvals in non-small cell lung cancer (52–54) and triple-negative breast cancer (55). Homeostatic proliferation of antigen-specific Teffs is an important outcome of chemotherapeutic interventions, such as CTX. CTX induces lymphopenia followed by homeostatic proliferation of tumor-specific Teffs (24–26). At high doses, CTX promotes profound lymphodepletion that can create immunological “space.” During reconstitution from lymphopenia, T cells acquire an activated phenotype, upregulating molecules such as GITR, and tumor-reactive clones preferentially expand (22). This is a primary reason why high-dose CTX is included in conditioning regimens for adoptive cell transfer protocols and chimeric antigen receptor T cell therapy (56).

The mechanisms by which CTX acts in our combination therapy are likely multiple. Lymphodepletion on its own is insufficient to explain the efficacy because total-body irradiation at a dose that B16 is radioresistant to does not work in combination with anti-GITR. The data showing Pmel-1 antigen–specific T cell proliferation specifically in TDLNs of mice treated with CTX and anti-GITR support the hypothesis that CTX induces tumor death and release of the Pmel antigen for Pmel-1 T cell recognition. Further investigations are currently underway to examine why CTX, but not gem, which also slows tumor growth as a monotherapy and thus presumably elicits tumor cell death directly, uniquely combines with anti-GITR to augment tumor immunity.

An important mechanism by which tumors avoid immune elimination is the recruitment and activation of Foxp3^+^ Tregs, and the presence of these cells is an indicator of poor prognosis for patients with solid tumors (4, 5). Tregs suppress antitumor immune responses by a variety of mechanisms, including acting as an IL-2 sink to reduce the availability of IL-2 for effector cells and the secretion of immunosuppressive cytokines, such as TGF-β and IL-10 (4). Highly suppressive Tregs are found in both mouse and human tumors, and a major focus is to develop strategies to mitigate the presence and function of these cells. Anti–CTLA-4 (57) and anti-CCR4 (58) are 2 such approaches, but neither have proved effective for the majority of patients. Engaging GITR is an effective strategy to reduce or deactivate the suppressive function of Tregs and promote antitumor immunity. In this study, we demonstrated by both flow cytometry and scRNA-Seq that the combination of CTX and GITR agonist antibodies profoundly depletes intratumor Tregs. Closer examination revealed that Treg elimination occurred by induced cell death of hyperactivated Tregs. Interestingly, this is similar to the known cell death of Tregs observed in autoimmunity (59, 60). Other reports have described similar hyperactivation and potent antitumor immunity when engaging members of the TNF receptor family, such as OX40 (61, 62). The Tregs that failed to succumb to anti-GITR–induced cell death showed an increased Teff signature, characterized by upregulation of Teff and cytolytic markers. This observation is consistent with previous data from our laboratory showing that GITR agonism abolishes Treg suppressive function and destabilizes their phenotype to become effector like (7, 63). This “Teff-like” phenotype on Tregs stimulated with GITR agonist antibodies was more pronounced in cohorts of mice treated with CTX; perhaps the upregulation of surface GITR after CTX treatment underlies this observation (Figure 1). Future studies will establish if “Teff-like” Tregs possess actual antitumor functionality.

Previous mouse studies showed that GITR-induced depletion of Tregs is not sufficient to generate significant antitumor immunity, and effective engagement of GITR on Teffs is also needed (64, 65). Here we found that the combination of CTX and GITR agonism further promoted highly activated, cytolytic CD8^+^ T cells. More specifically, CD8^+^ T cells isolated from tumors of mice treated with combination therapy showed downregulation of KLRG1, consistent with improved antitumor activity (66). We also observed upregulation of Blimp1 in CD8^+^ T cells, suggestive of a terminally differentiated state (67). Most notably, CD8^+^ T cells from mice treated with combination CTX + GITR agonist antibodies produced higher levels of TNF-α and IFN-γ. These cells were more activated and had a less dysfunctional phenotype, characterized by high levels of CD25 and low levels of PD-1. Our analysis also revealed that a subset of CD8^+^ T cells from tumors of mice treated with combination therapy showed upregulation of Eomes and downregulation of T-bet. It remains unclear whether this particular phenotype is associated with tumor growth or regression because results from prior work across different models are conflicting (45–47, 66, 67); nevertheless, Eomes seems to be expressed at the end stages of the cytolytic program (68). Therefore, in addition to the effects on clonal expansion of tumor antigen–specific T cells, our data indicate that combination therapy with CTX and GITR engagement promotes highly cytolytic, terminally differentiated CD8^+^ T cells.

Recent advances in sequencing technology enable the detailed study of TCR repertoires in response to immunotherapy (69). As a result, TCR diversity has been suggested as a prognostic biomarker for monitoring immune responses when evaluating novel immunotherapies (70–72). An open question remains as to whether diversification of TCR repertoires or clonal proliferation underlies favorable outcomes in immunotherapy. It stands to reason that better outcomes might be associated with a diverse starting TCR repertoire, which contains multiple clones targeting multiple antigens that can efficiently eliminate tumors and prevent antigen escape variants (38, 73–75). However, the response to treatment is expected to be associated with the expansion of a few high-affinity clones that possess robust effector functionality (37, 76–80). Our results showed that, indeed, a reduced TCR repertoire with expansion of highly activated clones was associated with control of tumor growth and/or regression. We demonstrated that combination therapy promoted clones with high expression of genes involved in cell cycling and mitosis. Among these genes are a series of histone and chromatin-associated proteins associated with a vigorous proliferative burst (81–83). Based on the immunomodulatory and tumor-cytotoxic effects of CTX and the stimulatory effects of GITR, it was not surprising that high-affinity TCR clones were expanded after the combination treatment. CTX-induced lymphodepletion and subsequent homeostatic proliferation can promote expansion of higher affinity TCR clones (84). We believe the TCR data are extremely useful for future work that can yield potential therapeutic benefit, and we are currently investigating the specificity of the expanded clones, examining whether they recognize shared or unique tumor antigens. Based on this, we will ultimately evaluate whether the TCRs specific for tumor tissue–restricted antigens might be adopted for engineering into adoptive cell therapy approaches.

Taken together, CTX and GITR agonism potently controlled tumor growth in several clinically relevant animal models. Mechanisms associated with the control of tumor growth included CTX-induced tumor cell death, clonal expansion of highly active and terminally differentiated CD8^+^ T cells, and depletion of Tregs by activation-induced cell death. The potency of this combination therapy in mice with poorly immunogenic tumors warrants further investigation in the clinical setting. Indeed, the combination of the anti-GITR antibody TRX-518 with CTX and PD-1 blockade was the focus of a phase Ib/IIa trial in immunotherapy-refractory solid tumors (NCT03861403), but data are not yet available on the safety or efficacy of the triplet combination.

## Methods

### Mice and tumor cell lines.

C57BL/6J mice (6- to 8-week-old females) were obtained from The Jackson Laboratory. Pmel-1 TCR transgenic mice (Thy1.1) were originally a gift from N. Restifo (NIH, Bethesda, Maryland, USA) (41). All mice were bred at MSKCC. The B16-F10 (B16) mouse melanoma line was originally obtained from I. Fidler (MD Anderson Cancer Center, Houston, Texas, USA) and passaged intradermally in mice several times to ensure tumor growth. MPC-11 cells were obtained from ATCC. A lethal dose of 1 × 10^5^ B16, and 5 × 10^5^ MPC-11, cells was injected.

### Monoclonal antibodies and drug treatment.

Anti-GITR (DTA-1 clone) and IgG isotype control (rat IgG2b, anti-keyhole limpet hemocyanin) was obtained from Bio X Cell. For all treatments, 1 mg anti-GITR or IgG was administered. Cyclophosphamide monohydrate (MilliporeSigma) mixed in sterile PBS was administered. Clinical-grade gem was obtained from the MSKCC Pharmacy and diluted in PBS to the appropriate dilution. Whole-body irradiation was given as a single dose of 6 Gy. Both chemotherapies were administered as single doses intraperitoneally.

### Antibodies, FACS analysis, and cell sorting.

Antibodies used for flow cytometry analysis are listed in Supplemental Table 5 and were used according to the manufacturer’s instructions with the recommended buffers. The Vybrant CFDA SE Cell Tracer kit (CFSE), DAPI, and the LIVE/DEAD Fixable Aqua Dead Cell Stain kit were obtained from Invitrogen and used according to the manufacturer’s instructions. Sampled tumors were mechanically dissociated to obtain a single-cell suspension. Erythrocytes were removed using ACK lysing buffer (Invitrogen). Lymphocytes were clarified from tumors using 40% Percoll (GE Healthcare) gradient centrifugation. Before staining, cells were treated with saturating anti-CD16/CD32 (BD) in staining buffer (2% bovine serum albumin and 10 mM EDTA in PBS) on ice for 15 minutes. Staining of surface antigens was performed in staining buffer on ice for 40 minutes. All intracellular staining was conducted using the Foxp3 Fixation/Permeabilization Buffer (eBioscience) according to the manufacturer’s instructions. For intracellular cytokine staining, single-cell suspension from tumors were incubated at 37°C with 20 ng/mL PMA (MilliporeSigma) and 1 μg/mL ionomycin (MilliporeSigma) in Happy T Cell Media (RPMI 1640 supplemented with 10% FCS, 1× nonessential amino acids, 1 mM sodium pyruvate, 2 mM l-glutamine, and 50 µM β-mercaptoethanol) for 8 hours with 10 μg/mL monensin and 1X GolgiPlug (BD). Before staining, cells were treated with saturating anti-CD16/CD32 (BD) in staining buffer (2% bovine serum albumin and 2 mM EDTA in PBS) on ice for 15 minutes. Staining of surface antigens was performed in staining buffer on ice for 40 minutes. All intracellular staining was conducted using BD Cytoperm/Cytofix reagents (BD). Flow cytometry was performed on a flow cytometer (LSRII; BD). FlowJo software (version 8.6.2; Tree Star, Inc.) was used for all flow cytometry analysis. FACS was conducted on a cell sorter (FACSDiva; BD).

### Adoptive transfer experiments.

Tumor-bearing mice received 2 × 10^6^ CFSE-labeled CD8^+^ Pmel-1 cells intravenously via tail vein. CD8^+^ T cells were purified (<98% pure) from pooled spleens and lymph nodes from Pmel-1 mice (41) by positive selection using CD8a (Ly-2) microbeads (Miltenyi Biotec) according to the manufacturer’s instructions. Purified cells were stained in PBS with 10 μM CFSE at 37°C for 10 minutes and quenched in complete media. The cells were washed twice in PBS before transfer. Five days later, single-cell suspensions were prepared from draining lymph nodes and analyzed by flow cytometry as described above.

### scRNA-Seq.

Cohorts of 5 mice were injected with B16. On day 8, CTX was injected. The next day, mice were injected with anti-GITR antibody or IgG. Additional cohorts of mice were injected with B16 on day 8; on day 15 mice were injected with DTA or IgG. After 1 week, all cohorts of mice were sacrificed, and single-cell suspensions were prepared. Asynchronous B16 challenge was needed for the CTX groups a week apart given the lack of immune infiltrates caused by chemotherapy treatment. Single-cell suspensions were simultaneously stained with anti-CD5 PE (BD) and TotalSeq anti-mouse hashtag antibodies (C0301, C0302, C0303, C0304, C0305; BioLegend). The cells were FACS sorted based on CD5^+^, and next-generation TCR coupled with 10x Genomics sequencing was performed.

### scRNA-Seq data generation and processing.

Single-cell sequencing data were aligned to the Genome Reference Consortium Mouse Build 38 (mm10) using Cell Ranger (v3.1.0; 10x Genomics) to obtain T cell clonotypes, feature barcoding, and gene expression profiles associated with individual single cells. Each data type was matched to create a unique molecular identifier matrix, and cells were filtered out based on 3 metrics: (i) cells with fewer than 200 detectable genes, (ii) cells with more than 2500 detectable genes, and (iii) cells that had less than 5% percentage of counts related to mitochondrial genes. Data normalization, principal component analysis, and subsequent UMAP were performed on the data set using R package Seurat v.3.1.1 (https://github.com/satijalab/seurat). The differential expression comparisons were generated using the DESeq2 package with selected genes (FDR < 0.05). t-Distributed stochastic neighbor embedding plots were constructed using PartekFlow software.

### Supervised annotation of single cells.

After filtering, we created subclusters of cells using the Louvain algorithm (85, 86). We classified each subcluster to a predefined cell type by performing a supervised analysis based on a list of known marker genes (CD8 activated: *CD8a*^+^,*CD8b1*^+^ *Prf1*^+^, *Nkg7*^+^, *Gzmb*^+^, *GzmK*^+^, *CD4*^–^, *FoxP3*^–^; CD8: *CD8a*^+^, *CD8b1*^+^, *Perf1*^–^, *Slamf7*^–^ *CD4*^–^; Treg: *CD4*^+^, *IL2RA*^+^, *FoxP3*^+^, *CD8a*^–^, *TNFRSF4*^+^; CD4: *CD4*^+^, *CD8a*^–^, *CD8b*^–^, *CD28*^+^, *FoxP3^–^*; CD8 naive: *Sell*^+^, *Tcf7*^+^, *CD8a*^+^, *CCR7*^+^, *CD4*^–^; Tgd: *Tcrg^–^v6*^+^, *Trdv4*^+^, *IL17a*^+^, *Tcrg^–^C1*^+^, *Trdc*^+^, *CD8a*^–^, *CD4*^–^; CD4 naive: *CD4*^+^ *Sell*^+^ *FoxP3*^–^, *CCR7*^+^); 97% of the cells were distinctly annotated using this method, and the remaining cells were reviewed manually. We verified specific cell types using signature analysis for functional annotations (87, 88). Signature analysis per sample for a given subcluster was scored as the mean normalized expression across all genes listed for that signature.

### TCR clonality.

Clonality for each sample was calculated individually using Shannon-Weiner evenness. This was then compared across the different treatment conditions.

### Data availability.

Data presented in this study are available within the paper and its supplemental material. Publicly available data sets used in this study were available at National Center for Biotechnology Information Gene Expression Omnibus accession number GSE182292 (https://www.ncbi.nlm.nih.gov/geo/query/acc.cgi?acc=GSE182292).

### Statistics.

All statistical analyses were calculated using GraphPad Prism software. Statistical differences between 2 experimental groups were determined by the unpaired 2-tailed Student’s *t* test. Statistical differences between more than 2 groups were calculated using a 1- or 2-way ANOVA followed by a post hoc Tukey’s multiple comparisons test. Statistical differences between Kaplan-Meier survival curves were calculated using a log-rank (Mantel-Cox) test. The α value was set at 0.05 for all analyses, and *P* values less than 0.05 were considered statistically significant.

### Study approval.

All mouse procedures were performed in accordance with the Institutional Animal Care and Use Committee guidelines at MSKCC under an approved protocol.

## Author contributions

We determined the coauthorship by evaluating the key contribution of each author, and the first coauthorship was determined by the fact that the 2 first authors have been key contributors to the findings described in this manuscript. DH, ABW, TM, JDW, AC, ADC, SB, and ANH, GAR developed the concepts and discussed experiments. DH, ABW, RM, TM, and JDW wrote the manuscript. DH, ABW, ADC, SB, and NS performed and analyzed animal model experiments, flow cytometry experiments, and functional assays. DH, LMBM, and AC performed bioinformatics analysis. NS, LH, and CL provided technical assistance.

## Supplementary Material

Supplemental data

## Version 1 10/22/2021

Electronic publication
