# Supplementary material for: Cyclophosphamide enhances the antitumor potency of GITR engagement by increasing oligoclonal cytotoxic T cell fitness
Source: JCI Insight. 2021 Oct 22;6(20):e151035. doi: 10.1172/jci.insight.151035 (PMC8564916; doi:10.1172/jci.insight.151035)
Supplement: Supplemental data [file jciinsight-6-151035-s182.pdf]

Conflict of interest: TM and JDW are coinventors on patent applications related to CD40 and in situ vaccination (PCT/US2016/045970), filed by MSKCC. JDW is a consultant for Amgen, Astellas, AstraZeneca, Bayer, Bicara Therapeutics, Boehringer Ingelheim, Bristol Myers Squibb, Chugai, Daiichi Sankyo, Dragonfly, Eli Lilly, F Star, Georgiamune, Idera, Imvaq Therapeutics, Kyowa Kirin, Merck, Psioxus, Recepta, Sellas, Surface Oncology, Syndax, Syntalogic Pharmaceuticals, Truvax, Trishula, and Werewolf Therapeutics. JDW receives grant/research support from Bristol Myers Squibb and Sephora. JDW is a consultant and has equity in Tizona Pharmaceuticals, Imvaq Therapeutics, BeiGene, Linneaus Therapeutics, Apricity, Arsenal IO, Georgiamune, Trieza, Maverick Therapeutics, and Ascentage Pharma and is an inventor on patents: Xenogeneic (Canine) DNA Vaccines (US 7,556,805), alphavirus replicon particles expressing TRP2 (PCT/US2010/030423), myeloid-derived suppressor cell (MDSC) assay (PCT/US2013/027475), Newcastle disease virus for cancer therapy (US 10,251,922), vaccinia virus mutants useful for cancer immunotherapy (with TM, US 16/612,127), anti-CD40 agonist mAb fused to monophosphoryl lipid A (MPL) for cancer therapy (with TM, US 15/750,496), antigen-binding proteins targeting melanoma differentiation antigens and uses thereof (US 16/388,245) anti–PD-1 antibody (with TM, US 10,323,091), anti-CTLA4 antibodies (with TM, US 10,144,779), and anti-GITR antibodies and methods of use thereof (with TM, US 10,155,818; US 10,280,226). TM is a consultant for ImmunOs Therapeutics and Pfizer; is cofounder of and equity holder in Imvaq Therapeutics; receives research funding from Bristol Myers Squibb, Surface Oncology, Ikena Oncology, Infinity Pharmaceuticals, Peregrine Pharmaceuticals, Adaptive Biotechnologies, Leap Therapeutics, and Aprea Therapeutics; and is an inventor on patent applications related to oncolytic viral therapy (US 16/980,282), alpha virus–based vaccine (with JDW, PCT/US2010/030423), neo antigen modeling (with JDW, US 16/478,818), and OX40 (US 62/262,371). DH and SB have received royalties from Agenus. DH and TM are coinventors on patent applications related to OX40 antibodies (PCT/US2016/064794), filed by MSKCC. DH is an inventor on patent applications related to contraceptive vaccines (PCT/US2004/006216 and PCT/US2003/029257). ABW is a consultant for Iovance, Novartis, Nanobiotix, and Jo'Ann Medical and has received speaking honoraria from LG Chem.

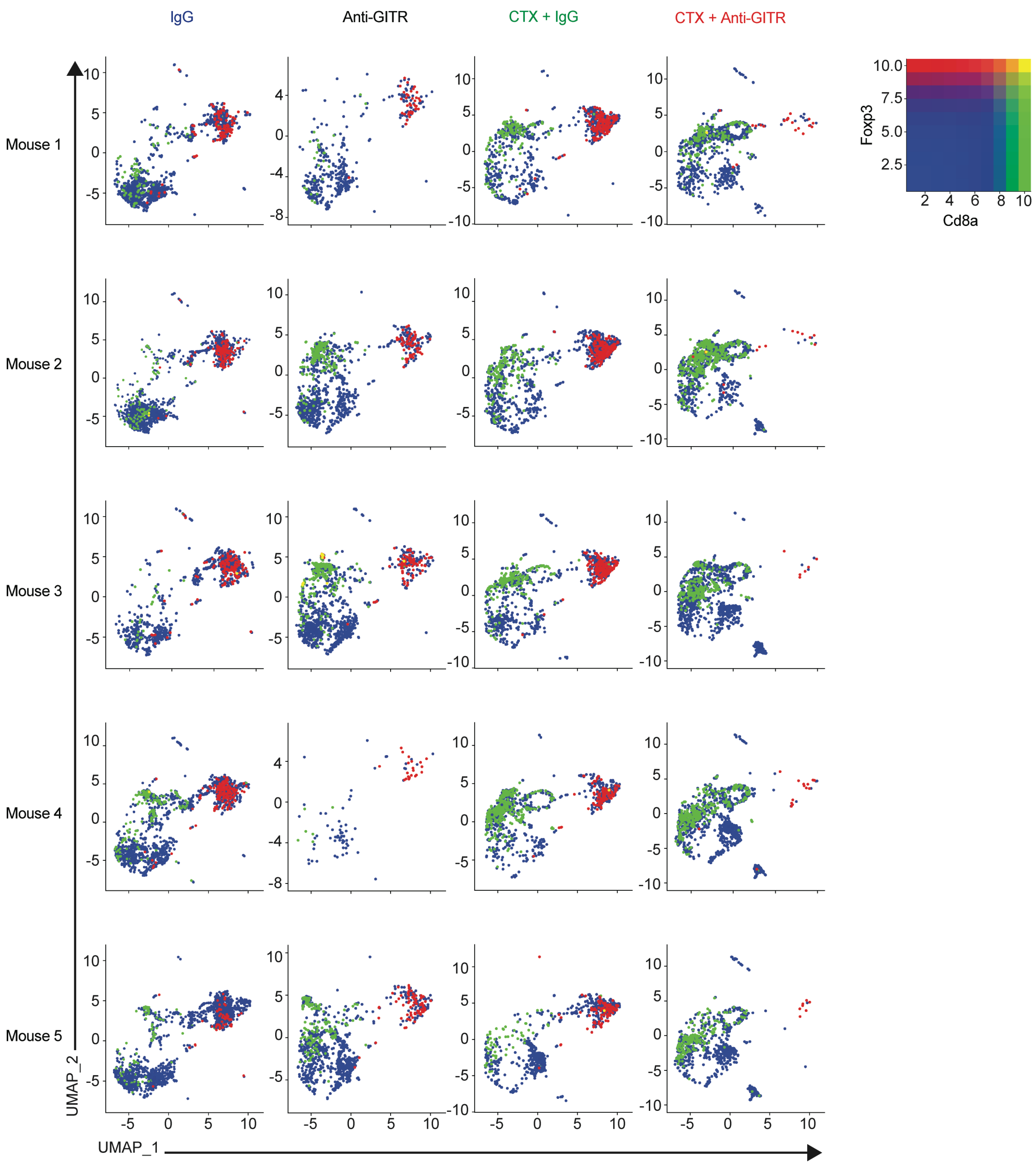

Supplemental Figure 1: Contribution of individual mice to the decrease in intratumoral Tregs and increases CD8:Treg ratio. As in previous experiments, mice were implanted with B16 and treated with CTX on day 8 and anti-GITR or control IgG on day 9. Additional cohorts of mice were implanted with B16 on day 8, on day 15 mice were treated with anti-GITR or IgG. After 1 week, all cohorts of mice were sacrificed, and single cell suspensions were prepared from tumors. UMAP plots for each treatment showing individual mice deconvoluted from each analysis are shown for the expression of CD8a and Foxp3.

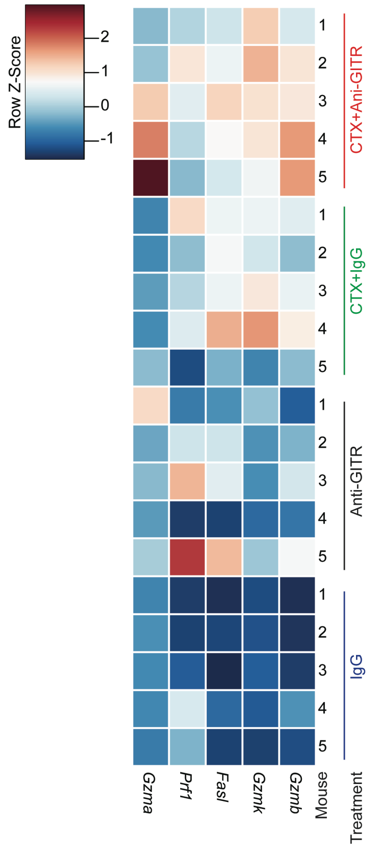

Supplemental Figure 2: Contribution of individual mice to the cytolytic program of CD8+ T cells. Cohorts of mice were implanted with B16. On day 8, CTX was injected. The next day, mice were treated with anti-GITR antibody or IgG. Additional cohorts of mice were implanted with B16 on day 8, on day 15 mice were treated with anti-GITR antibody or IgG. After 1 week, all cohorts of mice were sacrificed, and single cell suspensions were prepared. Asynchronous B16 challenge was needed for the CTX groups a week apart given the lack of immune infiltrates caused by chemotherapy treatment. Single cell suspensions were stained with anti-CD5 T cells and hashtag multiplex antibodies. The cells were FACs sorted based on CD5+ and next next-generation TCR coupled with 10X sequencing was performed. Heat map of selected cytolytic genes on CD8+ T cells from mice in each treatment group is shown here with an increase in expression in the combination treated group.

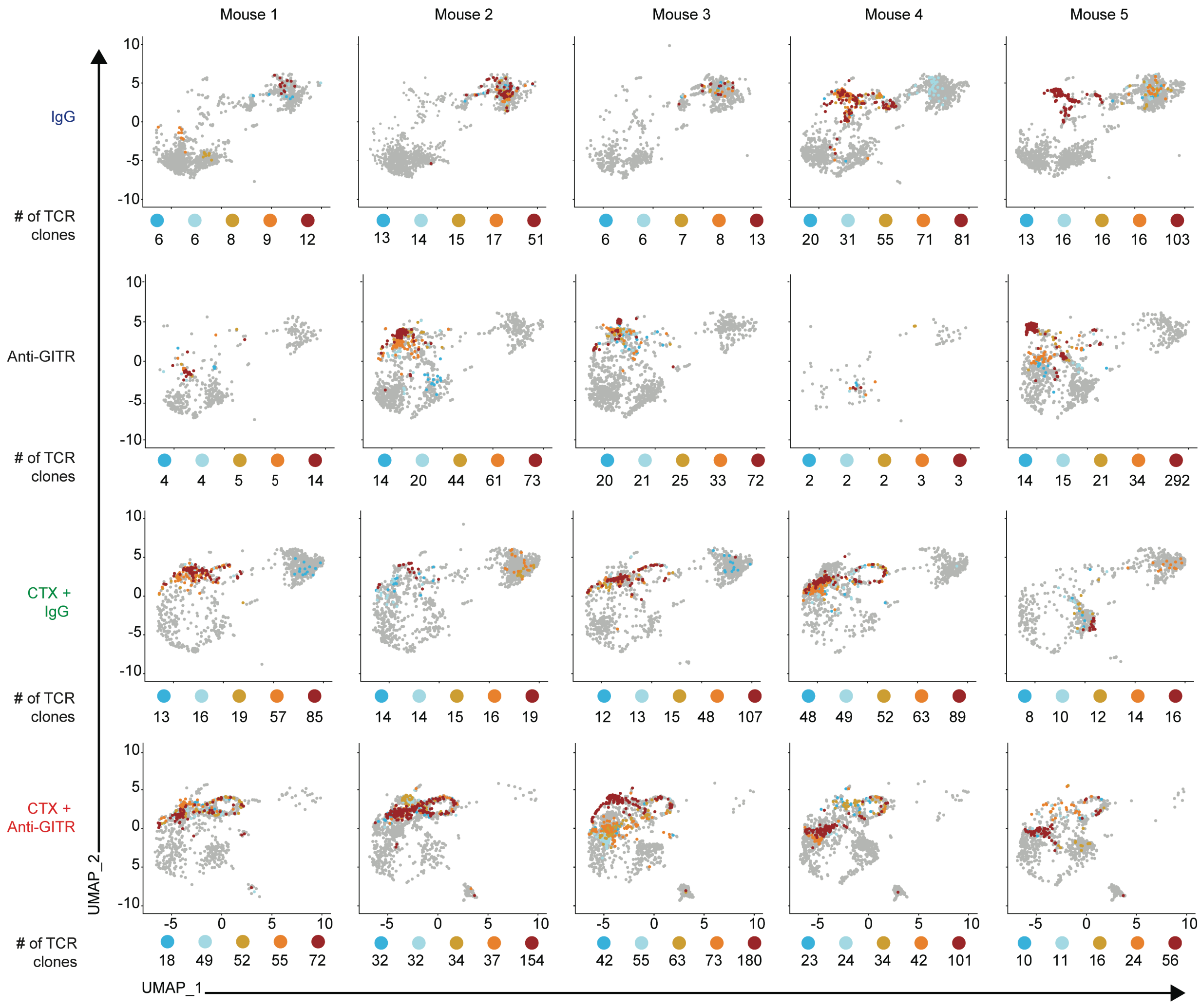

Supplemental Figure 3: Number of overrepresented TCR clones in individual mice per treatment. Cohorts of mice were implanted with B16. On day 8, CTX was injected. The next day, mice were treated with anti-GITR antibody or IgG. Additional cohorts of mice were implanted with B16 on day 8, on day 15 mice were treated with anti-GITR antibody or IgG. After 1 week, all cohorts of mice were sacrificed, and single cell suspensions were prepared. Asynchronous B16 challenge was needed for the CTX groups a week apart given the lack of immune infiltrates caused by chemotherapy treatment. Single cell suspensions were stained with anti-CD5 T cells and hashtag multiplex antibodies. The cells were FACs sorted based on CD5+ and next next-generation TCR coupled with 10X sequencing was performed. UMAPs showing the top 5 overrepresented clones deconvoluted from individual mice per treatment. Number below each colored circle represents the total number of cells in each clone.

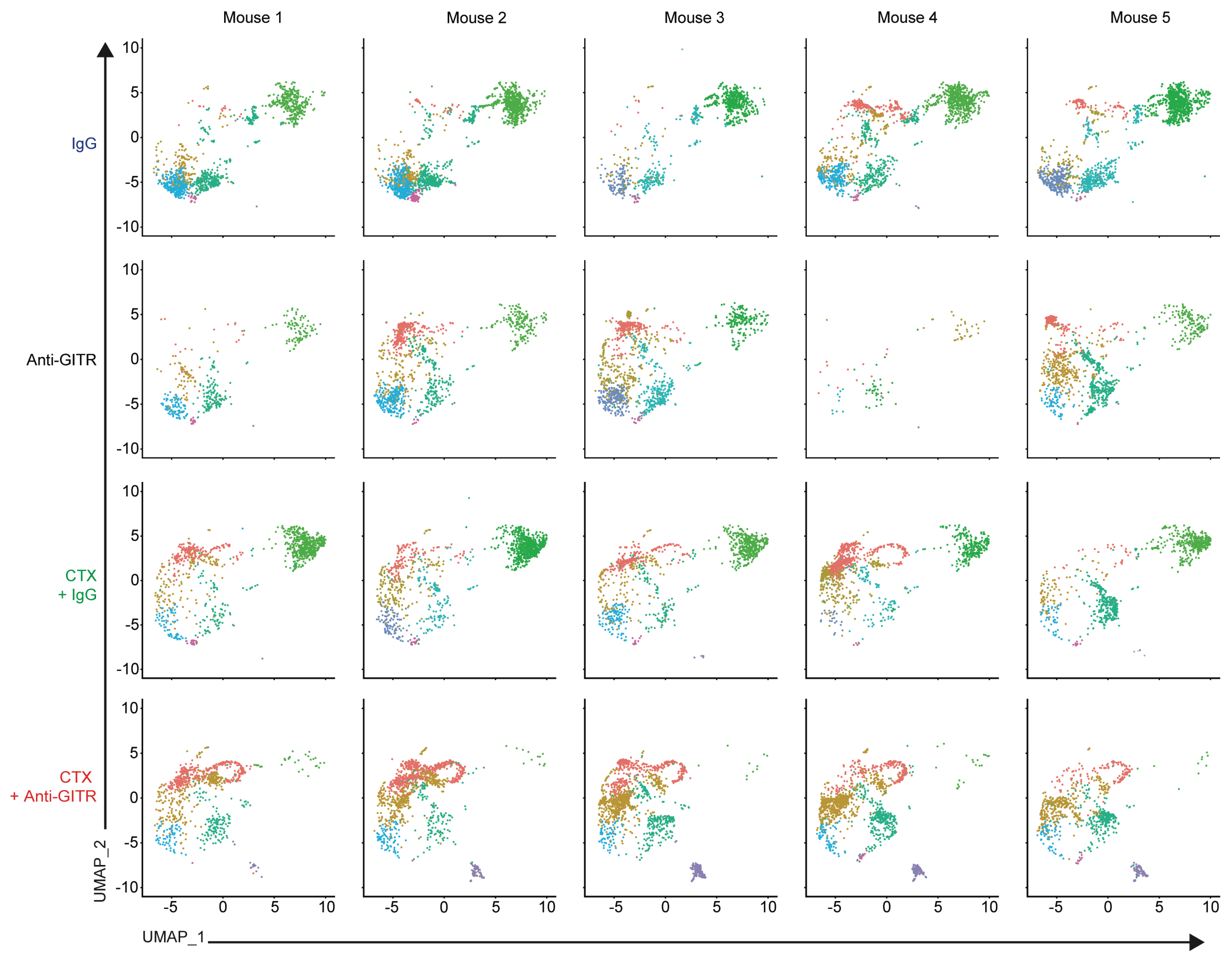

Supplemental Figure 4: Cluster distribution in individual mice per treatment. Cohorts of mice were implanted with B16. On day 8, CTX was injected. The next day, mice were treated with anti-GITR antibody or rat IgG. For the same experiment, additional cohorts of mice were implanted with B16 on day 8, on day 15 mice were treated with DTA or IgG. After 1 week, all cohorts of mice were sacrificed, and single cell suspensions were prepared. Asynchronous B16 implant was needed for the CTX groups a week apart given the lack of immune infiltrates caused by chemotherapy treatment. Single cells suspensions were stained for anti-CD5 T cells and hashtag multiplex antibodies. The cells were FACs sorted based on CD5+ and next next-generation TCR coupled with 10X sequencing was performed. UMAP showing the population of cells deconvoluted from individual mice per treatment.

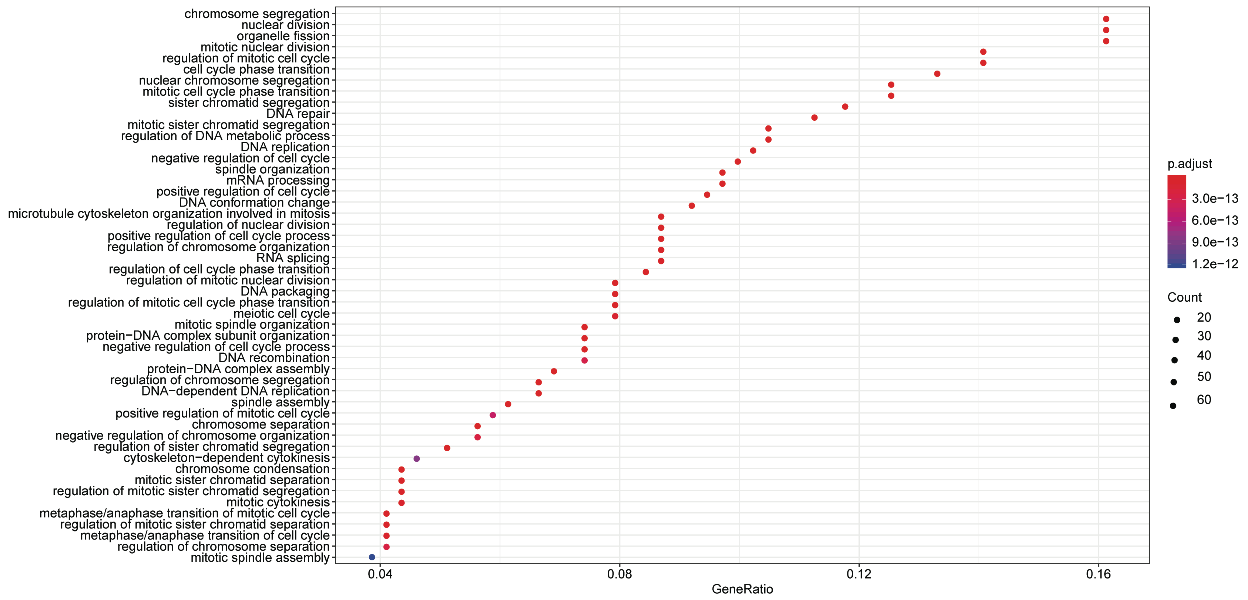

Supplemental Figure 5: Pathway analysis of CD8 Ki67 cells. Dot plot of enriched terms from GO overrepresentation analysis in the context of selected CD8 Ki67 cluster. The circle represents the counts of enriched genes in the signatures. P value is shown in the legend.

Supplemental Table 1: Sequences of the top 10 over-represented clones

**IgG**
TRA:CALGEDYSNNRLTL; TRB:CASSQTRDWAYEQYF, 107
TRA:CAVRTPDYANKMIF; TRB:CASSLNTGQLYF, 83
TRA:CAAKDYSNNRLTL; TRB:CASSVGLGEDTQYF, 80
TRA:CAATDYSNNRLTL; TRB:CASSLELGGLEQYF, 62
TRA:CAVSRGSALGRLHF; TRB:CSSSQRGRGERLFF, 52
TRA:CILRVSGGNNKLTF; TRB:CASSLSGGWYAEQFF, 32
TRA:CAAEDYSNNRLTL; TRB:CASSQVQGSAETLYF, 21
TRA:CALGYGSSGNKLIF; TRB:CASSQEGSQNTLYF, 19
TRA:CAAKGNTGNYKYVF; TRB:CASSQEWGGEQYF, 17
TRA:CALELDSNYQLIW; TRB:CASSRLGGGQNTLYF, 16
**Anti-GITR**
TRA:CSAKDYSNNRLTL; TRB:CASSQVQGANERLFF, 293
TRA:CAAKDYSNNRLTL; TRB:CASSQDKGANTEVFF, 73
TRA:CAMRDPGTQVVGQLTF; TRA:CALSDDYSNNRLTL; TRB:CASSPPGSSETLYF, 72
TRA:CALSEPNSNNRIFF; TRA:CALDMNYNQGKLIF; TRB:CASSFGTGQLYF, 61
TRA:CAMERDYSNNRLTL; TRB:CASSLELGGTEQFF, 44
TRA:CALNGNYGSSGNKLIF; TRA:CALRGTTASLGKLQF, TRB:CASSGGDQNTLYF, 34
TRA:CSASNPDYSNNRLTL; TRB:CASSRTGGSQNTLYF,33
TRA:CALSPDYSNNRLTL; TRB:CASSQVPDPNSDYTF, 25
TRA:CAAMDYSNNRLTL; TRB:CASSQGQGADTEVFF, 21
TRA:CALSDDYSNNRLTL; TRB:CASSQGQGSQNTLYF, 21
**CTX + IgG**
TRA:CALGDTNAYKVIF; TRA:CALVDLSGSGGKLTL; TRB:CASTPLSSNTEVFF, 107
TRA:CALFHDTNAYKVIF; TRB:CASSIRGVTEVFF,92
TRA:CAAKDYSNNRLTL; TRB:CASSQVGNNERLFF, 85
TRB:CASSRTGFTEVFF, 63
TRA:CAAGGDTNAYKVIF; TRA:CALGADSGGSNAKLTF; TRB:CAWSGGSAETLYF, 58
TRA:CALGHDTNAYKVIF; TRB:CASSYSGNTLF,52
TRA:CALGDPWQGGRALIF; TRB:CGARGQASAETLYF, 49
TRA:CALGGDTNAYKVIF; TRB:CASTPLSSNTEVFF,48
TRA:CALSSDTNAYKVIF; TRB:CASSRTGFEVFF, 48
TRA:CAMREASGGSNAKLTF; TRB:CASTPTANSDYTF, 34

**CTX + Anti-GITR**
TRA:CAMEQDYSNNRLTL; TRB:CASSLELGGLEQYF, 180
TRA:CALGHDTNAYKVIF; TRB:CASTINYAEQFF,155
TRA:CAASTLNSNNRIFF; TRB:CASTWTGYAEQFF, 101
TRA:CAVSDSNYQLIW; TRB:CASRTGSDYTF, 74
TRA:CADTGNYKYVF; TRA:CALGGDTBAYKVIF; TRB:CASSIQGSNTEVFF, 72
TRA:CALEGDYANKMIF; TRB:CASSENSYEQYF, 63
TRA:CAVMDSNYQLIW; TRB:CASSLVGTGNAEQFF,56
TRA:CAVSPPDYSNNRLTL; TRB:CASSSGGQDTQYF; TRB:CGAREGGGYEQYF,56
TRA:CAASWSGGSNYKLTF; TRB:CASSQDRGYEQYF, 55
TRA:CALGHDTNAYKVIF; TRA:CALGGDTNAYKVIF; TRB:CASSLLGSQNTLYF, 52

Supplemental Table 2: Uniquely expressed genes in each cell type

p_val	avg_logFC	pct.1	pct.2	p_val_adj	cluster	gene
0	1.100761271	0.556	0.115	0	Cd8 Activated	Trgv2
0	1.038199885	0.942	0.354	0	Cd8 Activated	Cd8a
0	1.017141622	0.398	0.153	0	Cd8 Activated	Stmn1
0	0.99188134	0.584	0.168	0	Cd8 Activated	Prf1
0	0.976310222	0.628	0.185	0	Cd8 Activated	Gzmk
0	0.94119479	0.368	0.095	0	Cd8 Activated	Ube2c
0	0.93175756	0.992	0.727	0	Cd8 Activated	Nkg7
0	0.931188736	0.952	0.408	0	Cd8 Activated	Cd8b1
0	0.83552887	0.937	0.441	0	Cd8 Activated	Cxcr6
0	0.826706751	0.775	0.265	0	Cd8 Activated	Lag3
0	0.823090617	0.287	0.056	0	Cd8 Activated	Birc5
0	0.75611558	0.881	0.428	0	Cd8 Activated	Pdcd1
0	0.753574993	0.473	0.123	0	Cd8 Activated	Ccl3
0	0.748780893	0.679	0.326	0	Cd8 Activated	Klrd1
0	0.745932673	0.945	0.676	0	Cd8 Activated	Ccl5
0	0.722013905	0.591	0.202	0	Cd8 Activated	Klrc1
0	0.716038555	0.922	0.632	0	Cd8 Activated	Sh2d2a
0	0.714613546	0.279	0.049	0	Cd8 Activated	Ccnb2
0	0.703803347	0.661	0.195	0	Cd8 Activated	Ccl4
0	0.638135184	0.937	0.673	0	Cd8 Activated	Ctsd
0	0.626794054	0.62	0.237	0	Cd8 Activated	Tox
0	0.598751948	0.29	0.071	0	Cd8 Activated	Cks1b
0	0.577686823	0.636	0.294	0	Cd8 Activated	Lgals3
0	0.574421637	0.955	0.666	0	Cd8 Activated	Anxa2
0	0.565420631	0.938	0.544	0	Cd8 Activated	Ctsw
0	0.563175246	0.69	0.328	0	Cd8 Activated	Il10ra
0	0.554204118	0.47	0.16	0	Cd8 Activated	Rgs16
0	0.522432762	0.758	0.349	0	Cd8 Activated	Litaf
0	0.521522974	0.606	0.252	0	Cd8 Activated	Klrk1
0	0.516865377	0.837	0.483	0	Cd8 Activated	Efhd2
0	0.512672048	0.973	0.787	0	Cd8 Activated	Sub1
0	0.512286613	0.921	0.652	0	Cd8 Activated	Txn1
0	0.49984547	0.489	0.147	0	Cd8 Activated	Arsb
0	0.488340199	0.993	0.869	0	Cd8 Activated	Lgals1
0	0.463336965	0.392	0.117	0	Cd8 Activated	Havcr2

0	0.445408401	0.646	0.301	0	Cd8 Activated	Casp3
0	0.441469791	0.917	0.654	0	Cd8 Activated	Zyx
0	0.435731172	0.999	0.941	0	Cd8 Activated	AW112010
0	0.43248169	0.256	0.044	0	Cd8 Activated	AC163354.1
0	0.42805917	0.545	0.2	0	Cd8 Activated	Fasl
0	0.412477107	0.512	0.179	0	Cd8 Activated	Ifng
0	0.410748105	0.449	0.162	0	Cd8 Activated	Chst12
0	0.401209731	0.254	0.044	0	Cd8 Activated	Tcrg-C2
0	0.397841203	0.378	0.119	0	Cd8 Activated	Plek
0	0.391981079	0.286	0.061	0	Cd8 Activated	Wdr95
0	0.37746055	0.994	0.94	0	Cd8 Activated	Cd3g
0	0.369041464	0.299	0.072	0	Cd8 Activated	Gpd2
0	0.358938107	0.979	0.856	0	Cd8 Activated	Myl12a
0	0.342380048	1	0.997	0	Cd8 Activated	Tmsb4x
0	0.329233262	0.274	0.072	0	Cd8 Activated	Gm17745
0	0.322205036	0.278	0.074	0	Cd8 Activated	Tmem163
0	0.320606403	0.995	0.945	0	Cd8 Activated	Cd3e
1.04E-307	0.436181471	0.694	0.362	1.67E-303	Cd8 Activated	Sh2d1a
1.11E-303	0.431377735	0.715	0.385	1.78E-299	Cd8 Activated	Ptms
6.77E-301	0.372589255	0.978	0.813	1.08E-296	Cd8 Activated	S100a11
7.24E-300	0.424264626	0.563	0.26	1.16E-295	Cd8 Activated	Serpinb6a
1.12E-299	0.325107833	0.989	0.924	1.79E-295	Cd8 Activated	Rbm3
2.69E-296	0.507600876	0.938	0.765	4.31E-292	Cd8 Activated	S100a6
1.45E-293	0.441863384	0.832	0.528	2.33E-289	Cd8 Activated	Cst7
2.47E-282	0.275886841	0.995	0.928	3.95E-278	Cd8 Activated	Cox8a
7.33E-278	0.340408666	0.49	0.206	1.17E-273	Cd8 Activated	Sema4a
6.83E-275	0.439951529	0.265	0.076	1.10E-270	Cd8 Activated	Klre1
1.55E-273	0.392305711	0.362	0.129	2.48E-269	Cd8 Activated	Nr4a2
8.02E-273	0.780468634	0.272	0.082	1.29E-268	Cd8 Activated	Pclaf
3.78E-266	0.389212181	0.761	0.449	6.06E-262	Cd8 Activated	Gng2
1.25E-263	0.332451466	0.313	0.103	2.00E-259	Cd8 Activated	Eomes
3.17E-260	0.43397913	0.58	0.289	5.08E-256	Cd8 Activated	Dut
7.21E-243	0.677703817	0.653	0.411	1.16E-238	Cd8 Activated	Hmgn2
1.48E-242	0.294750638	0.991	0.863	2.38E-238	Cd8 Activated	Thy1
1.78E-240	0.354970509	0.877	0.605	2.86E-236	Cd8 Activated	B4galnt1
1.89E-232	0.357631271	0.907	0.662	3.02E-228	Cd8 Activated	Id2
4.16E-231	0.308815077	0.471	0.213	6.67E-227	Cd8 Activated	Stard3nl
1.81E-226	0.294446513	0.252	0.079	2.91E-222	Cd8 Activated	Cenpw
5.52E-220	0.314057221	0.947	0.766	8.84E-216	Cd8 Activated	Cox5a

4.23E-219	0.306417158	0.955	0.797	6.78E-215	Cd8 Activated	Cox5b
1.91E-217	0.458366065	0.643	0.387	3.07E-213	Cd8 Activated	Selenoh
5.38E-216	0.266156364	0.256	0.083	8.62E-212	Cd8 Activated	Cd38
3.98E-212	0.340584425	0.767	0.488	6.38E-208	Cd8 Activated	Ech1
1.16E-210	0.278996422	0.359	0.145	1.86E-206	Cd8 Activated	Plscr1
4.09E-209	0.276857275	1	0.991	6.56E-205	Cd8 Activated	Ppia
5.75E-209	0.330492204	0.629	0.351	9.22E-205	Cd8 Activated	Cox17
1.10E-207	0.262215064	0.992	0.928	1.76E-203	Cd8 Activated	Clic1
1.73E-205	0.403380875	0.656	0.392	2.77E-201	Cd8 Activated	Dap
1.03E-204	0.271123104	0.99	0.918	1.65E-200	Cd8 Activated	Calm1
1.93E-204	0.656506403	0.444	0.226	3.09E-200	Cd8 Activated	Cenpa
1.15E-198	0.256090532	0.384	0.162	1.84E-194	Cd8 Activated	Atp8b4
3.68E-196	0.306830317	0.885	0.658	5.91E-192	Cd8 Activated	Gdi2
1.44E-194	0.260234711	0.336	0.135	2.31E-190	Cd8 Activated	Adam8
6.74E-193	0.278076511	0.421	0.19	1.08E-188	Cd8 Activated	Slamf7
4.14E-192	0.262022799	0.985	0.786	6.63E-188	Cd8 Activated	Ms4a4b
1.14E-186	0.319569848	0.624	0.363	1.82E-182	Cd8 Activated	Acadl
7.00E-184	0.323260203	0.804	0.564	1.12E-179	Cd8 Activated	Calm3
9.61E-183	0.354882839	0.965	0.827	1.54E-178	Cd8 Activated	Arl6ip1
1.51E-181	0.284364088	0.369	0.161	2.42E-177	Cd8 Activated	Cd160
2.37E-179	0.308747139	0.454	0.225	3.80E-175	Cd8 Activated	Serpinb6b
9.28E-175	0.318833179	0.401	0.191	1.49E-170	Cd8 Activated	Abcb9
2.76E-174	0.36987087	0.918	0.777	4.42E-170	Cd8 Activated	Hmgb1
1.30E-173	0.324142049	0.678	0.424	2.09E-169	Cd8 Activated	Nap1l1
1.81E-170	0.268021926	0.533	0.285	2.90E-166	Cd8 Activated	Ndfip2
7.06E-164	0.253429619	0.969	0.828	1.13E-159	Cd8 Activated	Lsp1
1.28E-162	0.263836104	0.422	0.206	2.06E-158	Cd8 Activated	Armc7
6.19E-160	0.259317013	0.417	0.205	9.92E-156	Cd8 Activated	Ctsc
6.72E-160	0.265221556	0.894	0.706	1.08E-155	Cd8 Activated	Atp5f1
4.86E-158	0.370667475	0.856	0.668	7.79E-154	Cd8 Activated	Ran
6.84E-158	0.282100646	0.557	0.315	1.10E-153	Cd8 Activated	Acot7
1.34E-157	0.288051203	0.847	0.627	2.15E-153	Cd8 Activated	Prdx1
1.78E-153	0.265154535	0.618	0.366	2.85E-149	Cd8 Activated	Uqcc2
2.61E-153	0.273694796	0.654	0.408	4.19E-149	Cd8 Activated	Atpif1
7.58E-146	0.393052541	0.534	0.32	1.22E-141	Cd8 Activated	Plac8
1.47E-145	0.252134953	0.401	0.201	2.36E-141	Cd8 Activated	Rpa2
1.06E-144	0.455882212	0.752	0.56	1.69E-140	Cd8 Activated	H2afv
6.69E-142	0.298517259	0.266	0.114	1.07E-137	Cd8 Activated	Racgap1
2.28E-141	0.298089299	0.308	0.142	3.66E-137	Cd8 Activated	Gm42031

6.06E-141	0.258825052	0.623	0.377	9.71E-137	Cd8 Activated	S100a4
2.36E-140	0.777108176	0.837	0.675	3.78E-136	Cd8 Activated	Hmgb2
8.76E-138	0.252694533	0.844	0.628	1.40E-133	Cd8 Activated	Rap1a
6.62E-137	0.264559278	0.647	0.408	1.06E-132	Cd8 Activated	Slc25a4
1.34E-136	0.301231773	0.583	0.357	2.14E-132	Cd8 Activated	Ptpn6
1.04E-135	0.257617308	0.718	0.475	1.66E-131	Cd8 Activated	Pstpip1
4.77E-131	0.258725862	0.814	0.599	7.64E-127	Cd8 Activated	Hsp90b1
5.52E-127	0.271923705	0.667	0.436	8.85E-123	Cd8 Activated	Trbc2
5.04E-113	0.265724483	0.653	0.44	8.08E-109	Cd8 Activated	Dbi
2.03E-110	0.322636166	0.301	0.154	3.26E-106	Cd8 Activated	Cks2
1.90E-97	0.28503127	0.37	0.214	3.04E-93	Cd8 Activated	Ube2s
2.31E-89	0.602940154	0.37	0.228	3.71E-85	Cd8 Activated	H2afx
7.21E-51	0.38448071	0.296	0.197	1.16E-46	Cd8 Activated	Tuba1b
4.86E-47	0.448295958	0.475	0.358	7.80E-43	Cd8 Activated	Tubb4b
6.29E-42	0.53885413	0.784	0.67	1.01E-37	Cd8 Activated	Tubb5
0	1.191536	0.835	0.398	0	Cd8	Ly6c2
0	0.880024493	0.596	0.295	0	Cd8	Plac8
0	0.859619459	0.858	0.411	0	Cd8	Cd8b1
0	0.773943016	0.551	0.188	0	Cd8	Gzmk
0	0.750007584	0.601	0.239	0	Cd8	Klrk1
0	0.745827171	0.901	0.677	0	Cd8	Ccl5
0	0.710102776	0.836	0.358	0	Cd8	Cd8a
0	0.691765249	0.524	0.204	0	Cd8	Klrc1
0	0.669603069	0.55	0.233	0	Cd8	Jaml
0	0.641235497	0.9	0.538	0	Cd8	Ctsw
0	0.616331836	0.981	0.719	0	Cd8	Nkg7
0	0.602589035	0.972	0.781	0	Cd8	Ms4a4b
0	0.519055001	0.444	0.175	0	Cd8	Slamf7
0	0.50782029	0.855	0.655	0	Cd8	Epsti1
0	0.436677016	0.648	0.319	0	Cd8	Klrd1
3.31E-250	0.620806184	0.439	0.211	5.31E-246	Cd8	Ifit3
7.70E-229	0.42042632	0.679	0.452	1.23E-224	Cd8	Dnajc15
7.16E-225	0.369416698	0.55	0.302	1.15E-220	Cd8	Evl
3.50E-220	0.370921996	0.357	0.16	5.61E-216	Cd8	Atp8b4
8.95E-218	0.364351777	0.909	0.781	1.43E-213	Cd8	Ms4a6b
1.53E-217	0.540079422	0.429	0.218	2.45E-213	Cd8	Usp18
1.18E-207	0.383615565	0.352	0.157	1.88E-203	Cd8	Cd160
2.93E-207	0.372728415	0.429	0.216	4.70E-203	Cd8	Fasl
5.02E-206	0.290503011	0.963	0.865	8.05E-202	Cd8	Thy1

7.80E-205	0.297824229	0.75	0.47	1.25E-200	Cd8	Cxcr6
2.68E-195	0.491949902	0.355	0.169	4.30E-191	Cd8	Ms4a4c
4.64E-175	0.310869136	0.314	0.142	7.43E-171	Cd8	St3gal6
1.51E-167	0.387409971	0.471	0.275	2.42E-163	Cd8	Ifi208
2.86E-164	0.302829491	0.4	0.208	4.58E-160	Cd8	Gramd3
4.39E-157	0.30388209	0.809	0.649	7.03E-153	Cd8	Sp100
7.12E-155	0.340296525	0.384	0.207	1.14E-150	Cd8	Armc7
1.17E-146	0.326293562	0.582	0.38	1.88E-142	Cd8	Itgb1
1.75E-136	0.313096264	0.826	0.673	2.81E-132	Cd8	Id2
3.38E-135	0.328735203	0.314	0.159	5.42E-131	Cd8	Ifit1bl1
1.35E-132	0.301738257	0.516	0.328	2.17E-128	Cd8	Ctla2a
3.66E-132	0.294846581	0.508	0.325	5.86E-128	Cd8	Sp110
7.52E-131	0.466140768	0.424	0.249	1.21E-126	Cd8	Cd7
7.68E-125	0.283368326	0.646	0.467	1.23E-120	Cd8	Gng2
1.55E-123	0.280336434	0.681	0.51	2.48E-119	Cd8	Efhd2
3.55E-121	0.312830199	0.46	0.29	5.69E-117	Cd8	Ifi213
1.87E-120	0.312339524	0.778	0.637	3.00E-116	Cd8	Bst2
1.25E-119	0.267443444	0.277	0.138	2.01E-115	Cd8	Gpr18
9.59E-119	0.257314727	0.825	0.667	1.54E-114	Cd8	Zyx
6.35E-117	0.507417638	0.607	0.456	1.02E-112	Cd8	Isg15
2.16E-114	0.269382747	0.288	0.15	3.47E-110	Cd8	Fam26f
1.18E-111	0.256883044	0.381	0.224	1.90E-107	Cd8	Sema4a
2.96E-110	0.272930844	0.293	0.156	4.74E-106	Cd8	Ifi214
6.30E-110	0.277239086	0.801	0.673	1.01E-105	Cd8	Txn1
1.83E-105	0.303446618	0.682	0.535	2.94E-101	Cd8	Zbp1
4.51E-105	0.376206583	0.39	0.242	7.23E-101	Cd8	Ifit1
1.33E-104	0.259804663	0.747	0.609	2.13E-100	Cd8	Psmb10
2.29E-103	0.323131428	0.662	0.509	3.67E-99	Cd8	Irf7
2.22E-100	0.40282127	0.475	0.323	3.55E-96	Cd8	Lgals3
5.03E-98	0.2987369	0.547	0.392	8.06E-94	Cd8	Ifi209
4.25E-97	0.272549615	0.785	0.661	6.82E-93	Cd8	Ifi47
2.55E-95	0.416139092	0.673	0.523	4.09E-91	Cd8	Gzmb
4.01E-92	0.250304523	0.327	0.194	6.42E-88	Cd8	Phf11c
2.15E-75	0.278699619	0.715	0.583	3.44E-71	Cd8	Hspe1
1.88E-70	0.259028437	0.404	0.279	3.01E-66	Cd8	Gbp2
8.78E-67	0.258002066	0.418	0.302	1.41E-62	Cd8	Mcm6
5.91E-65	0.276446744	0.556	0.433	9.48E-61	Cd8	Phf11b
2.29E-17	0.320280604	0.611	0.549	3.67E-13	Cd8	Hsp90aa1
1.00E-15	0.282111248	0.428	0.362	1.61E-11	Cd8	Dnajb1

0	2.208022423	0.952	0.219	0	Treg	Tnfrsf4
0	1.939777943	0.843	0.034	0	Treg	Foxp3
0	1.778113314	0.712	0.102	0	Treg	Cd74
0	1.710295443	0.878	0.349	0	Treg	Tnfrsf9
0	1.659525889	0.864	0.267	0	Treg	Ctla4
0	1.521867518	0.965	0.562	0	Treg	Tnfrsf18
0	1.381466488	0.841	0.226	0	Treg	Ikzf2
0	1.338833805	0.475	0.08	0	Treg	Klrg1
0	1.330049456	0.897	0.507	0	Treg	Glrx
0	1.327432588	0.637	0.101	0	Treg	Izumo1r
0	1.298177391	0.799	0.296	0	Treg	Ass1
0	1.293867688	0.479	0.075	0	Treg	Ccr8
0	1.287535712	0.514	0.039	0	Treg	Cd81
0	1.154552671	0.774	0.41	0	Treg	Ddit4
0	1.133126112	0.547	0.101	0	Treg	Il2ra
0	1.131532689	0.924	0.682	0	Treg	Sdf4
0	1.116880195	0.89	0.564	0	Treg	Pglyrp1
0	1.097332444	0.938	0.512	0	Treg	Capg
0	1.09437058	0.441	0.083	0	Treg	Bmyc
0	1.094113759	0.796	0.466	0	Treg	Odc1
0	1.084354058	0.795	0.38	0	Treg	Icos
0	1.045655544	0.817	0.481	0	Treg	Zfp36l1
0	0.98936367	0.842	0.476	0	Treg	Tigit
0	0.945718748	0.88	0.468	0	Treg	Itgb7
0	0.944212652	0.783	0.451	0	Treg	Dusp1
0	0.931410513	0.678	0.289	0	Treg	Gm2a
0	0.919755093	0.8	0.451	0	Treg	Itm2c
0	0.885525935	0.543	0.215	0	Treg	Itgav
0	0.872066711	0.787	0.418	0	Treg	Hif1a
0	0.869229144	0.892	0.657	0	Treg	Nfkbia
0	0.868562491	0.491	0.13	0	Treg	Ramp1
0	0.866273066	0.807	0.466	0	Treg	Pim1
0	0.864421808	0.452	0.16	0	Treg	Slc16a3
0	0.859375185	0.639	0.291	0	Treg	Ighm
0	0.852324395	0.654	0.305	0	Treg	Samsn1
0	0.839483154	0.959	0.808	0	Treg	Cd2
0	0.824373592	0.559	0.158	0	Treg	Tspan32
0	0.813873466	0.628	0.25	0	Treg	Psen2
0	0.806370546	0.354	0.028	0	Treg	Ebi3

0	0.790901054	0.987	0.933	0	Treg	Srgn
0	0.781867928	0.495	0.11	0	Treg	Maf
0	0.780720807	0.609	0.34	0	Treg	Ccr2
0	0.772950188	0.453	0.095	0	Treg	Snx9
0	0.771575293	0.322	0.033	0	Treg	Ecm1
0	0.767320491	0.971	0.871	0	Treg	Pkm
0	0.754060382	0.809	0.51	0	Treg	Cd5
0	0.740010581	0.511	0.212	0	Treg	Dgat1
0	0.734319238	0.504	0.2	0	Treg	Tnfrsf1b
0	0.724271482	0.469	0.2	0	Treg	H1f0
0	0.71823505	0.972	0.825	0	Treg	Ly6a
0	0.711663207	0.605	0.29	0	Treg	Snx20
0	0.711422104	0.446	0.121	0	Treg	Sdcbp2
0	0.711416617	0.985	0.849	0	Treg	Ifi27l2a
0	0.711138283	0.96	0.845	0	Treg	Vim
0	0.70728679	0.375	0.078	0	Treg	Arl5a
0	0.706998382	0.365	0.131	0	Treg	Bnip3
0	0.689102136	0.546	0.261	0	Treg	Bcl3
0	0.687135258	0.326	0.053	0	Treg	Matk
0	0.67908133	0.72	0.444	0	Treg	Crlf2
0	0.675915356	0.495	0.231	0	Treg	Fgl2
0	0.674450719	0.706	0.423	0	Treg	Isg20
0	0.670136473	0.465	0.194	0	Treg	Cebpb
0	0.663422933	0.394	0.113	0	Treg	Fam110a
0	0.656448281	0.389	0.099	0	Treg	Wls
0	0.655721116	0.701	0.463	0	Treg	M6pr
0	0.64915011	0.46	0.162	0	Treg	Ctsz
0	0.647282607	0.357	0.124	0	Treg	Ccrl2
0	0.646789854	0.995	0.965	0	Treg	Ly6e
0	0.64038399	0.642	0.391	0	Treg	Tmem123
0	0.631598602	0.596	0.345	0	Treg	Wnk1
0	0.628882855	0.553	0.154	0	Treg	Cd4
0	0.626670779	0.803	0.561	0	Treg	Cmtm7
0	0.625191839	0.456	0.188	0	Treg	Sdhaf1
0	0.615561923	0.408	0.179	0	Treg	Mxd1
0	0.606812161	0.27	0.026	0	Treg	Rapsn
0	0.606620263	0.627	0.364	0	Treg	Ubl3
0	0.602029399	0.478	0.206	0	Treg	Trp53i11
0	0.595239846	0.613	0.339	0	Treg	Mif4gd

0	0.59463223	0.652	0.394	0	Treg	Sat1
0	0.59045076	0.777	0.579	0	Treg	Ctsb
0	0.583312957	0.607	0.352	0	Treg	Cish
0	0.581648171	0.348	0.107	0	Treg	Snx18
0	0.581076606	0.623	0.363	0	Treg	Dusp5
0	0.569139366	0.908	0.821	0	Treg	Npc2
0	0.566779976	0.277	0.038	0	Treg	C1qtnf12
0	0.565115422	0.833	0.692	0	Treg	Pgk1
0	0.56456079	0.279	0.049	0	Treg	Lamc1
0	0.558431014	0.683	0.465	0	Treg	Sla
0	0.55188219	0.45	0.205	0	Treg	Crem
0	0.545117301	0.66	0.434	0	Treg	Pkp3
0	0.541337922	0.39	0.131	0	Treg	Tmem154
0	0.538881653	0.86	0.746	0	Treg	Gpx4
0	0.538839414	0.399	0.173	0	Treg	Il3ra
0	0.534300808	0.677	0.472	0	Treg	Ctss
0	0.531110061	0.931	0.853	0	Treg	Ifngr1
0	0.53106697	0.866	0.728	0	Treg	Rap1b
0	0.528366315	0.287	0.081	0	Treg	Lmna
0	0.52817243	0.786	0.583	0	Treg	Lrp10
0	0.522841285	0.637	0.414	0	Treg	Cyb5a
0	0.522553963	0.379	0.157	0	Treg	Serinc3
0	0.519165639	0.797	0.643	0	Treg	Ypel3
0	0.51867164	0.39	0.166	0	Treg	P2ry10
0	0.513621027	0.795	0.615	0	Treg	Samhd1
0	0.499147752	0.346	0.138	0	Treg	Hacd3
0	0.49503425	0.275	0.069	0	Treg	Aldoc
0	0.491130425	0.46	0.226	0	Treg	Gna15
0	0.465605758	0.26	0.073	0	Treg	Socs2
0	0.464218926	0.274	0.079	0	Treg	Rarg
0	0.460786163	0.755	0.603	0	Treg	Coro1b
0	0.459741693	0.936	0.841	0	Treg	Ndfip1
0	0.452897642	0.833	0.707	0	Treg	Cd37
0	0.451351525	0.318	0.112	0	Treg	Arhgap31
0	0.444273646	0.811	0.684	0	Treg	Rhog
0	0.44267523	0.998	0.995	0	Treg	H2-K1
0	0.440877799	0.907	0.804	0	Treg	Pnrc1
0	0.43498059	0.999	0.996	0	Treg	Actg1
0	0.431809547	0.998	0.994	0	Treg	H2-D1

0	0.430387579	0.968	0.918	0	Treg	S100a10
0	0.428387918	0.395	0.146	0	Treg	Selenop
0	0.422875712	0.949	0.891	0	Treg	Ptprcap
0	0.422285095	0.986	0.983	0	Treg	Fth1
0	0.392156909	0.961	0.92	0	Treg	Ldha
0	0.357002863	0.99	0.974	0	Treg	Shisa5
0	0.32752818	0.979	0.963	0	Treg	Fxyd5
0	0.305731719	0.978	0.956	0	Treg	Itm2b
4.58E-305	0.511000334	0.635	0.42	7.34E-301	Treg	Rhoh
4.75E-300	0.549382672	0.59	0.382	7.61E-296	Treg	Syngr2
5.22E-300	0.410023727	0.964	0.915	8.37E-296	Treg	Aldoa
2.35E-299	0.549948655	0.665	0.463	3.77E-295	Treg	Traf1
2.85E-298	0.532617113	0.78	0.605	4.57E-294	Treg	Gimap7
5.26E-295	0.52023352	0.78	0.585	8.43E-291	Treg	Tpi1
2.23E-290	0.483942589	0.935	0.882	3.58E-286	Treg	Ltb
6.89E-286	0.520212111	0.296	0.104	1.10E-281	Treg	Iigp1
9.26E-278	0.498367878	0.481	0.264	1.48E-273	Treg	Snx2
5.88E-272	0.473181951	0.784	0.628	9.43E-268	Treg	Jund
3.49E-270	0.41905388	0.281	0.101	5.59E-266	Treg	Sytl1
6.96E-267	0.522976394	0.5	0.287	1.12E-262	Treg	Mxd4
3.63E-266	0.457172905	0.414	0.201	5.81E-262	Treg	Retreg1
1.86E-260	0.546746133	0.562	0.357	2.98E-256	Treg	Rilpl2
3.54E-259	0.658464467	0.721	0.498	5.68E-255	Treg	Gzmb
3.22E-258	0.553406487	0.715	0.531	5.16E-254	Treg	Zfp36
4.02E-257	0.344344121	0.946	0.908	6.45E-253	Treg	Il2rg
2.05E-255	0.511093971	0.649	0.475	3.29E-251	Treg	Cd27
1.34E-253	0.497566232	0.527	0.322	2.14E-249	Treg	Lbh
5.46E-250	0.438704347	0.446	0.225	8.75E-246	Treg	Rora
6.26E-248	0.477568788	0.352	0.158	1.00E-243	Treg	Phlda1
2.87E-245	0.384278987	0.278	0.106	4.60E-241	Treg	Sypl
3.39E-245	0.45032335	0.547	0.329	5.43E-241	Treg	Rgs10
4.02E-245	0.735972684	0.289	0.114	6.44E-241	Treg	Mt1
8.98E-245	0.443215884	0.472	0.265	1.44E-240	Treg	Spint2
5.48E-242	0.430386978	0.772	0.643	8.79E-238	Treg	Tap1
9.33E-236	0.422598603	0.803	0.683	1.50E-231	Treg	Cytip
4.63E-235	0.431050042	0.285	0.116	7.42E-231	Treg	1700017B05Rik
2.07E-234	0.561629486	0.36	0.178	3.32E-230	Treg	Chchd10
1.01E-231	0.446388932	0.661	0.476	1.62E-227	Treg	Cd6
1.05E-231	0.444997584	0.265	0.102	1.68E-227	Treg	Nrip1

1.71E-230	0.454897825	0.544	0.352	2.74E-226	Treg	Ptpn7
2.32E-230	0.405462788	0.89	0.806	3.71E-226	Treg	Gapdh
8.96E-230	0.486525428	0.344	0.16	1.44E-225	Treg	Rgs2
2.75E-228	0.391529274	0.767	0.65	4.41E-224	Treg	Slc9a3r1
2.52E-227	0.432008002	0.298	0.128	4.03E-223	Treg	Slc25a19
9.63E-227	0.413882706	0.441	0.242	1.54E-222	Treg	Tmbim4
1.83E-226	0.313040562	0.927	0.884	2.93E-222	Treg	Ucp2
3.69E-225	0.43327909	0.4	0.212	5.91E-221	Treg	Unc119
1.34E-222	0.54942583	0.326	0.15	2.14E-218	Treg	Hilpda
1.18E-220	0.384977886	0.372	0.182	1.89E-216	Treg	Smco4
1.86E-220	0.314985772	0.876	0.807	2.97E-216	Treg	Gnb2
1.33E-209	0.430520868	0.555	0.377	2.13E-205	Treg	Rhof
2.67E-209	0.359430667	0.831	0.738	4.28E-205	Treg	Leprotl1
1.56E-208	0.404674896	0.875	0.807	2.50E-204	Treg	H2-T22
5.18E-201	0.294104095	0.878	0.813	8.30E-197	Treg	Rac1
1.43E-196	0.413015945	0.665	0.484	2.29E-192	Treg	Cxcr3
8.61E-196	0.386679297	0.385	0.199	1.38E-191	Treg	Ube2l6
1.24E-195	0.430002303	0.57	0.394	1.99E-191	Treg	Cdc42se2
3.93E-195	0.2662315	0.901	0.858	6.30E-191	Treg	Arf5
1.15E-194	0.527984154	0.381	0.203	1.84E-190	Treg	Gadd45b
1.77E-194	0.432594569	0.428	0.252	2.83E-190	Treg	Glipr1
4.85E-193	0.392426213	0.499	0.306	7.77E-189	Treg	Foxo1
2.72E-192	0.3246222	0.875	0.767	4.36E-188	Treg	Il2rb
5.80E-192	0.397278876	0.281	0.126	9.30E-188	Treg	Coro2a
2.38E-191	0.403480813	0.29	0.131	3.81E-187	Treg	Plk3
8.16E-191	0.40229042	0.331	0.163	1.31E-186	Treg	Fam129a
1.21E-190	0.337196176	0.729	0.585	1.93E-186	Treg	Vamp8
2.00E-189	0.346072324	0.804	0.696	3.21E-185	Treg	Gimap1
1.03E-186	0.402399802	0.319	0.156	1.65E-182	Treg	Ttc39c
9.63E-185	0.423853719	0.392	0.225	1.54E-180	Treg	Impa2
1.21E-183	0.398148002	0.376	0.202	1.95E-179	Treg	Galnt6
2.28E-181	0.477239331	0.334	0.173	3.65E-177	Treg	Got1
6.62E-181	0.318032689	0.262	0.11	1.06E-176	Treg	Arl5c
4.77E-179	0.384966605	0.445	0.27	7.65E-175	Treg	Eva1b
9.99E-177	0.370506837	0.655	0.509	1.60E-172	Treg	Hcls1
1.44E-176	0.422941813	0.669	0.503	2.31E-172	Treg	Rgs1
2.05E-176	0.377758451	0.584	0.427	3.28E-172	Treg	Glmp
6.13E-175	0.430548909	0.301	0.149	9.82E-171	Treg	Nfil3
3.80E-171	0.292732734	0.911	0.865	6.08E-167	Treg	Gpi1

1.72E-170	0.300757397	0.978	0.969	2.75E-166	Treg	Malat1
1.18E-166	0.391326059	0.366	0.203	1.89E-162	Treg	Nfkb2
2.20E-166	0.391574948	0.503	0.34	3.53E-162	Treg	Jak3
8.47E-165	0.259303919	0.921	0.867	1.36E-160	Treg	Tmbim6
6.14E-163	0.394639005	0.39	0.225	9.85E-159	Treg	Plec
1.43E-162	0.360959094	0.307	0.159	2.30E-158	Treg	Ergic1
6.96E-161	0.34371185	0.259	0.117	1.12E-156	Treg	Fam46c
1.97E-160	0.377113734	0.364	0.204	3.16E-156	Treg	Ubash3b
2.05E-160	0.357865633	0.272	0.132	3.29E-156	Treg	Mmd
7.11E-159	0.368342266	0.281	0.136	1.14E-154	Treg	Rrad
1.59E-158	0.35596159	0.501	0.339	2.54E-154	Treg	Pigx
1.23E-155	0.392332926	0.501	0.342	1.97E-151	Treg	Tspan13
1.51E-155	0.359585784	0.638	0.5	2.42E-151	Treg	Grap
1.16E-153	0.637801948	0.263	0.124	1.86E-149	Treg	Nr4a1
1.72E-153	0.37355717	0.588	0.439	2.76E-149	Treg	Rbm38
2.96E-153	0.25499351	0.884	0.84	4.75E-149	Treg	Gng5
8.70E-151	0.324396477	0.718	0.612	1.39E-146	Treg	Map1lc3b
1.24E-150	0.365466181	0.345	0.195	1.98E-146	Treg	Vcpkmt
8.89E-150	0.370659635	0.551	0.399	1.42E-145	Treg	Plp2
2.02E-148	0.346908713	0.299	0.156	3.23E-144	Treg	Il12rb1
1.67E-147	0.343096466	0.67	0.546	2.67E-143	Treg	Bnip3l
5.15E-146	0.32416559	0.311	0.163	8.25E-142	Treg	Csrnp1
7.79E-145	0.312287569	0.913	0.877	1.25E-140	Treg	H2-Q7
1.16E-143	0.342065198	0.3	0.159	1.86E-139	Treg	Tank
1.20E-143	0.329229952	0.279	0.142	1.92E-139	Treg	Stx11
2.22E-143	0.342214985	0.408	0.257	3.56E-139	Treg	Map2k3
2.45E-142	0.338416971	0.602	0.465	3.93E-138	Treg	Ptp4a2
2.74E-141	0.301396086	0.777	0.688	4.39E-137	Treg	Cd53
2.91E-141	0.318697072	0.254	0.124	4.67E-137	Treg	Rhoc
6.94E-141	0.342223912	0.557	0.412	1.11E-136	Treg	Rps6ka1
9.33E-141	0.305760356	0.34	0.191	1.50E-136	Treg	Lxn
5.43E-140	0.350981218	0.566	0.413	8.70E-136	Treg	Gbp7
2.77E-139	0.290155471	0.739	0.641	4.43E-135	Treg	Selenok
3.29E-139	0.335289331	0.377	0.224	5.27E-135	Treg	Gata3
8.64E-139	0.311683863	0.287	0.149	1.38E-134	Treg	Nmb
1.28E-138	0.302344896	0.298	0.155	2.05E-134	Treg	Slamf1
1.61E-138	0.316425452	0.277	0.142	2.58E-134	Treg	Grn
1.40E-137	0.360701238	0.643	0.514	2.25E-133	Treg	Peli1
1.42E-137	0.329835062	0.526	0.37	2.28E-133	Treg	Gna13

4.27E-137	0.313877973	0.276	0.142	6.84E-133	Treg	Dok1
2.75E-136	0.317789773	0.746	0.651	4.41E-132	Treg	Sqstm1
1.34E-135	0.285469379	0.908	0.758	2.14E-131	Treg	S100a6
1.34E-134	0.307757158	0.771	0.682	2.15E-130	Treg	Ets1
3.92E-134	0.332163135	0.634	0.511	6.28E-130	Treg	Pycard
1.03E-133	0.304133711	0.723	0.612	1.65E-129	Treg	Tap2
6.54E-133	0.379610335	0.603	0.456	1.05E-128	Treg	Igtp
2.53E-132	0.332293144	0.511	0.367	4.06E-128	Treg	Ncf4
2.80E-132	0.288996313	0.746	0.647	4.49E-128	Treg	Aup1
1.96E-131	0.367565345	0.458	0.315	3.14E-127	Treg	Dnajc1
2.98E-130	0.29322837	0.252	0.125	4.77E-126	Treg	Hbs1l
3.60E-129	0.291602227	0.778	0.699	5.78E-125	Treg	Vasp
9.35E-129	0.301029298	0.848	0.793	1.50E-124	Treg	Gimap3
1.03E-124	0.301469247	0.285	0.154	1.65E-120	Treg	Ccs
1.16E-124	0.321539137	0.577	0.443	1.85E-120	Treg	Cyth4
1.27E-123	0.337376501	0.628	0.504	2.04E-119	Treg	Pld3
8.86E-121	0.32257482	0.599	0.477	1.42E-116	Treg	Mtdh
6.31E-120	0.317007589	0.305	0.174	1.01E-115	Treg	Entpd1
7.87E-120	0.312203189	0.569	0.441	1.26E-115	Treg	Anxa11
2.70E-119	0.26385688	0.679	0.57	4.33E-115	Treg	Gpx1
8.31E-119	0.344020064	0.393	0.251	1.33E-114	Treg	Nr3c1
5.94E-118	0.264065535	0.799	0.727	9.52E-114	Treg	Prkar1a
5.93E-117	0.293954125	0.719	0.606	9.50E-113	Treg	Trac
2.07E-116	0.320503795	0.278	0.153	3.32E-112	Treg	P4ha1
4.21E-116	0.329024981	0.458	0.322	6.75E-112	Treg	Cast
7.23E-116	0.296415364	0.251	0.131	1.16E-111	Treg	Birc3
6.09E-113	0.265001571	0.802	0.738	9.77E-109	Treg	Anp32a
1.24E-111	0.267787113	0.866	0.834	1.98E-107	Treg	Gimap4
6.67E-109	0.297293444	0.622	0.512	1.07E-104	Treg	Fermt3
6.74E-109	0.313358505	0.276	0.156	1.08E-104	Treg	Syt11
1.90E-108	0.346190722	0.541	0.423	3.04E-104	Treg	Gstp3
4.87E-108	0.315997863	0.523	0.397	7.81E-104	Treg	Serp1
7.85E-108	0.286858236	0.652	0.541	1.26E-103	Treg	Fmnl1
5.09E-107	0.305484996	0.593	0.478	8.16E-103	Treg	Ctsa
5.58E-107	0.290133829	0.271	0.151	8.95E-103	Treg	Rara
1.86E-104	0.294734429	0.639	0.52	2.98E-100	Treg	Ccnd2
1.00E-103	0.2817557	0.742	0.649	1.61E-99	Treg	Arf6
2.41E-103	0.332441599	0.38	0.255	3.86E-99	Treg	Ciapin1
2.82E-103	0.317166516	0.565	0.447	4.52E-99	Treg	Cst3

3.06E-103	0.298736707	0.464	0.336	4.90E-99	Treg	Pold4
5.85E-103	0.291350415	0.644	0.529	9.37E-99	Treg	Tpm4
2.89E-102	0.292397555	0.432	0.309	4.63E-98	Treg	BC004004
1.94E-101	0.275646092	0.601	0.486	3.11E-97	Treg	Chmp2a
1.93E-100	0.320176285	0.418	0.293	3.09E-96	Treg	Ehd1
2.55E-100	0.328724122	0.684	0.614	4.08E-96	Treg	Mdh1
6.72E-100	0.304739205	0.581	0.467	1.08E-95	Treg	Tgfb1
7.20E-99	0.38075978	0.631	0.534	1.15E-94	Treg	Ubald2
1.26E-98	0.283324811	0.623	0.526	2.02E-94	Treg	Csk
1.52E-98	0.273379947	0.638	0.533	2.44E-94	Treg	Lcp2
2.12E-98	0.308195055	0.455	0.329	3.40E-94	Treg	Tmem173
3.21E-98	0.311253815	0.365	0.242	5.15E-94	Treg	Ap3b1
4.90E-97	0.28755494	0.569	0.454	7.85E-93	Treg	Smpdl3a
1.53E-96	0.303156506	0.436	0.31	2.46E-92	Treg	S1pr4
2.48E-95	0.315336714	0.392	0.269	3.98E-91	Treg	Pfkl
4.07E-95	0.309924344	0.377	0.251	6.52E-91	Treg	Orai1
4.59E-95	0.260010891	0.689	0.598	7.36E-91	Treg	H13
5.85E-95	0.281720313	0.411	0.287	9.37E-91	Treg	Fnbp1
7.66E-95	0.28742938	0.337	0.218	1.23E-90	Treg	Mthfsl
3.53E-94	0.306273191	0.427	0.305	5.66E-90	Treg	Rabgap1l
5.10E-94	0.275837388	0.575	0.461	8.17E-90	Treg	Aes
7.44E-94	0.276792992	0.585	0.48	1.19E-89	Treg	Ppdpf
1.08E-92	0.271126158	0.656	0.558	1.74E-88	Treg	Rbms1
1.26E-92	0.279291632	0.377	0.253	2.02E-88	Treg	Arhgap4
2.54E-92	0.256235836	0.69	0.605	4.08E-88	Treg	Pla2g16
3.01E-92	0.296069494	0.778	0.698	4.83E-88	Treg	Stat1
7.13E-92	0.277338581	0.748	0.686	1.14E-87	Treg	Eno1
3.51E-91	0.261137723	0.294	0.18	5.63E-87	Treg	Fbxo6
3.55E-91	0.401711435	0.517	0.402	5.69E-87	Treg	Serpina3g
4.31E-90	0.258046608	0.288	0.175	6.91E-86	Treg	Phyh
3.15E-89	0.276682677	0.366	0.247	5.05E-85	Treg	Capn2
3.04E-88	0.374064535	0.306	0.197	4.88E-84	Treg	Slc2a1
3.30E-88	0.262600694	0.642	0.547	5.30E-84	Treg	Ube2b
1.43E-86	0.292739069	0.27	0.165	2.29E-82	Treg	Fdft1
1.63E-86	0.254825919	0.413	0.289	2.61E-82	Treg	Rcsd1
2.50E-86	0.290051073	0.458	0.344	4.01E-82	Treg	Rab11a
4.23E-86	0.278665893	0.453	0.33	6.79E-82	Treg	Inpp4b
3.80E-85	0.257965049	0.341	0.224	6.09E-81	Treg	Cers4
2.88E-84	0.267757177	0.521	0.408	4.61E-80	Treg	Slc50a1

2.41E-83	0.273224893	0.58	0.484	3.86E-79	Treg	Lamtor4
3.70E-83	0.317923624	0.584	0.481	5.93E-79	Treg	Stat3
8.81E-82	0.338500936	0.37	0.26	1.41E-77	Treg	Ccr5
1.13E-81	0.261341851	0.272	0.167	1.82E-77	Treg	Srebf2
1.77E-81	0.2610546	0.512	0.389	2.84E-77	Treg	S100a4
3.57E-81	0.271783656	0.451	0.339	5.72E-77	Treg	Cdipt
8.09E-80	0.276765562	0.501	0.391	1.30E-75	Treg	Kdelr1
1.92E-79	0.310052423	0.457	0.35	3.07E-75	Treg	Batf
2.15E-76	0.28393303	0.393	0.288	3.45E-72	Treg	Vmp1
7.16E-76	0.272300496	0.394	0.281	1.15E-71	Treg	Cdkn1b
1.08E-75	0.25277604	0.335	0.225	1.73E-71	Treg	Tnip1
9.33E-75	0.250542377	0.68	0.598	1.49E-70	Treg	Ppp1r12a
6.74E-74	0.256555764	0.459	0.35	1.08E-69	Treg	AI467606
1.33E-73	0.268116005	0.7	0.624	2.13E-69	Treg	Pgam1
4.38E-71	0.265273374	0.274	0.177	7.02E-67	Treg	Pgm2
1.84E-62	0.267153055	0.479	0.378	2.95E-58	Treg	Tsc22d3
3.09E-60	0.288274508	0.427	0.334	4.95E-56	Treg	Hopx
2.44E-59	0.26211644	0.405	0.313	3.91E-55	Treg	Camk4
2.53E-59	0.327542814	0.348	0.255	4.06E-55	Treg	Icam1
4.00E-59	0.250611087	0.665	0.596	6.42E-55	Treg	H2-Q6
1.53E-36	0.303742685	0.292	0.223	2.46E-32	Treg	Irf8
0	0.938869125	0.334	0.037	0	Cd4	Cd40lg
0	0.660866741	0.455	0.204	0	Cd4	Cd4
2.06E-252	0.726875916	0.578	0.382	3.31E-248	Cd4	Itgb1
4.85E-136	0.655863372	0.125	0.317	7.77E-132	Cd4	Ccl4
7.75E-121	0.425489386	0.911	0.888	1.24E-116	Cd4	Junb
9.13E-111	0.37868225	0.75	0.727	1.46E-106	Cd4	Slfn2
2.40E-110	0.514369816	0.488	0.371	3.84E-106	Cd4	Btg2
5.58E-105	0.454296526	0.574	0.455	8.94E-101	Cd4	Emb
5.14E-75	0.403632611	0.271	0.17	8.24E-71	Cd4	S1pr1
3.15E-57	0.422136118	0.559	0.515	5.05E-53	Cd4	Slfn1
3.20E-57	0.328023563	0.516	0.45	5.14E-53	Cd4	Cd28
3.16E-56	0.345620632	0.967	0.972	5.07E-52	Cd4	Malat1
1.34E-54	0.35114243	0.286	0.202	2.15E-50	Cd4	Il18r1
8.30E-54	0.288623359	0.301	0.204	1.33E-49	Cd4	Tcf7
8.05E-45	0.354085908	0.303	0.229	1.29E-40	Cd4	Gpr183
4.27E-42	0.413260902	0.432	0.359	6.84E-38	Cd4	Il7r
2.25E-36	0.301108467	0.995	0.998	3.61E-32	Cd4	Gm42418
1.20E-35	0.34273906	0.6	0.588	1.92E-31	Cd4	Ier2

1.05E-34	0.309030498	0.353	0.276	1.68E-30	Cd4	Klf2
1.39E-33	0.450694791	0.48	0.429	2.23E-29	Cd4	Fos
2.13E-31	0.323996394	0.433	0.387	3.41E-27	Cd4	Ier5
1.22E-29	0.258026543	0.279	0.219	1.96E-25	Cd4	Tespa1
4.22E-29	0.288609349	0.299	0.244	6.77E-25	Cd4	Gpr132
1.05E-23	0.280920437	0.267	0.216	1.69E-19	Cd4	Gm26740
2.68E-20	0.354871772	0.588	0.598	4.29E-16	Cd4	Zfp36l2
1.34E-18	0.37260609	0.266	0.223	2.15E-14	Cd4	Socs3
2.55E-18	0.303997028	0.308	0.277	4.08E-14	Cd4	Arid5a
2.22E-16	0.252561882	0.391	0.37	3.56E-12	Cd4	Rexo2
9.61E-16	0.283289501	0.323	0.297	1.54E-11	Cd4	Tgfbr2
1.52E-15	0.355884018	0.357	0.329	2.43E-11	Cd4	Tnfaip3
6.20E-15	0.283338078	0.44	0.425	9.94E-11	Cd4	Bhlhe40
2.63E-14	0.274913298	0.279	0.247	4.21E-10	Cd4	Itga4
2.50E-08	0.277130841	0.748	0.785	0.00040124	Cd4	Lars2
4.28E-06	0.25789865	0.547	0.576	0.068626275	Cd4	Klf6
4.34E-06	0.408152589	0.486	0.505	0.069498257	Cd4	AY036118
0.000512492	0.812815273	0.421	0.487	1	Cd4	Gm26917
0.000667407	0.282066922	0.265	0.257	1	Cd4	Ifit3
0.005254458	0.252582367	0.275	0.273	1	Cd4	Ifit1
0	1.021282745	0.642	0.114	0	Cd8 Naive	Sell
0	0.981714755	0.734	0.281	0	Cd8 Naive	Bcl2
0	0.891765631	0.445	0.073	0	Cd8 Naive	Ccr7
0	0.878577377	0.65	0.168	0	Cd8 Naive	Tcf7
0	0.863824638	0.679	0.241	0	Cd8 Naive	Klf2
0	0.80467316	0.613	0.242	0	Cd8 Naive	Cd7
0	0.78922348	0.538	0.136	0	Cd8 Naive	Satb1
0	0.747705117	0.471	0.157	0	Cd8 Naive	Pim2
0	0.734702095	0.993	0.913	0	Cd8 Naive	Rpl36a
0	0.72029824	0.254	0.038	0	Cd8 Naive	Dapl1
0	0.708213439	1	0.99	0	Cd8 Naive	Rps20
0	0.69373513	0.521	0.178	0	Cd8 Naive	Lef1
0	0.684405108	0.999	0.992	0	Cd8 Naive	Rplp1
0	0.670976345	0.373	0.053	0	Cd8 Naive	Nsg2
0	0.668774696	1	0.995	0	Cd8 Naive	Rps24
0	0.655327044	0.949	0.763	0	Cd8 Naive	mt-Nd2
0	0.652834981	0.995	0.946	0	Cd8 Naive	Rps28
0	0.652344251	1	0.987	0	Cd8 Naive	Rps19
0	0.648039254	0.995	0.958	0	Cd8 Naive	Rps29

0	0.638641031	0.993	0.954	0	Cd8 Naive	Rpl12
0	0.622497285	1	0.997	0	Cd8 Naive	Rpl13
0	0.618949512	0.998	0.972	0	Cd8 Naive	Rps7
0	0.604042623	1	0.987	0	Cd8 Naive	Rpl8
0	0.602860422	0.451	0.152	0	Cd8 Naive	Txk
0	0.594625963	1	0.996	0	Cd8 Naive	Rps16
0	0.588289239	1	0.979	0	Cd8 Naive	Rps21
0	0.588272914	0.999	0.966	0	Cd8 Naive	Rps18
0	0.58816456	0.998	0.982	0	Cd8 Naive	Rps27
0	0.581688151	0.982	0.889	0	Cd8 Naive	mt-Nd4l
0	0.57476901	1	0.993	0	Cd8 Naive	Rps5
0	0.570223503	0.974	0.859	0	Cd8 Naive	mt-Nd1
0	0.56892993	0.871	0.639	0	Cd8 Naive	mt-Nd5
0	0.562374274	1	0.986	0	Cd8 Naive	Rpl35a
0	0.559406317	1	0.993	0	Cd8 Naive	Rpl23
0	0.556610324	1	0.992	0	Cd8 Naive	Rps4x
0	0.555415647	1	0.993	0	Cd8 Naive	Rps8
0	0.551376446	0.986	0.908	0	Cd8 Naive	Rpl5
0	0.550568708	1	0.991	0	Cd8 Naive	Rpl21
0	0.549767789	1	0.991	0	Cd8 Naive	Rps27a
0	0.549328766	0.99	0.932	0	Cd8 Naive	Eef1b2
0	0.539473373	1	0.991	0	Cd8 Naive	Rps3a1
0	0.534058488	0.997	0.95	0	Cd8 Naive	Rpl35
0	0.512736533	0.997	0.972	0	Cd8 Naive	Rpl10a
0	0.504174741	0.996	0.954	0	Cd8 Naive	Rpl10
0	0.494244451	0.29	0.067	0	Cd8 Naive	Sh3bp5
0	0.493862317	1	0.992	0	Cd8 Naive	Rps10
0	0.493800229	0.998	0.975	0	Cd8 Naive	Rpl15
0	0.486994327	1	0.987	0	Cd8 Naive	Rps9
0	0.485409919	1	0.993	0	Cd8 Naive	Rpl9
0	0.482284718	0.999	0.983	0	Cd8 Naive	Rps23
0	0.479543748	0.993	0.95	0	Cd8 Naive	Rps6
0	0.475746134	0.97	0.87	0	Cd8 Naive	Npm1
0	0.473521574	0.999	0.979	0	Cd8 Naive	Rpl3
0	0.469334354	1	0.987	0	Cd8 Naive	mt-Atp6
0	0.468704311	1	0.992	0	Cd8 Naive	Rpl30
0	0.465381239	1	0.993	0	Cd8 Naive	Rpl32
0	0.464267893	0.995	0.975	0	Cd8 Naive	Rps12
0	0.461020804	1	0.992	0	Cd8 Naive	Rps3

0	0.460856953	0.991	0.942	0	Cd8 Naive	Rack1
0	0.45801518	0.999	0.981	0	Cd8 Naive	Rplp2
0	0.457797746	1	0.989	0	Cd8 Naive	Rps2
0	0.457787741	0.999	0.987	0	Cd8 Naive	Rpl36
0	0.452791656	1	0.993	0	Cd8 Naive	Rpl27a
0	0.451739276	0.996	0.969	0	Cd8 Naive	Rpl14
0	0.449618282	0.997	0.978	0	Cd8 Naive	Rpl26
0	0.449580177	1	0.987	0	Cd8 Naive	Rpl39
0	0.442194947	1	0.995	0	Cd8 Naive	Rpl18a
0	0.437236444	1	0.991	0	Cd8 Naive	Rpl37a
0	0.430330484	1	0.991	0	Cd8 Naive	Rpl37
0	0.420170714	1	0.986	0	Cd8 Naive	Rps26
0	0.419685876	0.998	0.976	0	Cd8 Naive	mt-Co3
0	0.418564168	1	0.993	0	Cd8 Naive	Rpl17
0	0.4184494	1	0.988	0	Cd8 Naive	Rpl6
0	0.417219056	0.999	0.98	0	Cd8 Naive	Rpl28
0	0.416702369	0.984	0.927	0	Cd8 Naive	Rpl4
0	0.415164363	1	0.994	0	Cd8 Naive	Rpl18
0	0.41086967	1	0.997	0	Cd8 Naive	Rpsa
0	0.406940835	0.996	0.97	0	Cd8 Naive	Rpl7
0	0.400616383	1	0.988	0	Cd8 Naive	Rps14
0	0.389049154	1	0.994	0	Cd8 Naive	Rplp0
0	0.386957304	1	0.996	0	Cd8 Naive	Rpl19
0	0.378554216	1	0.993	0	Cd8 Naive	Rps13
0	0.373310573	1	0.993	0	Cd8 Naive	Rps11
0	0.372365998	0.997	0.974	0	Cd8 Naive	Rpl38
0	0.359558755	1	0.995	0	Cd8 Naive	Rps15a
0	0.358250925	0.996	0.973	0	Cd8 Naive	Rpl22
0	0.356801383	0.999	0.994	0	Cd8 Naive	mt-Co1
0	0.345690602	1	0.992	0	Cd8 Naive	Rpl11
0	0.345421497	1	0.992	0	Cd8 Naive	Rpl34
0	0.338612626	1	0.995	0	Cd8 Naive	Tpt1
0	0.33836013	0.997	0.986	0	Cd8 Naive	mt-Co2
0	0.334990497	1	0.997	0	Cd8 Naive	Eef1a1
0	0.329954092	0.999	0.984	0	Cd8 Naive	Rpl29
0	0.275972702	1	0.995	0	Cd8 Naive	Rpl41
8.80E-307	0.408500586	0.993	0.935	1.41E-302	Cd8 Naive	mt-Cytb
4.02E-276	0.542352695	0.497	0.197	6.44E-272	Cd8 Naive	Socs3
2.50E-273	0.509865962	0.318	0.088	4.01E-269	Cd8 Naive	Klf3

4.21E-261	0.545441095	0.622	0.318	6.75E-257	Cd8 Naive	Evl
1.55E-252	0.538738672	0.5	0.221	2.48E-248	Cd8 Naive	Foxp1
6.28E-244	0.390453691	0.954	0.862	1.01E-239	Cd8 Naive	Rpl23a
9.78E-242	0.470923054	0.87	0.686	1.57E-237	Cd8 Naive	mt-Nd4
1.83E-241	0.494550341	0.324	0.105	2.93E-237	Cd8 Naive	Actn1
7.74E-236	0.449012718	0.806	0.447	1.24E-231	Cd8 Naive	Ly6c2
1.30E-229	0.477546198	0.466	0.2	2.08E-225	Cd8 Naive	Hmgn1
2.42E-229	0.399851082	0.939	0.836	3.88E-225	Cd8 Naive	Rps25
6.07E-228	0.472291087	0.752	0.444	9.72E-224	Cd8 Naive	Emb
8.51E-227	0.424113532	0.935	0.829	1.36E-222	Cd8 Naive	Rpl22l1
1.45E-223	0.501353537	0.339	0.118	2.32E-219	Cd8 Naive	Sidt1
8.59E-218	0.370825365	0.959	0.88	1.38E-213	Cd8 Naive	Rps15
1.69E-214	0.415573317	0.349	0.124	2.71E-210	Cd8 Naive	Gm2682
8.90E-201	0.432952496	0.389	0.158	1.43E-196	Cd8 Naive	Pdlim1
1.49E-187	0.412501544	0.841	0.698	2.39E-183	Cd8 Naive	Eef1g
6.23E-175	0.52364484	0.338	0.141	9.99E-171	Cd8 Naive	Klhdc2
2.03E-169	0.347144071	0.655	0.353	3.26E-165	Cd8 Naive	Klrd1
4.01E-167	0.430794802	0.483	0.25	6.42E-163	Cd8 Naive	Arl4c
5.04E-165	0.424567229	0.45	0.221	8.08E-161	Cd8 Naive	Gramd3
1.43E-147	0.370996975	0.391	0.181	2.29E-143	Cd8 Naive	Ssh2
8.53E-147	0.401982962	0.682	0.486	1.37E-142	Cd8 Naive	Nop10
1.04E-140	0.346542747	0.853	0.689	1.66E-136	Cd8 Naive	mt-Atp8
5.70E-136	0.412571339	0.547	0.338	9.14E-132	Cd8 Naive	Acp5
1.21E-135	0.427316233	0.321	0.143	1.94E-131	Cd8 Naive	Myc
1.04E-129	0.310009325	0.888	0.788	1.67E-125	Cd8 Naive	Rpl31
7.75E-128	0.348188597	0.312	0.138	1.24E-123	Cd8 Naive	Il4ra
4.00E-122	0.350117059	0.672	0.476	6.41E-118	Cd8 Naive	Dnajc15
1.37E-120	0.333109408	0.356	0.169	2.20E-116	Cd8 Naive	S1pr1
3.18E-111	0.365147777	0.493	0.306	5.10E-107	Cd8 Naive	Rps27rt
9.88E-111	0.285689206	0.752	0.592	1.58E-106	Cd8 Naive	Hspe1
1.63E-110	0.391587074	0.592	0.407	2.61E-106	Cd8 Naive	Ablim1
3.19E-110	0.379469568	0.517	0.34	5.11E-106	Cd8 Naive	Gm10073
4.48E-110	0.38748294	0.52	0.337	7.18E-106	Cd8 Naive	mt-Nd3
1.39E-107	0.34540539	0.487	0.3	2.22E-103	Cd8 Naive	Fbl
1.73E-107	0.353776182	0.493	0.312	2.77E-103	Cd8 Naive	Mrpl23
1.68E-105	0.339622184	0.324	0.163	2.70E-101	Cd8 Naive	Snhg12
7.37E-103	0.284815765	0.868	0.759	1.18E-98	Cd8 Naive	Rps17
2.64E-102	0.428225518	0.426	0.247	4.23E-98	Cd8 Naive	Jun
9.92E-102	0.356765641	0.5	0.319	1.59E-97	Cd8 Naive	Fam189b

1.24E-101	0.35378324	0.505	0.33	1.99E-97	Cd8 Naive	Gm9493
3.13E-99	0.26965513	0.364	0.187	5.02E-95	Cd8 Naive	Ms4a4c
9.53E-99	0.313103168	0.313	0.158	1.53E-94	Cd8 Naive	Epb41l4aos
2.13E-96	0.335598443	0.437	0.268	3.42E-92	Cd8 Naive	Impdh2
8.56E-95	0.289957885	0.308	0.153	1.37E-90	Cd8 Naive	Ripor2
6.32E-88	0.269514225	0.554	0.35	1.01E-83	Cd8 Naive	Il7r
6.37E-87	0.31330931	0.391	0.23	1.02E-82	Cd8 Naive	Atp11b
1.32E-86	0.274322183	0.318	0.167	2.12E-82	Cd8 Naive	Bzw2
1.86E-85	0.386990114	0.567	0.404	2.99E-81	Cd8 Naive	Vps37b
1.93E-85	0.309944026	0.35	0.196	3.09E-81	Cd8 Naive	Klhl6
4.65E-84	0.273562199	0.294	0.15	7.45E-80	Cd8 Naive	Gpr18
1.03E-81	0.299217632	0.653	0.516	1.65E-77	Cd8 Naive	Gm11808
3.00E-81	0.32916365	0.513	0.358	4.80E-77	Cd8 Naive	Klk8
7.99E-79	0.341019013	0.708	0.575	1.28E-74	Cd8 Naive	Ier2
1.24E-76	0.251274127	0.794	0.673	1.99E-72	Cd8 Naive	Trmt112
1.61E-74	0.285036261	0.453	0.299	2.58E-70	Cd8 Naive	C1qbp
1.05E-69	0.282285482	0.592	0.445	1.68E-65	Cd8 Naive	Rapgef6
5.62E-69	0.284242248	0.355	0.218	9.01E-65	Cd8 Naive	AB124611
5.14E-67	0.309831627	0.575	0.422	8.24E-63	Cd8 Naive	Fos
1.13E-60	0.280658854	0.375	0.244	1.81E-56	Cd8 Naive	Pik3cd
2.30E-55	0.252972129	0.521	0.392	3.68E-51	Cd8 Naive	Npm3
3.08E-53	0.252713463	0.374	0.251	4.93E-49	Cd8 Naive	H2-Ke6
0	3.513173327	0.938	0.003	0	Tgd	Trdv4
0	3.394776238	0.629	0.003	0	Tgd	Il17a
0	2.463409127	0.888	0.006	0	Tgd	Cd163l1
0	2.29014726	0.902	0.039	0	Tgd	Tmem176b
0	2.118564265	0.83	0.003	0	Tgd	Tcrg-V6
0	2.111512486	0.88	0.076	0	Tgd	Ckb
0	2.041663035	0.854	0.029	0	Tgd	Tmem176a
0	1.764573771	0.83	0.048	0	Tgd	Ltb4r1
0	1.706521566	0.775	0.011	0	Tgd	Tcrg-C1
0	1.636529911	0.741	0.017	0	Tgd	Trdc
0	1.575909285	0.698	0.001	0	Tgd	Blk
0	1.563356592	0.791	0.068	0	Tgd	Actn2
0	1.44642845	0.67	0.055	0	Tgd	Serpinb1a
0	1.425752175	0.727	0.078	0	Tgd	Smox
0	1.425137034	0.795	0.195	0	Tgd	Selenop
0	1.412223529	0.859	0.207	0	Tgd	Ramp1
0	1.239568346	0.586	0.008	0	Tgd	Rorc

0	1.17256074	0.571	0.003	0	Tgd	Ppp1r14c
0	1.166333771	0.568	0.056	0	Tgd	Furin
0	1.152404486	0.536	0.039	0	Tgd	Avpi1
0	1.09112494	0.539	0.028	0	Tgd	Aqp3
0	1.076921443	0.548	0.04	0	Tgd	Ly6g5b
0	0.936882897	0.461	0.027	0	Tgd	Tcrg-C4
0	0.911300607	0.441	0.003	0	Tgd	Zbtb16
0	0.806051873	0.348	0	0	Tgd	Abi3bp
0	0.786547079	0.382	0.015	0	Tgd	Slc6a13
0	0.785531597	0.459	0.048	0	Tgd	Tnfrsf25
0	0.677615103	0.318	0.001	0	Tgd	Prr29
0	0.635114276	0.311	0.007	0	Tgd	Lrrc25
0	0.632047642	0.305	0.005	0	Tgd	Irak3
0	0.610495914	0.305	0	0	Tgd	Sox13
0	0.594598627	0.298	0.005	0	Tgd	1700012B07Rik
0	0.583747051	0.279	0.002	0	Tgd	Il1r1
0	0.571695266	0.262	0.001	0	Tgd	Il23r
0	0.55657366	0.282	0.021	0	Tgd	Rasa4
0	0.546820934	0.259	0.001	0	Tgd	Bcl2a1a
0	0.546299637	0.259	0.005	0	Tgd	Il17re
0	0.531324752	0.275	0.004	0	Tgd	Clnk
4.37E-258	1.060334997	0.491	0.082	7.01E-254	Tgd	Naga
1.97E-254	1.328816441	0.986	0.518	3.15E-250	Tgd	Cxcr6
5.75E-254	0.875329025	0.559	0.107	9.22E-250	Tgd	Prelid2
2.18E-229	0.909401981	0.577	0.127	3.50E-225	Tgd	F2r
2.74E-217	0.552243051	0.257	0.025	4.38E-213	Tgd	Lmo4
8.89E-192	1.219620676	0.946	0.589	1.42E-187	Tgd	Gpx1
2.50E-187	0.794891531	0.998	0.961	4.01E-183	Tgd	Itm2b
1.67E-177	0.754269758	0.482	0.108	2.68E-173	Tgd	Plin2
1.00E-165	0.679825151	0.455	0.1	1.61E-161	Tgd	Podnl1
9.27E-157	0.606722617	0.346	0.062	1.49E-152	Tgd	Prrt1
2.81E-150	0.799248098	0.645	0.197	4.51E-146	Tgd	Maf
8.38E-150	0.838956264	0.461	0.11	1.34E-145	Tgd	Tnf
1.97E-146	0.800515989	0.988	0.611	3.15E-142	Tgd	Capg
1.65E-138	0.709277754	0.366	0.072	2.65E-134	Tgd	Rln3
2.73E-134	0.787641919	0.9	0.47	4.38E-130	Tgd	Emb
2.05E-129	0.817339787	0.921	0.613	3.29E-125	Tgd	Sec11c
5.85E-129	0.824266829	0.764	0.365	9.37E-125	Tgd	Rexo2
9.67E-127	0.521924373	0.28	0.049	1.55E-122	Tgd	Slc15a3

1.74E-124	0.669284758	0.393	0.095	2.79E-120	Tgd	Serpina3f
1.61E-121	0.563560856	0.382	0.091	2.58E-117	Tgd	Adgrg5
3.09E-121	0.48761438	0.3	0.058	4.95E-117	Tgd	Myo1e
1.26E-112	0.657256297	0.439	0.124	2.02E-108	Tgd	Znrf1
1.90E-112	0.675429786	0.97	0.809	3.04E-108	Tgd	Tagln2
1.22E-111	0.419829256	0.286	0.056	1.95E-107	Tgd	Selenom
2.41E-110	0.562554843	0.384	0.099	3.86E-106	Tgd	Pik3ap1
2.86E-108	0.840451866	0.902	0.631	4.58E-104	Tgd	Hsp90b1
1.67E-105	0.824012011	0.675	0.278	2.68E-101	Tgd	Dgat1
3.47E-102	0.78937526	0.798	0.468	5.57E-98	Tgd	Manf
1.08E-101	0.648593088	0.673	0.272	1.73E-97	Tgd	Rora
4.44E-101	0.618213404	0.527	0.184	7.12E-97	Tgd	Il12rb1
2.39E-100	0.664661122	0.995	0.859	3.83E-96	Tgd	Ly6a
2.05E-99	0.710572443	0.479	0.157	3.28E-95	Tgd	Ero1l
2.51E-98	0.658702408	0.484	0.163	4.03E-94	Tgd	Adam8
2.05E-97	0.686209712	0.746	0.393	3.28E-93	Tgd	Surf4
3.11E-94	0.551482149	0.971	0.858	4.99E-90	Tgd	Tspo
6.48E-93	0.578482532	0.605	0.235	1.04E-88	Tgd	Gpr183
6.65E-89	0.632808113	0.666	0.29	1.07E-84	Tgd	Jaml
1.01E-88	0.347451736	0.998	0.987	1.62E-84	Tgd	B2m
2.79E-88	0.523491312	0.305	0.076	4.47E-84	Tgd	AC163354.1
7.12E-88	0.748630054	0.807	0.511	1.14E-83	Tgd	Lgals3bp
6.06E-87	0.658949149	0.829	0.55	9.71E-83	Tgd	Aprt
6.34E-87	0.453346274	0.289	0.068	1.02E-82	Tgd	Tmem64
5.10E-86	0.555992309	0.798	0.399	8.18E-82	Tgd	Ccr2
6.34E-86	0.69120079	0.761	0.431	1.02E-81	Tgd	Dap
2.26E-85	0.680438716	0.516	0.185	3.62E-81	Tgd	Trgv2
1.14E-81	0.447650904	0.27	0.065	1.83E-77	Tgd	Scpep1
4.60E-81	0.55747425	0.986	0.889	7.37E-77	Tgd	Lgals1
8.74E-76	0.605306281	0.996	0.945	1.40E-71	Tgd	Srgn
7.21E-74	0.369554985	0.996	0.972	1.16E-69	Tgd	Ly6e
1.03E-73	0.565014342	0.591	0.274	1.65E-69	Tgd	Cd44
1.25E-73	0.472030919	0.95	0.794	2.00E-69	Tgd	Cox5a
4.90E-73	0.484970249	0.984	0.838	7.86E-69	Tgd	S100a11
3.05E-70	0.537828015	0.636	0.317	4.89E-66	Tgd	Ap3s1
7.77E-70	0.537140338	0.977	0.893	1.25E-65	Tgd	Ltb
8.86E-70	0.568336265	0.818	0.477	1.42E-65	Tgd	Icos
1.28E-69	0.551940812	0.623	0.29	2.06E-65	Tgd	Fgl2
1.27E-67	0.473209127	0.405	0.147	2.04E-63	Tgd	Ppt2

5.26E-67	0.535991385	0.486	0.208	8.43E-63	Tgd	Comt
3.10E-66	0.311249462	0.996	0.971	4.97E-62	Tgd	Oaz1
4.20E-66	0.485896664	0.92	0.765	6.73E-62	Tgd	Sec61g
4.50E-66	0.623919987	0.848	0.62	7.21E-62	Tgd	Zap70
1.04E-65	0.569287419	0.616	0.312	1.67E-61	Tgd	Snx2
6.04E-65	0.467797297	0.988	0.791	9.68E-61	Tgd	S100a6
1.55E-64	0.468675649	0.943	0.824	2.48E-60	Tgd	Sec61b
8.94E-64	0.753532167	0.673	0.403	1.43E-59	Tgd	Rilpl2
1.00E-63	0.674083596	0.611	0.322	1.61E-59	Tgd	Pdia6
1.50E-63	0.490498906	0.87	0.638	2.40E-59	Tgd	Spcs2
2.78E-63	0.639811504	0.562	0.26	4.46E-59	Tgd	Jun
3.64E-62	0.482342309	0.954	0.852	5.83E-58	Tgd	Lat
2.59E-61	0.479897372	0.989	0.926	4.15E-57	Tgd	Aldoa
3.12E-61	0.467832195	0.43	0.172	5.00E-57	Tgd	Riox2
3.98E-61	0.511597485	0.348	0.118	6.38E-57	Tgd	Rgcc
1.73E-60	0.500752985	0.964	0.883	2.78E-56	Tgd	Thy1
3.16E-60	0.462892642	0.979	0.894	5.06E-56	Tgd	Pkm
5.62E-60	0.425339381	0.979	0.93	9.01E-56	Tgd	S100a10
6.78E-60	0.649407536	0.845	0.639	1.09E-55	Tgd	Pgam1
1.75E-59	0.63964545	0.479	0.218	2.81E-55	Tgd	Sdf2l1
3.51E-59	0.510948075	0.889	0.672	5.62E-55	Tgd	Cd82
8.71E-58	0.563902016	0.52	0.24	1.40E-53	Tgd	Ube2l6
1.22E-57	0.71911283	0.739	0.488	1.96E-53	Tgd	Igtp
4.63E-57	0.498245063	0.462	0.204	7.42E-53	Tgd	Acat1
1.46E-55	0.495343749	0.521	0.254	2.34E-51	Tgd	Akirin1
2.47E-55	0.444018406	0.339	0.122	3.95E-51	Tgd	Crmp1
2.59E-55	0.464749581	0.945	0.856	4.15E-51	Tgd	Psme2
4.18E-55	0.450108679	0.371	0.143	6.69E-51	Tgd	Bcl2a1d
4.98E-55	0.439875616	0.489	0.207	7.99E-51	Tgd	Il2ra
1.27E-54	0.556696994	0.741	0.492	2.03E-50	Tgd	P4hb
3.70E-54	0.306291273	0.989	0.956	5.94E-50	Tgd	Serf2
2.39E-53	0.492401191	0.764	0.518	3.83E-49	Tgd	Stk24
5.97E-53	0.360435895	0.289	0.095	9.57E-49	Tgd	St6galnac3
8.83E-53	0.369051824	0.3	0.101	1.41E-48	Tgd	Plin3
1.22E-52	0.47819128	0.386	0.157	1.95E-48	Tgd	Rbpj
2.30E-52	0.462875211	0.877	0.694	3.69E-48	Tgd	Cdk2ap2
2.00E-50	0.611103868	0.848	0.649	3.21E-46	Tgd	Hspa5
5.79E-50	0.542638356	0.659	0.399	9.28E-46	Tgd	Pfkp
6.62E-50	0.449564428	0.855	0.67	1.06E-45	Tgd	Pomp

2.15E-49	0.432716105	0.843	0.631	3.45E-45	Tgd	Prdx6
7.35E-49	0.667544369	0.364	0.148	1.18E-44	Tgd	Iigp1
8.35E-49	0.555405286	0.809	0.606	1.34E-44	Tgd	Bsg
3.57E-48	0.443682561	0.882	0.749	5.72E-44	Tgd	Krtcap2
4.26E-47	0.497087109	0.586	0.307	6.83E-43	Tgd	Serpinb6a
1.08E-46	0.440212726	0.905	0.768	1.73E-42	Tgd	Pdia3
2.64E-46	0.465381324	0.841	0.667	4.24E-42	Tgd	Ssr4
3.38E-46	0.350588215	0.304	0.112	5.42E-42	Tgd	Ppp1r14b
5.00E-46	0.350983972	0.27	0.092	8.01E-42	Tgd	Agpat5
5.14E-46	0.527893281	0.689	0.424	8.25E-42	Tgd	Ikzf3
6.83E-46	0.396921114	0.327	0.124	1.10E-41	Tgd	Rarg
1.35E-45	0.392499994	0.468	0.224	2.17E-41	Tgd	Fkbp2
1.81E-45	0.368225161	0.298	0.106	2.90E-41	Tgd	Oasl2
2.35E-45	0.421553753	0.979	0.93	3.76E-41	Tgd	Ldha
7.74E-45	0.359073404	0.329	0.128	1.24E-40	Tgd	Parp1
8.02E-44	0.444007849	0.821	0.622	1.28E-39	Tgd	Cd247
8.71E-44	0.464561501	0.386	0.173	1.40E-39	Tgd	St3gal6
1.08E-43	0.395365426	0.382	0.167	1.73E-39	Tgd	Ccdc50
1.41E-41	0.364087256	0.888	0.72	2.25E-37	Tgd	Cox6a1
1.89E-41	0.49288214	0.786	0.577	3.03E-37	Tgd	Psap
1.97E-41	0.392101283	0.816	0.596	3.16E-37	Tgd	Smdt1
5.76E-41	0.38535975	0.395	0.176	9.24E-37	Tgd	Rara
1.58E-40	0.361141654	0.421	0.192	2.53E-36	Tgd	Tmem154
4.46E-40	0.558947252	0.804	0.631	7.15E-36	Tgd	Tpi1
1.14E-39	0.437480143	0.611	0.371	1.82E-35	Tgd	Itgal
1.34E-39	0.473363391	0.725	0.499	2.15E-35	Tgd	Calr
2.65E-39	0.442759331	0.459	0.239	4.24E-35	Tgd	Pon2
3.31E-39	0.404112641	0.845	0.676	5.30E-35	Tgd	Tmed2
5.47E-39	0.30590933	0.254	0.092	8.77E-35	Tgd	Mcur1
2.16E-38	0.33613665	0.327	0.139	3.46E-34	Tgd	11-Sep
2.59E-38	0.417330329	0.639	0.394	4.15E-34	Tgd	Cstb
2.91E-38	0.404401886	0.359	0.161	4.67E-34	Tgd	Eif4e3
3.05E-38	0.416679794	0.648	0.427	4.89E-34	Tgd	Ndufs5
3.15E-38	0.396022276	0.698	0.472	5.05E-34	Tgd	Cst3
3.44E-38	0.415657065	0.405	0.199	5.51E-34	Tgd	Bri3bp
1.35E-37	0.549453964	0.661	0.425	2.17E-33	Tgd	Serpina3g
7.27E-37	0.363246348	0.879	0.744	1.17E-32	Tgd	Prr13
3.19E-36	0.369253139	0.368	0.171	5.11E-32	Tgd	Plekho2
3.53E-36	0.447232768	0.604	0.382	5.66E-32	Tgd	Ppp1r11

1.01E-35	0.307711744	0.279	0.11	1.61E-31	Tgd	Dctn1
1.27E-35	0.455292023	0.491	0.276	2.04E-31	Tgd	Pdia4
4.94E-35	0.399086106	0.705	0.503	7.92E-31	Tgd	Capza2
5.36E-35	0.427094987	0.657	0.444	8.59E-31	Tgd	Cd164
1.08E-34	0.447726226	0.766	0.54	1.72E-30	Tgd	Rgs1
1.13E-34	0.438693538	0.846	0.662	1.81E-30	Tgd	Bst2
1.69E-34	0.367373406	0.768	0.545	2.71E-30	Tgd	Rwdd1
1.87E-34	0.794764166	0.712	0.528	3.00E-30	Tgd	Ndufa1
2.32E-34	0.304007545	0.948	0.839	3.72E-30	Tgd	Ybx1
2.87E-34	0.483416502	0.6	0.392	4.60E-30	Tgd	Ptpn6
5.88E-34	0.289124894	0.268	0.106	9.42E-30	Tgd	Uhrf1bp1l
1.11E-33	0.309035566	0.336	0.149	1.78E-29	Tgd	Runx1
1.14E-33	0.380366788	0.45	0.229	1.82E-29	Tgd	Slc16a3
1.29E-33	0.354780072	0.543	0.311	2.07E-29	Tgd	Trpv2
1.64E-33	0.391599477	0.495	0.277	2.63E-29	Tgd	Xbp1
1.83E-33	0.413557377	0.538	0.324	2.93E-29	Tgd	Selenos
1.87E-33	0.380012995	0.654	0.428	3.00E-29	Tgd	Rpn2
2.00E-33	0.331803544	0.879	0.71	3.21E-29	Tgd	Fkbp1a
2.01E-33	0.362278322	0.348	0.164	3.23E-29	Tgd	Osbpl3
2.51E-33	0.313648543	0.896	0.807	4.03E-29	Tgd	Cd47
2.52E-33	0.336598669	0.868	0.717	4.04E-29	Tgd	Ndufa4
1.52E-32	0.341696831	0.302	0.133	2.44E-28	Tgd	Clic4
1.36E-31	0.312298638	0.92	0.795	2.18E-27	Tgd	Gmfg
2.29E-31	0.348881772	0.496	0.288	3.67E-27	Tgd	Tmbim4
3.21E-31	0.367468205	0.523	0.31	5.14E-27	Tgd	Canx
6.55E-31	0.393118032	0.871	0.724	1.05E-26	Tgd	Pgk1
1.51E-30	0.350347385	0.389	0.202	2.43E-26	Tgd	Bcl2a1b
1.69E-30	0.425766476	0.795	0.561	2.71E-26	Tgd	Zfp36l1
1.80E-30	0.361667879	0.654	0.446	2.89E-26	Tgd	Tmed10
3.11E-30	0.320177971	0.379	0.189	4.98E-26	Tgd	Cmtm6
4.13E-30	0.34489794	0.441	0.238	6.62E-26	Tgd	Ppa1
5.96E-30	0.355639352	0.32	0.15	9.55E-26	Tgd	Eif4ebp1
1.00E-29	0.280580278	0.809	0.567	1.61E-25	Tgd	Itgb7
1.60E-29	0.335401876	0.393	0.208	2.57E-25	Tgd	Tfg
3.41E-29	0.284563585	0.945	0.858	5.46E-25	Tgd	Eif5a
3.47E-29	0.348965021	0.8	0.618	5.57E-25	Tgd	Cmtm7
6.45E-29	0.402141556	0.5	0.31	1.03E-24	Tgd	Rpn1
6.65E-29	0.336425175	0.388	0.205	1.07E-24	Tgd	Rftn1
7.83E-29	0.360106433	0.359	0.185	1.25E-24	Tgd	Agpat4

2.01E-28	0.336253195	0.482	0.275	3.22E-24	Tgd	Rasgrp1
2.34E-28	0.49076238	0.332	0.164	3.75E-24	Tgd	Egr1
2.38E-28	0.321926438	0.348	0.173	3.82E-24	Tgd	Stx11
6.19E-28	0.307446481	0.773	0.58	9.92E-24	Tgd	Selenof
9.31E-28	0.317460667	0.409	0.216	1.49E-23	Tgd	Cd226
1.17E-27	0.314334477	0.638	0.411	1.87E-23	Tgd	Cish
1.54E-27	0.316921696	0.891	0.701	2.47E-23	Tgd	Id2
3.53E-27	0.333791407	0.609	0.41	5.66E-23	Tgd	Rnh1
3.60E-27	0.322736317	0.702	0.513	5.77E-23	Tgd	Ostc
1.30E-26	0.401746756	0.73	0.551	2.09E-22	Tgd	Gstp1
1.52E-26	0.296871008	0.405	0.222	2.44E-22	Tgd	Mlec
2.25E-26	0.350509519	0.305	0.151	3.60E-22	Tgd	Creld2
3.18E-26	0.345774154	0.688	0.518	5.10E-22	Tgd	Ddost
4.00E-26	0.336743402	0.562	0.364	6.42E-22	Tgd	Diaph1
4.55E-26	0.276246291	0.932	0.846	7.30E-22	Tgd	Ppib
5.39E-26	0.280569602	0.325	0.158	8.63E-22	Tgd	Arhgap25
5.67E-26	0.511621419	0.355	0.187	9.10E-22	Tgd	Bnip3
1.07E-25	0.31260751	0.525	0.334	1.71E-21	Tgd	Sms
2.71E-25	0.25286215	0.275	0.128	4.34E-21	Tgd	Coro1c
4.39E-25	0.365488306	0.852	0.715	7.04E-21	Tgd	Stat1
4.73E-25	0.351705375	0.6	0.404	7.58E-21	Tgd	Sptssa
4.27E-24	0.292746974	0.42	0.241	6.84E-20	Tgd	Impa1
5.06E-24	0.332713711	0.412	0.245	8.12E-20	Tgd	Timm23
5.55E-24	0.29875526	0.707	0.504	8.89E-20	Tgd	Cd69
6.87E-24	0.32111654	0.434	0.251	1.10E-19	Tgd	Mapkapk3
6.95E-24	0.272626958	0.373	0.203	1.11E-19	Tgd	Dctpp1
7.33E-24	0.346356764	0.386	0.218	1.17E-19	Tgd	Clcn3
7.80E-24	0.299517247	0.3	0.149	1.25E-19	Tgd	Ranbp2
1.49E-23	0.250258733	0.471	0.274	2.39E-19	Tgd	Pde4b
2.61E-23	0.315570123	0.336	0.179	4.19E-19	Tgd	Gpr65
4.32E-23	0.276853659	0.43	0.252	6.92E-19	Tgd	Tor1aip1
5.72E-23	0.291735323	0.323	0.169	9.17E-19	Tgd	Tbl1x
6.58E-23	0.288962063	0.541	0.341	1.05E-18	Tgd	Psen2
8.88E-23	0.295809241	0.779	0.632	1.42E-18	Tgd	Spcs1
1.81E-22	0.307339406	0.673	0.496	2.90E-18	Tgd	H2-T23
2.12E-22	0.264066381	0.925	0.842	3.39E-18	Tgd	Cox7c
3.24E-22	0.500600699	0.346	0.191	5.19E-18	Tgd	Hilpda
3.61E-22	0.288542147	0.384	0.22	5.78E-18	Tgd	Szrd1
5.44E-22	0.281138653	0.659	0.466	8.72E-18	Tgd	Cyb5a

5.96E-22	0.328309634	0.311	0.164	9.56E-18	Tgd	Tab2
6.78E-22	0.277816939	0.727	0.564	1.09E-17	Tgd	Psmd8
8.21E-22	0.267896191	0.896	0.814	1.32E-17	Tgd	Ubl5
9.58E-22	0.323263126	0.445	0.28	1.54E-17	Tgd	Tmem208
2.63E-21	0.30409656	0.679	0.503	4.21E-17	Tgd	Tax1bp1
3.64E-21	0.312646136	0.839	0.699	5.83E-17	Tgd	Eno1
3.87E-21	0.261337199	0.875	0.751	6.20E-17	Tgd	Anp32a
7.16E-21	0.262360626	0.339	0.184	1.15E-16	Tgd	Nfil3
1.09E-20	0.29890342	0.446	0.273	1.74E-16	Tgd	Irgm1
1.17E-20	0.255173424	0.938	0.858	1.87E-16	Tgd	Selenow
1.89E-20	0.289719589	0.502	0.329	3.02E-16	Tgd	Cib1
1.94E-20	0.310690997	0.779	0.634	3.12E-16	Tgd	Psmb10
2.55E-20	0.323927144	0.414	0.247	4.09E-16	Tgd	Tgtp2
3.78E-20	0.280165738	0.673	0.518	6.07E-16	Tgd	Tmem14c
4.95E-20	0.310322459	0.904	0.827	7.93E-16	Tgd	Mif
8.34E-20	0.256237004	0.695	0.519	1.34E-15	Tgd	M6pr
1.09E-19	0.26531671	0.762	0.606	1.74E-15	Tgd	Tecr
1.12E-19	0.309091106	0.493	0.33	1.80E-15	Tgd	Gbp4
1.22E-19	0.355384839	0.459	0.301	1.95E-15	Tgd	Gbp2
1.52E-19	0.277099768	0.775	0.661	2.43E-15	Tgd	Ppp1cc
1.64E-19	0.333983273	0.311	0.168	2.62E-15	Tgd	Plk3
2.27E-19	0.28502669	0.516	0.353	3.63E-15	Tgd	Dnajb11
4.77E-19	0.283359616	0.814	0.696	7.64E-15	Tgd	Ostf1
5.82E-19	0.323851873	0.696	0.503	9.33E-15	Tgd	Pdcd1
9.26E-19	0.266792598	0.534	0.362	1.48E-14	Tgd	D16Ertd472e
9.44E-19	0.305191301	0.584	0.416	1.51E-14	Tgd	Dnajc3
9.52E-19	0.324647801	0.65	0.479	1.53E-14	Tgd	Smpdl3a
1.03E-18	0.279812107	0.568	0.4	1.65E-14	Tgd	Ncor1
1.15E-18	0.251897192	0.825	0.693	1.84E-14	Tgd	Eif4a1
1.53E-18	0.286944223	0.257	0.134	2.46E-14	Tgd	Zfp414
1.79E-18	0.258705246	0.839	0.751	2.86E-14	Tgd	Slc25a5
1.85E-18	0.277677117	0.48	0.314	2.96E-14	Tgd	Fnbp1
2.09E-18	0.403865109	0.514	0.361	3.36E-14	Tgd	Fam162a
6.09E-18	0.254857729	0.427	0.268	9.76E-14	Tgd	Utp11
8.88E-18	0.284703987	0.532	0.371	1.42E-13	Tgd	Spn
1.42E-17	0.25731104	0.348	0.203	2.28E-13	Tgd	Fam129a
2.66E-17	0.280305623	0.557	0.397	4.27E-13	Tgd	Ptpn7
5.16E-17	0.252590278	0.389	0.244	8.26E-13	Tgd	Tex264
5.41E-17	0.283197824	0.35	0.204	8.67E-13	Tgd	Phlda1

1.78E-16	0.266778916	0.486	0.331	2.85E-12	Tgd	Nfkbib
2.19E-16	0.253043981	0.388	0.244	3.51E-12	Tgd	Rrbp1
3.23E-16	0.272355878	0.254	0.137	5.18E-12	Tgd	Ap1s2
4.94E-16	0.275446247	0.502	0.351	7.91E-12	Tgd	Rtn4
1.93E-15	0.251590246	0.53	0.381	3.09E-11	Tgd	Cmpk1
2.54E-15	0.251154671	0.459	0.311	4.07E-11	Tgd	Top2b
3.07E-14	0.261438738	0.264	0.154	4.92E-10	Tgd	Gbp8
2.58E-13	0.281772436	0.452	0.322	4.13E-09	Tgd	Ehd1
3.62E-13	0.25303655	0.736	0.616	5.80E-09	Tgd	Ccnd3
8.13E-13	0.25242209	0.43	0.305	1.30E-08	Tgd	Ahsa1
9.94E-13	0.251803746	0.304	0.19	1.59E-08	Tgd	Rcbtb2
8.09E-10	0.254676059	0.68	0.559	1.30E-05	Tgd	Hsp90aa1
1.58E-06	0.368414723	0.296	0.228	0.02537182	Tgd	Gem
0	2.509741462	0.686	0.023	0	Cd4 Naive	Igfbp4
0	1.719501237	0.829	0.107	0	Cd4 Naive	Ccr7
0	1.096665927	0.323	0.016	0	Cd4 Naive	Ly6c1
7.08E-232	1.265703689	0.531	0.068	1.14E-227	Cd4 Naive	Rflnb
2.28E-207	1.742107302	0.929	0.283	3.65E-203	Cd4 Naive	Klf2
1.06E-182	1.331486892	0.783	0.21	1.70E-178	Cd4 Naive	Lef1
3.13E-181	1.002615672	0.444	0.06	5.01E-177	Cd4 Naive	Il6ra
2.94E-166	0.800414848	0.314	0.032	4.72E-162	Cd4 Naive	Atp1b1
1.04E-156	1.259784378	0.767	0.217	1.67E-152	Cd4 Naive	Tcf7
1.41E-146	0.673373255	1	0.996	2.27E-142	Cd4 Naive	Rps24
1.74E-136	0.7433002	1	0.989	2.79E-132	Cd4 Naive	Rps19
5.31E-134	0.670942308	1	0.988	8.51E-130	Cd4 Naive	Rpl8
8.97E-127	0.618207161	1	0.988	1.44E-122	Cd4 Naive	Rpl35a
1.46E-126	0.553959047	1	0.997	2.34E-122	Cd4 Naive	Rps16
7.79E-124	0.657170697	1	0.991	1.25E-119	Cd4 Naive	Rps20
5.89E-122	0.626939684	1	0.993	9.45E-118	Cd4 Naive	Rplp1
1.46E-121	0.559197341	1	0.996	2.35E-117	Cd4 Naive	Tpt1
2.26E-120	0.741915391	0.991	0.962	3.62E-116	Cd4 Naive	Rps29
1.15E-116	0.54087141	1	0.992	1.84E-112	Cd4 Naive	Rps27a
2.07E-114	0.575907017	1	0.994	3.31E-110	Cd4 Naive	Rps8
1.95E-112	0.55929273	1	0.993	3.13E-108	Cd4 Naive	Rps4x
3.85E-112	0.544855969	1	0.992	6.17E-108	Cd4 Naive	Rps3a1
3.86E-112	0.653528705	1	0.983	6.19E-108	Cd4 Naive	Rps27
5.70E-109	0.74450095	0.994	0.958	9.14E-105	Cd4 Naive	Rpl12
7.83E-105	1.043529945	0.599	0.178	1.26E-100	Cd4 Naive	Satb1
1.11E-102	0.482179349	0.997	0.994	1.78E-98	Cd4 Naive	Rpl23

1.23E-102	0.428021938	1	0.997	1.97E-98	Cd4 Naive	Rpl19
1.11E-101	0.467128319	1	0.993	1.78E-97	Cd4 Naive	Rpl30
6.23E-101	1.238899983	0.661	0.226	9.98E-97	Cd4 Naive	Socs3
1.02E-98	0.499867004	1	0.992	1.63E-94	Cd4 Naive	Rpl21
1.24E-98	0.463565893	1	0.997	2.00E-94	Cd4 Naive	Rpl13
5.24E-96	0.814997108	0.407	0.086	8.41E-92	Cd4 Naive	Nsg2
3.48E-94	0.578208686	1	0.975	5.57E-90	Cd4 Naive	Rps7
4.21E-94	0.540040535	1	0.981	6.75E-90	Cd4 Naive	Rps21
5.89E-94	0.918889885	0.481	0.126	9.43E-90	Cd4 Naive	Actn1
1.85E-89	0.45366026	0.997	0.989	2.96E-85	Cd4 Naive	Rps9
3.27E-89	0.556640855	1	0.988	5.25E-85	Cd4 Naive	mt-Atp6
6.50E-89	0.441485956	1	0.993	1.04E-84	Cd4 Naive	Rpl9
1.95E-88	0.449940761	1	0.993	3.13E-84	Cd4 Naive	Rps5
2.39E-87	0.940064931	0.447	0.111	3.83E-83	Cd4 Naive	Klf3
1.35E-86	0.611754516	0.997	0.951	2.17E-82	Cd4 Naive	Rps28
3.30E-86	0.419094919	1	0.993	5.29E-82	Cd4 Naive	Rps10
3.31E-86	0.445892086	1	0.989	5.31E-82	Cd4 Naive	Rps14
3.20E-85	0.697128208	0.273	0.045	5.13E-81	Cd4 Naive	Trib2
2.83E-83	0.906600713	0.553	0.171	4.54E-79	Cd4 Naive	Sell
2.59E-81	0.503648113	0.984	0.973	4.15E-77	Cd4 Naive	Rpl7
1.25E-80	0.641413799	0.981	0.916	2.00E-76	Cd4 Naive	Rpl5
1.64E-79	0.40177858	1	0.992	2.63E-75	Cd4 Naive	Rps3
4.20E-79	0.450772519	1	0.985	6.73E-75	Cd4 Naive	Rps23
2.78E-78	0.380118052	1	0.994	4.46E-74	Cd4 Naive	Rpl27a
7.57E-78	1.176887468	0.64	0.274	1.21E-73	Cd4 Naive	Pde4b
3.43E-74	0.381945634	1	0.994	5.50E-70	Cd4 Naive	Rpl17
1.77E-73	0.386682143	1	0.995	2.84E-69	Cd4 Naive	Rplp0
3.29E-73	0.426256731	1	0.992	5.27E-69	Cd4 Naive	Rpl37a
1.68E-72	0.351798538	1	0.994	2.70E-68	Cd4 Naive	Rps13
2.03E-71	0.447765684	1	0.975	3.26E-67	Cd4 Naive	Rpl10a
1.91E-68	0.573518469	0.988	0.921	3.06E-64	Cd4 Naive	Rpl36a
1.30E-66	0.344612105	1	0.996	2.09E-62	Cd4 Naive	Rpl18a
1.29E-65	0.465746679	0.997	0.959	2.07E-61	Cd4 Naive	Rpl10
3.73E-64	0.403187481	1	0.981	5.97E-60	Cd4 Naive	Rpl3
8.77E-64	0.97004808	0.68	0.336	1.41E-59	Cd4 Naive	Ppp1r15a
1.35E-63	0.395055162	0.997	0.983	2.17E-59	Cd4 Naive	Rplp2
5.96E-63	0.831465811	0.522	0.186	9.55E-59	Cd4 Naive	S1pr1
7.52E-63	0.705821896	0.348	0.09	1.21E-58	Cd4 Naive	Sh3bp5
1.20E-62	0.580362676	0.975	0.942	1.93E-58	Cd4 Naive	mt-Cytb

3.58E-62	0.418948417	1	0.978	5.74E-58	Cd4 Naive	Rpl15
7.77E-62	0.867052042	0.745	0.458	1.25E-57	Cd4 Naive	Rapgef6
2.12E-61	0.392013447	1	0.988	3.40E-57	Cd4 Naive	Rps26
3.30E-61	0.787969661	0.28	0.06	5.29E-57	Cd4 Naive	Dapl1
4.94E-61	0.292780583	1	0.998	7.91E-57	Cd4 Naive	Eef1a1
2.11E-59	0.441015175	0.994	0.97	3.38E-55	Cd4 Naive	Rps18
3.50E-59	0.394023699	0.994	0.98	5.61E-55	Cd4 Naive	Rpl26
6.24E-59	0.365059958	1	0.989	1.00E-54	Cd4 Naive	Rpl6
5.00E-58	0.604280983	0.963	0.899	8.01E-54	Cd4 Naive	mt-Nd4l
1.82E-57	0.456776355	0.994	0.979	2.91E-53	Cd4 Naive	mt-Co3
2.61E-57	0.718450026	0.969	0.892	4.19E-53	Cd4 Naive	Junb
1.20E-56	0.445593399	0.994	0.955	1.93E-52	Cd4 Naive	Rpl35
3.98E-55	0.668435155	0.907	0.783	6.38E-51	Cd4 Naive	mt-Nd2
9.16E-55	0.817062268	0.556	0.249	1.47E-50	Cd4 Naive	Foxp1
9.82E-54	0.74584906	0.401	0.131	1.57E-49	Cd4 Naive	Rasgrp2
2.54E-52	0.351063358	1	0.988	4.08E-48	Cd4 Naive	Rpl39
3.23E-52	0.434449647	0.997	0.972	5.17E-48	Cd4 Naive	Rpl14
3.71E-52	0.670560769	0.32	0.088	5.95E-48	Cd4 Naive	Pik3ip1
7.92E-52	0.59647672	0.963	0.849	1.27E-47	Cd4 Naive	Btg1
1.58E-49	0.335928753	1	0.988	2.53E-45	Cd4 Naive	Rpl36
4.18E-48	0.287154943	1	0.995	6.70E-44	Cd4 Naive	Rpl18
1.81E-46	0.292538914	1	0.997	2.90E-42	Cd4 Naive	Rpsa
1.87E-46	0.290041149	1	0.993	2.99E-42	Cd4 Naive	Rpl11
6.70E-44	0.39583842	0.972	0.955	1.07E-39	Cd4 Naive	Rps6
2.18E-43	0.702411221	0.823	0.574	3.50E-39	Cd4 Naive	Zfp36
8.07E-43	0.436911854	0.969	0.939	1.29E-38	Cd4 Naive	Eef1b2
1.31E-42	0.256284193	1	0.995	2.09E-38	Cd4 Naive	Rps15a
3.03E-41	0.758961648	0.475	0.206	4.86E-37	Cd4 Naive	Selenop
1.59E-40	0.706011109	0.668	0.435	2.55E-36	Cd4 Naive	Crlf3
1.77E-40	0.759068045	0.64	0.404	2.84E-36	Cd4 Naive	Dgka
1.40E-39	0.847421152	0.534	0.264	2.24E-35	Cd4 Naive	Jun
2.60E-38	0.31445599	1	0.982	4.17E-34	Cd4 Naive	Rpl28
6.62E-38	0.649249591	0.665	0.37	1.06E-33	Cd4 Naive	Il7r
8.85E-38	0.468073546	0.944	0.872	1.42E-33	Cd4 Naive	mt-Nd1
1.96E-37	0.387197661	0.981	0.933	3.15E-33	Cd4 Naive	Rpl4
2.30E-37	0.375884827	0.978	0.948	3.69E-33	Cd4 Naive	Rack1
3.41E-37	0.312027203	1	0.99	5.47E-33	Cd4 Naive	Rps2
2.13E-36	0.268477688	1	0.994	3.42E-32	Cd4 Naive	Rpl32
1.94E-35	0.317982938	0.991	0.988	3.11E-31	Cd4 Naive	mt-Co2

2.99E-35	0.664499135	0.401	0.168	4.79E-31	Cd4 Naive	Ripor2
1.69E-34	0.67434161	0.413	0.184	2.71E-30	Cd4 Naive	Txk
1.95E-34	0.695564669	0.637	0.4	3.13E-30	Cd4 Naive	Tsc22d3
7.18E-34	0.758102306	0.581	0.33	1.15E-29	Cd4 Naive	Bcl2
7.30E-33	0.812657449	0.714	0.541	1.17E-28	Cd4 Naive	Irf1
1.26E-31	0.597713367	0.72	0.561	2.03E-27	Cd4 Naive	Atp1b3
2.29E-31	0.510685645	0.835	0.712	3.67E-27	Cd4 Naive	Cytip
1.36E-30	0.581856961	0.335	0.136	2.18E-26	Cd4 Naive	Stat5b
2.68E-29	0.42249311	0.276	0.096	4.30E-25	Cd4 Naive	Trat1
1.27E-28	0.433009547	0.944	0.915	2.04E-24	Cd4 Naive	Ddx5
2.52E-28	0.270635879	0.991	0.975	4.03E-24	Cd4 Naive	Rpl22
9.45E-28	0.660405154	0.609	0.427	1.51E-23	Cd4 Naive	Ablim1
3.68E-27	0.502366282	0.817	0.707	5.91E-23	Cd4 Naive	mt-Atp8
1.91E-24	0.37002284	0.913	0.847	3.06E-20	Cd4 Naive	Rps25
6.63E-24	0.526938355	0.329	0.147	1.06E-19	Cd4 Naive	Gm2682
2.15E-23	0.328762534	0.932	0.881	3.45E-19	Cd4 Naive	Npm1
3.60E-23	0.713376893	0.255	0.101	5.78E-19	Cd4 Naive	Gadd45g
3.87E-23	0.349649174	0.953	0.917	6.20E-19	Cd4 Naive	Limd2
2.37E-22	0.313854348	0.935	0.888	3.80E-18	Cd4 Naive	Rps15
3.15E-22	0.437118653	0.792	0.703	5.05E-18	Cd4 Naive	Ets1
6.66E-22	0.590034002	0.714	0.589	1.07E-17	Cd4 Naive	Ier2
1.12E-21	0.500721113	0.649	0.507	1.80E-17	Cd4 Naive	Gm8369
3.10E-20	0.500817335	0.255	0.109	4.98E-16	Cd4 Naive	Zfp281
3.56E-20	0.427241455	0.767	0.716	5.70E-16	Cd4 Naive	Cox7a2l
9.96E-20	0.479990208	0.565	0.382	1.60E-15	Cd4 Naive	Rgs10
8.32E-19	0.49786913	0.373	0.204	1.33E-14	Cd4 Naive	Ssh2
1.80E-18	0.428438162	0.745	0.665	2.89E-14	Cd4 Naive	mt-Nd5
6.38E-17	0.528704348	0.276	0.138	1.02E-12	Cd4 Naive	Stt3b
1.74E-15	0.387291181	0.674	0.585	2.79E-11	Cd4 Naive	Rabac1
2.00E-15	0.427484176	0.304	0.159	3.20E-11	Cd4 Naive	Rasa3
2.38E-15	0.474980132	0.59	0.464	3.82E-11	Cd4 Naive	Socs1
3.06E-15	0.281071894	0.898	0.897	4.90E-11	Cd4 Naive	Ftl1
6.05E-15	0.3611864	0.755	0.707	9.69E-11	Cd4 Naive	mt-Nd4
6.79E-15	0.455803515	0.304	0.165	1.09E-10	Cd4 Naive	Gpr18
1.89E-14	0.310096305	0.829	0.755	3.04E-10	Cd4 Naive	Gimap6
2.79E-14	0.319163492	0.866	0.841	4.47E-10	Cd4 Naive	Rpl22l1
3.18E-14	0.428864146	0.422	0.279	5.10E-10	Cd4 Naive	Mcl1
5.23E-14	0.412213396	0.481	0.359	8.39E-10	Cd4 Naive	Gm10073
1.24E-13	0.399458506	0.289	0.157	1.99E-09	Cd4 Naive	Il4ra

1.39E-13	0.400791554	0.317	0.183	2.22E-09	Cd4 Naive	Pdlim1
3.41E-13	0.405765269	0.46	0.34	5.46E-09	Cd4 Naive	Arhgap15
8.23E-13	0.409701247	0.363	0.23	1.32E-08	Cd4 Naive	Hmgn1
1.03E-12	0.401384445	0.45	0.321	1.65E-08	Cd4 Naive	Eif4a2
1.35E-12	0.451400608	0.286	0.163	2.16E-08	Cd4 Naive	Klhdc2
1.76E-12	0.427778915	0.55	0.421	2.83E-08	Cd4 Naive	Vps37b
3.15E-12	0.374761601	0.637	0.546	5.04E-08	Cd4 Naive	Peli1
4.17E-12	0.389519897	0.571	0.483	6.68E-08	Cd4 Naive	Srsf5
1.06E-11	0.303982169	0.848	0.83	1.69E-07	Cd4 Naive	Pnrc1
1.07E-11	0.276377157	0.845	0.799	1.72E-07	Cd4 Naive	Rpl31
1.29E-11	0.434695133	0.45	0.349	2.07E-07	Cd4 Naive	Gm9493
1.77E-11	0.525846139	0.596	0.508	2.83E-07	Cd4 Naive	Cd69
2.28E-11	0.462871601	0.46	0.358	3.66E-07	Cd4 Naive	Chd3
7.14E-11	0.355751525	0.345	0.226	1.14E-06	Cd4 Naive	Utrn
7.89E-11	0.399494538	0.413	0.299	1.26E-06	Cd4 Naive	Fam102a
1.86E-10	0.322192784	0.652	0.552	2.99E-06	Cd4 Naive	Pim1
2.88E-10	0.425978048	0.612	0.539	4.62E-06	Cd4 Naive	Txnip
4.61E-10	0.265827979	0.609	0.478	7.39E-06	Cd4 Naive	Emb
1.01E-09	0.466814014	0.301	0.202	1.62E-05	Cd4 Naive	Ndrg3
1.03E-09	0.31407816	0.755	0.714	1.65E-05	Cd4 Naive	Arhgap45
1.66E-09	0.34710125	0.388	0.275	2.67E-05	Cd4 Naive	Arl4c
1.84E-09	0.375667236	0.345	0.238	2.95E-05	Cd4 Naive	Stk38
1.94E-09	0.351252025	0.283	0.175	3.10E-05	Cd4 Naive	Slamf6
2.08E-09	0.384704317	0.453	0.357	3.33E-05	Cd4 Naive	mt-Nd3
2.22E-09	0.40664201	0.419	0.327	3.56E-05	Cd4 Naive	Rps27rt
3.30E-09	0.330400036	0.261	0.159	5.30E-05	Cd4 Naive	Tmem71
3.63E-09	0.366531794	0.28	0.173	5.82E-05	Cd4 Naive	Tcp11l2
9.31E-09	0.360493785	0.398	0.301	0.000149255	Cd4 Naive	Tgfbr2
1.33E-08	0.294073422	0.63	0.602	0.000213692	Cd4 Naive	Ube2d2a
1.68E-08	0.274296885	0.997	0.997	0.000269113	Cd4 Naive	Gm42418
1.69E-08	0.291785301	0.677	0.661	0.000270403	Cd4 Naive	Arhgef1
2.05E-08	0.321005769	0.447	0.36	0.000329228	Cd4 Naive	Inpp4b
3.76E-08	0.3312006	0.348	0.25	0.000602801	Cd4 Naive	Clk1
1.04E-07	0.369553524	0.724	0.746	0.001660832	Cd4 Naive	Stk17b
1.17E-07	0.32655385	0.36	0.258	0.001882553	Cd4 Naive	Tspan32
1.30E-07	0.344383278	0.332	0.242	0.002081348	Cd4 Naive	Klf13
1.42E-07	0.388466727	0.391	0.316	0.002281764	Cd4 Naive	Phf20l1
1.83E-07	0.345992412	0.36	0.277	0.002938634	Cd4 Naive	Grap2
2.41E-07	0.391795545	0.357	0.271	0.003858102	Cd4 Naive	Slc38a2

3.72E-07	0.329717246	0.32	0.229	0.005955973	Cd4 Naive	Smad7
6.83E-07	0.251995614	0.54	0.438	0.010941602	Cd4 Naive	Fos
8.39E-07	0.32951239	0.267	0.183	0.013442726	Cd4 Naive	Bzw2
8.43E-07	0.385259749	0.429	0.37	0.013503896	Cd4 Naive	Rnf167
1.14E-06	0.344972699	0.273	0.189	0.018289665	Cd4 Naive	Nfkbiz
1.32E-06	0.491001196	0.28	0.199	0.021113524	Cd4 Naive	Csrnp1
1.75E-06	0.34449488	0.295	0.213	0.028118499	Cd4 Naive	Klhl6
2.14E-06	0.29077851	0.509	0.479	0.034310403	Cd4 Naive	Skp1a
2.44E-06	0.334707823	0.258	0.18	0.039168295	Cd4 Naive	Epc1
2.69E-06	0.294585654	0.429	0.353	0.043164768	Cd4 Naive	Evl
3.84E-06	0.319527366	0.323	0.248	0.061545205	Cd4 Naive	Atp11b
3.91E-06	0.264214407	0.382	0.309	0.062619731	Cd4 Naive	Cdkn1b
5.37E-06	0.330265538	0.419	0.354	0.086002783	Cd4 Naive	Foxo1
5.40E-06	0.309802875	0.32	0.248	0.086556621	Cd4 Naive	Phf3
5.56E-06	0.302153302	0.388	0.321	0.089144671	Cd4 Naive	Fbl
5.81E-06	0.322355559	0.637	0.654	0.093123868	Cd4 Naive	Tpr
6.34E-06	0.344858508	0.276	0.207	0.101555385	Cd4 Naive	Add1
1.02E-05	0.302226467	0.326	0.254	0.164035958	Cd4 Naive	Retreg1
1.57E-05	0.278354643	0.481	0.457	0.252154899	Cd4 Naive	Scp2
1.73E-05	0.281337503	0.252	0.178	0.277563881	Cd4 Naive	Kbtbd11
1.76E-05	0.339215977	0.295	0.229	0.281529874	Cd4 Naive	Add3
1.87E-05	0.285769718	0.528	0.506	0.299251361	Cd4 Naive	Zc3hav1
3.22E-05	0.319230282	0.416	0.372	0.515323608	Cd4 Naive	Psip1
4.95E-05	0.276377476	0.531	0.532	0.793396168	Cd4 Naive	Gm11808
5.80E-05	0.255635184	0.301	0.235	0.929625965	Cd4 Naive	Macf1
7.18E-05	0.288237244	0.752	0.716	1	Cd4 Naive	Nfkbia
9.08E-05	0.299799744	0.376	0.332	1	Cd4 Naive	Mrpl23
9.27E-05	0.278158042	0.373	0.318	1	Cd4 Naive	Gramd1a
0.000121336	0.300836182	0.264	0.205	1	Cd4 Naive	Osbpl9
0.000151126	0.288262931	0.267	0.207	1	Cd4 Naive	Foxn3
0.000193404	0.297126213	0.314	0.259	1	Cd4 Naive	Pik3cd
0.000227335	0.273840524	0.366	0.32	1	Cd4 Naive	2810474O19Rik
0.000272683	0.286511438	0.273	0.216	1	Cd4 Naive	Sun2
0.000345519	0.323362255	0.388	0.361	1	Cd4 Naive	Srpk1
0.000350487	0.296187819	0.301	0.251	1	Cd4 Naive	Ikzf1
0.000455685	0.320401359	0.307	0.258	1	Cd4 Naive	Pdcd4
0.000475653	0.255701032	0.45	0.421	1	Cd4 Naive	Nsd3
0.000693608	0.402883478	0.466	0.453	1	Cd4 Naive	Smc4
0.001388395	0.258779551	0.77	0.777	1	Cd4 Naive	Lars2

0.002028054	0.268123662	0.373	0.346	1	Cd4 Naive	Smchd1
0.002164335	0.290821311	0.252	0.209	1	Cd4 Naive	Kif21b
0.003268469	0.293337651	0.342	0.31	1	Cd4 Naive	Cdkn2d
0.005124053	0.255285429	0.304	0.276	1	Cd4 Naive	Ccm2
0.00918672	0.376799835	0.208	0.306	1	Cd4 Naive	Gbp2
0.009515878	0.274425084	0.304	0.28	1	Cd4 Naive	Rasgrp1

Supplemental Table 3: Genes in CD8 Ki67 Cells

p_val	avg_logFC	pct.1	pct.2	p_val_adj	cluster	gene
0	2.226404826	0.984	0.168	0	Cd8 Hist+Mki67+	Stmn1
0	2.042276388	0.85	0.118	0	Cd8 Hist+Mki67+	Ube2c
0	2.018374999	0.898	0.087	0	Cd8 Hist+Mki67+	Pclaf
0	1.919528595	0.956	0.066	0	Cd8 Hist+Mki67+	Birc5
0	1.832065923	1	0.693	0	Cd8 Hist+Mki67+	Hmgb2
0	1.757138676	0.839	0.232	0	Cd8 Hist+Mki67+	H2afx
0	1.692105305	0.987	0.679	0	Cd8 Hist+Mki67+	Tubb5
0	1.645811772	0.881	0.036	0	Cd8 Hist+Mki67+	Ccna2
0	1.607604129	0.932	0.359	0	Cd8 Hist+Mki67+	Tubb4b
0	1.54066455	0.833	0.063	0	Cd8 Hist+Mki67+	Ccnb2
0	1.533243178	0.773	0.107	0	Cd8 Hist+Mki67+	Top2a
0	1.500366711	0.658	0.049	0	Cd8 Hist+Mki67+	Rrm2
0	1.493902156	0.896	0.081	0	Cd8 Hist+Mki67+	Cks1b
0	1.485140699	0.857	0.243	0	Cd8 Hist+Mki67+	Cenpa
0	1.44394917	0.823	0.193	0	Cd8 Hist+Mki67+	Tuba1b
0	1.443169259	0.981	0.434	0	Cd8 Hist+Mki67+	Hmgn2
0	1.405580727	0.854	0.051	0	Cd8 Hist+Mki67+	Cdca8
0	1.280727426	0.646	0.024	0	Cd8 Hist+Mki67+	Cenpf
0	1.26413451	0.642	0.072	0	Cd8 Hist+Mki67+	Hist1h1b
0	1.257674313	0.644	0.029	0	Cd8 Hist+Mki67+	Cdc20
0	1.254685618	0.801	0.105	0	Cd8 Hist+Mki67+	Smc2
0	1.244398527	0.602	0.061	0	Cd8 Hist+Mki67+	Hist1h2ae
0	1.236947547	0.746	0.042	0	Cd8 Hist+Mki67+	Spc24
0	1.233131396	0.786	0.079	0	Cd8 Hist+Mki67+	Mki67
0	1.230052837	0.761	0.045	0	Cd8 Hist+Mki67+	Cdca3
0	1.217946925	0.688	0.036	0	Cd8 Hist+Mki67+	Cdk1
0	1.211229351	0.971	0.58	0	Cd8 Hist+Mki67+	H2afv
0	1.187149944	0.817	0.157	0	Cd8 Hist+Mki67+	Cks2
0	1.181433786	0.732	0.033	0	Cd8 Hist+Mki67+	Tpx2
0	1.149201703	1	0.81	0	Cd8 Hist+Mki67+	H2afz
0	1.147805325	0.614	0.021	0	Cd8 Hist+Mki67+	Nusap1
0	1.122361044	0.948	0.35	0	Cd8 Hist+Mki67+	Lmnb1
0	1.09936889	0.722	0.043	0	Cd8 Hist+Mki67+	Tacc3
0	1.07911283	0.674	0.056	0	Cd8 Hist+Mki67+	Asf1b
0	1.057777719	0.834	0.22	0	Cd8 Hist+Mki67+	Ube2s
0	1.057528306	0.775	0.118	0	Cd8 Hist+Mki67+	Racgap1

0	1.016265376	0.545	0.019	0	Cd8 Hist+Mki67+	Fbxo5
0	1.004696957	0.938	0.414	0	Cd8 Hist+Mki67+	Selenoh
0	1.001703088	0.989	0.691	0	Cd8 Hist+Mki67+	Ran
0	0.98190967	1	0.972	0	Cd8 Hist+Mki67+	Ptma
0	0.978433305	0.995	0.795	0	Cd8 Hist+Mki67+	Hmgb1
0	0.975042256	0.57	0.02	0	Cd8 Hist+Mki67+	Plk1
0	0.974320717	0.504	0.017	0	Cd8 Hist+Mki67+	Hist1h2ap
0	0.973508561	0.608	0.059	0	Cd8 Hist+Mki67+	Tk1
0	0.967476083	0.6	0.033	0	Cd8 Hist+Mki67+	Aurkb
0	0.962010389	0.609	0.101	0	Cd8 Hist+Mki67+	Tyms
0	0.937943712	0.564	0.023	0	Cd8 Hist+Mki67+	Cenpe
0	0.92713987	0.797	0.231	0	Cd8 Hist+Mki67+	Tmpo
0	0.898812811	0.562	0.024	0	Cd8 Hist+Mki67+	Kif11
0	0.885976381	0.587	0.028	0	Cd8 Hist+Mki67+	Kif22
0	0.879122203	0.525	0.014	0	Cd8 Hist+Mki67+	Hmmr
0	0.873504728	0.659	0.11	0	Cd8 Hist+Mki67+	Incenp
0	0.851332981	0.494	0.027	0	Cd8 Hist+Mki67+	Ccnb1
0	0.84859698	0.518	0.031	0	Cd8 Hist+Mki67+	Cdkn3
0	0.833121165	0.529	0.042	0	Cd8 Hist+Mki67+	Prc1
0	0.825755351	0.627	0.091	0	Cd8 Hist+Mki67+	Cenpw
0	0.796295559	0.545	0.058	0	Cd8 Hist+Mki67+	Knstrn
0	0.783292154	0.505	0.015	0	Cd8 Hist+Mki67+	Ckap2l
0	0.774141167	0.519	0.015	0	Cd8 Hist+Mki67+	Knl1
0	0.768084176	0.529	0.091	0	Cd8 Hist+Mki67+	Tuba1c
0	0.766408551	0.578	0.105	0	Cd8 Hist+Mki67+	Reep4
0	0.74322366	0.461	0.055	0	Cd8 Hist+Mki67+	Gm4316
0	0.710340408	0.562	0.086	0	Cd8 Hist+Mki67+	Hmgb3
0	0.705463455	0.443	0.01	0	Cd8 Hist+Mki67+	Pimreg
0	0.704036692	0.41	0.009	0	Cd8 Hist+Mki67+	Esco2
0	0.701071991	0.553	0.097	0	Cd8 Hist+Mki67+	Rrm1
0	0.669431243	1	0.993	0	Cd8 Hist+Mki67+	Ppia
0	0.668304269	0.572	0.103	0	Cd8 Hist+Mki67+	Lockd
0	0.668290238	0.539	0.073	0	Cd8 Hist+Mki67+	Ccdc34
0	0.663686792	0.47	0.041	0	Cd8 Hist+Mki67+	Cenpm
0	0.661184618	0.459	0.032	0	Cd8 Hist+Mki67+	Kif23
0	0.659190512	0.501	0.049	0	Cd8 Hist+Mki67+	Mad2l1
0	0.653121062	0.434	0.016	0	Cd8 Hist+Mki67+	Cep55
0	0.643948282	0.557	0.106	0	Cd8 Hist+Mki67+	Dbf4
0	0.633998157	0.419	0.019	0	Cd8 Hist+Mki67+	Ndc80

0	0.633554799	0.417	0.013	0	Cd8 Hist+Mki67+	Kif20a
0	0.633442218	0.383	0.025	0	Cd8 Hist+Mki67+	Clspn
0	0.625684189	0.436	0.028	0	Cd8 Hist+Mki67+	Dlgap5
0	0.615316004	0.388	0.054	0	Cd8 Hist+Mki67+	Tcf19
0	0.613671166	0.358	0.006	0	Cd8 Hist+Mki67+	Mxd3
0	0.600667825	0.502	0.088	0	Cd8 Hist+Mki67+	Pmf1
0	0.594738418	0.376	0.012	0	Cd8 Hist+Mki67+	Spc25
0	0.585273896	0.415	0.026	0	Cd8 Hist+Mki67+	Ncaph
0	0.581096387	0.367	0.024	0	Cd8 Hist+Mki67+	Rad51
0	0.580816342	0.397	0.017	0	Cd8 Hist+Mki67+	Cdca2
0	0.576968822	0.407	0.037	0	Cd8 Hist+Mki67+	Ncapd2
0	0.576318988	0.488	0.075	0	Cd8 Hist+Mki67+	Gm10282
0	0.572089327	0.381	0.012	0	Cd8 Hist+Mki67+	Bub1b
0	0.568663741	0.371	0.009	0	Cd8 Hist+Mki67+	Shcbp1
0	0.562149407	0.379	0.018	0	Cd8 Hist+Mki67+	Kifc1
0	0.554534105	0.336	0.014	0	Cd8 Hist+Mki67+	Aurka
0	0.549381154	0.394	0.021	0	Cd8 Hist+Mki67+	Ncapg
0	0.549176924	0.319	0.013	0	Cd8 Hist+Mki67+	Aspm
0	0.546228582	0.347	0.014	0	Cd8 Hist+Mki67+	Rad51ap1
0	0.545099107	0.377	0.032	0	Cd8 Hist+Mki67+	Kif20b
0	0.541994544	0.353	0.009	0	Cd8 Hist+Mki67+	Ska1
0	0.534191261	0.328	0.009	0	Cd8 Hist+Mki67+	Pbk
0	0.525210071	0.352	0.01	0	Cd8 Hist+Mki67+	Cdca5
0	0.52085337	0.354	0.014	0	Cd8 Hist+Mki67+	Cit
0	0.500530347	0.336	0.008	0	Cd8 Hist+Mki67+	Nuf2
0	0.496612573	0.341	0.01	0	Cd8 Hist+Mki67+	Sgo2a
0	0.490241104	0.309	0.011	0	Cd8 Hist+Mki67+	Ckap2
0	0.487117864	0.305	0.009	0	Cd8 Hist+Mki67+	Ccnf
0	0.482813011	0.364	0.042	0	Cd8 Hist+Mki67+	Kif15
0	0.479765145	0.306	0.011	0	Cd8 Hist+Mki67+	Depdc1a
0	0.471414785	0.3	0.007	0	Cd8 Hist+Mki67+	Neil3
0	0.467432273	0.306	0.009	0	Cd8 Hist+Mki67+	Sapcd2
0	0.460417234	0.331	0.021	0	Cd8 Hist+Mki67+	Diaph3
0	0.45270596	0.329	0.036	0	Cd8 Hist+Mki67+	Ube2t
0	0.452650123	0.3	0.008	0	Cd8 Hist+Mki67+	Kif2c
0	0.446378669	0.275	0.011	0	Cd8 Hist+Mki67+	Mis18bp1
0	0.434583762	0.334	0.036	0	Cd8 Hist+Mki67+	Ska2
0	0.434217934	0.267	0.008	0	Cd8 Hist+Mki67+	Nek2
0	0.42491107	0.316	0.019	0	Cd8 Hist+Mki67+	Poc1a

0	0.411354452	0.279	0.007	0	Cd8 Hist+Mki67+	Parpbp
0	0.408869493	0.324	0.035	0	Cd8 Hist+Mki67+	Plk4
0	0.402313878	0.271	0.009	0	Cd8 Hist+Mki67+	Kif4
0	0.36114325	0.258	0.009	0	Cd8 Hist+Mki67+	Gtse1
1.34E-305	0.718178702	0.55	0.112	2.15E-301	Cd8 Hist+Mki67+	Nrm
1.45E-304	0.576820507	0.5	0.09	2.32E-300	Cd8 Hist+Mki67+	Rfc5
6.80E-292	0.401738349	0.263	0.026	1.09E-287	Cd8 Hist+Mki67+	Hist1h2bj
6.42E-290	0.693641071	0.568	0.123	1.03E-285	Cd8 Hist+Mki67+	Ezh2
6.94E-288	0.442243046	0.339	0.044	1.11E-283	Cd8 Hist+Mki67+	Gpsm2
5.79E-278	0.69630767	0.441	0.079	9.28E-274	Cd8 Hist+Mki67+	Kpna2
1.94E-273	0.733043367	0.74	0.22	3.10E-269	Cd8 Hist+Mki67+	Lsm2
2.56E-267	0.598552325	0.515	0.107	4.10E-263	Cd8 Hist+Mki67+	Fen1
5.53E-267	0.818069473	0.807	0.282	8.87E-263	Cd8 Hist+Mki67+	Anp32e
8.34E-264	0.499710288	0.345	0.05	1.34E-259	Cd8 Hist+Mki67+	Uhrf1
5.27E-263	0.370764061	0.289	0.035	8.45E-259	Cd8 Hist+Mki67+	Cenpl
1.69E-262	0.412516178	0.323	0.044	2.72E-258	Cd8 Hist+Mki67+	Rcc1
3.69E-262	0.808649196	0.603	0.156	5.92E-258	Cd8 Hist+Mki67+	Gm42031
6.67E-259	0.96711515	0.829	0.323	1.07E-254	Cd8 Hist+Mki67+	Dut
4.70E-258	0.655492672	0.598	0.148	7.54E-254	Cd8 Hist+Mki67+	Nucks1
3.64E-255	0.534963514	0.273	0.032	5.84E-251	Cd8 Hist+Mki67+	Hist1h1d
5.14E-255	0.681003154	0.607	0.153	8.24E-251	Cd8 Hist+Mki67+	Gmnn
9.48E-253	0.580699009	0.528	0.115	1.52E-248	Cd8 Hist+Mki67+	Haus4
4.24E-250	0.628037184	0.522	0.116	6.80E-246	Cd8 Hist+Mki67+	Mcm7
4.13E-247	0.963827635	0.63	0.177	6.62E-243	Cd8 Hist+Mki67+	Hist1h1e
1.21E-245	0.882127925	0.907	0.437	1.93E-241	Cd8 Hist+Mki67+	Smc4
1.38E-244	0.466289384	0.39	0.067	2.22E-240	Cd8 Hist+Mki67+	Rfc4
1.71E-242	0.544331761	0.334	0.05	2.75E-238	Cd8 Hist+Mki67+	Hist1h3e
1.58E-239	0.618914545	0.476	0.101	2.53E-235	Cd8 Hist+Mki67+	Lig1
1.36E-237	0.421661669	0.379	0.064	2.18E-233	Cd8 Hist+Mki67+	Hjurp
2.80E-233	0.589198746	0.489	0.108	4.49E-229	Cd8 Hist+Mki67+	Mcm5
3.77E-231	0.721337491	0.762	0.262	6.05E-227	Cd8 Hist+Mki67+	Rad21
2.01E-230	0.537116069	0.467	0.099	3.22E-226	Cd8 Hist+Mki67+	Ckap5
5.87E-229	0.561468872	0.527	0.125	9.41E-225	Cd8 Hist+Mki67+	Cmc2
2.89E-222	0.779348615	0.975	0.702	4.62E-218	Cd8 Hist+Mki67+	Anp32b
6.83E-222	0.896618589	0.891	0.462	1.09E-217	Cd8 Hist+Mki67+	Dek
1.25E-220	0.663271871	0.686	0.213	2.00E-216	Cd8 Hist+Mki67+	Fkbp2
8.75E-219	0.412336153	0.324	0.052	1.40E-214	Cd8 Hist+Mki67+	Prim1
8.91E-217	0.416407389	0.36	0.063	1.43E-212	Cd8 Hist+Mki67+	Mns1
3.13E-216	0.367521109	0.314	0.049	5.02E-212	Cd8 Hist+Mki67+	Pkmyt1

6.65E-212	0.537694981	0.522	0.128	1.07E-207	Cd8 Hist+Mki67+	Rdm1
9.73E-210	0.738500956	0.924	0.486	1.56E-205	Cd8 Hist+Mki67+	Banf1
6.51E-207	0.528232471	0.566	0.15	1.04E-202	Cd8 Hist+Mki67+	Rangap1
4.68E-206	0.658105905	0.517	0.131	7.50E-202	Cd8 Hist+Mki67+	Cdc25b
7.00E-203	0.553367134	0.647	0.194	1.12E-198	Cd8 Hist+Mki67+	Lsm3
8.67E-195	0.447845719	0.364	0.071	1.39E-190	Cd8 Hist+Mki67+	Hist1h4d
6.35E-192	0.57587122	0.549	0.155	1.02E-187	Cd8 Hist+Mki67+	Tipin
3.67E-191	0.508632333	0.999	0.933	5.88E-187	Cd8 Hist+Mki67+	Rbm3
1.69E-190	0.518009648	0.998	0.937	2.71E-186	Cd8 Hist+Mki67+	Clic1
6.49E-186	0.559112262	0.996	0.896	1.04E-181	Cd8 Hist+Mki67+	Hnrnpa2b1
5.11E-184	0.646354387	0.993	0.821	8.19E-180	Cd8 Hist+Mki67+	Hnrnpa3
3.51E-183	0.402967728	0.379	0.079	5.62E-179	Cd8 Hist+Mki67+	Tubg1
1.18E-182	0.904424392	0.687	0.248	1.90E-178	Cd8 Hist+Mki67+	Gzmk
3.71E-180	0.44953297	0.472	0.119	5.95E-176	Cd8 Hist+Mki67+	Rnaseh2b
2.54E-169	0.488984547	0.408	0.098	4.07E-165	Cd8 Hist+Mki67+	Cdkn2c
7.70E-168	0.663933964	0.875	0.463	1.23E-163	Cd8 Hist+Mki67+	Dbi
1.66E-165	0.468034335	0.482	0.131	2.66E-161	Cd8 Hist+Mki67+	Nasp
5.66E-165	0.595507313	0.76	0.309	9.07E-161	Cd8 Hist+Mki67+	Ddx39
1.58E-163	0.625250716	0.967	0.69	2.54E-159	Cd8 Hist+Mki67+	Txn1
6.60E-163	0.503026564	0.551	0.169	1.06E-158	Cd8 Hist+Mki67+	Ncaph2
1.78E-162	0.365881497	0.373	0.084	2.85E-158	Cd8 Hist+Mki67+	Anapc15
3.86E-158	0.515205043	0.498	0.144	6.19E-154	Cd8 Hist+Mki67+	Mcm3
5.46E-157	0.414585973	0.388	0.093	8.74E-153	Cd8 Hist+Mki67+	Hells
1.01E-156	0.428262249	0.353	0.081	1.63E-152	Cd8 Hist+Mki67+	Orc6
4.45E-156	0.361201732	0.311	0.063	7.14E-152	Cd8 Hist+Mki67+	Ncapd3
5.26E-155	0.577105994	0.755	0.324	8.43E-151	Cd8 Hist+Mki67+	Lsm5
1.82E-154	0.636324343	0.829	0.413	2.91E-150	Cd8 Hist+Mki67+	Snrpd1
3.48E-150	0.566169003	0.688	0.274	5.58E-146	Cd8 Hist+Mki67+	Anapc5
2.04E-149	0.384730571	0.363	0.086	3.27E-145	Cd8 Hist+Mki67+	Tmem97
8.04E-147	0.682686369	0.896	0.539	1.29E-142	Cd8 Hist+Mki67+	Hnrnpab
1.21E-143	0.463154769	0.483	0.146	1.94E-139	Cd8 Hist+Mki67+	Rpa3
2.35E-143	0.384150496	0.362	0.088	3.77E-139	Cd8 Hist+Mki67+	Alyref
6.36E-142	0.583613864	0.928	0.595	1.02E-137	Cd8 Hist+Mki67+	Calm3
6.90E-140	0.470347896	0.525	0.169	1.11E-135	Cd8 Hist+Mki67+	Fzr1
5.61E-139	0.399649774	0.419	0.114	9.00E-135	Cd8 Hist+Mki67+	Mis18a
2.07E-138	0.645354909	0.839	0.456	3.32E-134	Cd8 Hist+Mki67+	Nap1l1
1.85E-135	0.557074692	0.697	0.292	2.97E-131	Cd8 Hist+Mki67+	Dnajc9
6.59E-135	0.368133544	0.309	0.07	1.06E-130	Cd8 Hist+Mki67+	1500009L16Rik
2.05E-134	0.29910714	0.283	0.059	3.29E-130	Cd8 Hist+Mki67+	Nup37

7.98E-134	0.474690965	0.567	0.197	1.28E-129	Cd8 Hist+Mki67+	Nde1
1.12E-130	0.542748348	0.819	0.403	1.80E-126	Cd8 Hist+Mki67+	Srsf7
9.77E-129	0.505548828	0.723	0.312	1.57E-124	Cd8 Hist+Mki67+	Exosc8
9.97E-129	0.479149001	0.975	0.811	1.60E-124	Cd8 Hist+Mki67+	Hint1
1.03E-124	0.521105176	0.686	0.289	1.65E-120	Cd8 Hist+Mki67+	Smc1a
6.80E-124	0.552030408	0.892	0.515	1.09E-119	Cd8 Hist+Mki67+	Srsf3
1.26E-122	0.515382524	0.834	0.437	2.01E-118	Cd8 Hist+Mki67+	Atpif1
6.20E-122	0.374402085	0.4	0.116	9.94E-118	Cd8 Hist+Mki67+	Hmgn5
2.30E-121	0.538269252	0.938	0.662	3.69E-117	Cd8 Hist+Mki67+	Ywhah
7.96E-120	0.547855246	0.882	0.522	1.28E-115	Cd8 Hist+Mki67+	Srsf2
3.34E-117	0.577726555	0.934	0.693	5.35E-113	Cd8 Hist+Mki67+	Jpt1
1.10E-115	0.395178737	0.998	0.928	1.76E-111	Cd8 Hist+Mki67+	Calm1
6.50E-115	0.419111685	0.459	0.153	1.04E-110	Cd8 Hist+Mki67+	Tfdp1
1.31E-114	0.52873961	0.938	0.626	2.11E-110	Cd8 Hist+Mki67+	Hsp90b1
4.49E-113	0.352810387	0.416	0.128	7.19E-109	Cd8 Hist+Mki67+	Psat1
2.15E-112	0.510475246	0.811	0.436	3.45E-108	Cd8 Hist+Mki67+	Anapc11
1.47E-111	0.429785261	0.533	0.196	2.36E-107	Cd8 Hist+Mki67+	Arsb
1.33E-110	0.450556792	0.566	0.222	2.13E-106	Cd8 Hist+Mki67+	Hpf1
5.42E-110	0.444234333	0.657	0.275	8.69E-106	Cd8 Hist+Mki67+	Csrp1
5.76E-110	0.472684772	0.659	0.286	9.24E-106	Cd8 Hist+Mki67+	Phgdh
4.72E-109	0.265007136	0.261	0.06	7.57E-105	Cd8 Hist+Mki67+	Cbx5
1.28E-107	0.513689114	0.953	0.726	2.06E-103	Cd8 Hist+Mki67+	Emp3
3.63E-107	0.402078331	0.556	0.212	5.81E-103	Cd8 Hist+Mki67+	Sdf2l1
4.42E-107	0.752344533	0.972	0.847	7.09E-103	Cd8 Hist+Mki67+	Arl6ip1
3.88E-105	0.451585692	0.45	0.158	6.22E-101	Cd8 Hist+Mki67+	Pcna
4.56E-105	0.449800785	0.569	0.228	7.30E-101	Cd8 Hist+Mki67+	Usp1
5.65E-105	0.47772367	0.869	0.51	9.05E-101	Cd8 Hist+Mki67+	Nudt21
2.17E-103	0.283992027	0.262	0.063	3.47E-99	Cd8 Hist+Mki67+	Pold1
4.12E-103	0.463588114	0.745	0.371	6.60E-99	Cd8 Hist+Mki67+	Gclm
1.45E-102	0.613765495	0.706	0.371	2.33E-98	Cd8 Hist+Mki67+	Slbp
4.34E-102	0.268608588	0.3	0.078	6.96E-98	Cd8 Hist+Mki67+	Nup85
7.86E-102	0.401053447	0.458	0.159	1.26E-97	Cd8 Hist+Mki67+	Trim59
1.72E-101	0.285966063	0.306	0.082	2.75E-97	Cd8 Hist+Mki67+	Ppih
1.94E-101	0.540731782	0.72	0.375	3.11E-97	Cd8 Hist+Mki67+	Bub3
6.19E-99	0.490460095	0.927	0.656	9.92E-95	Cd8 Hist+Mki67+	Prdx1
7.26E-99	0.430092269	0.693	0.321	1.16E-94	Cd8 Hist+Mki67+	Rnaseh2c
2.33E-98	0.529424718	0.831	0.444	3.74E-94	Cd8 Hist+Mki67+	Cd8a
2.59E-98	0.420443352	0.65	0.286	4.15E-94	Cd8 Hist+Mki67+	Cbx1
1.02E-97	0.301367122	0.271	0.069	1.64E-93	Cd8 Hist+Mki67+	Rfc3

1.09E-97	0.372813711	0.447	0.157	1.74E-93	Cd8 Hist+Mki67+	Nelfe
2.78E-97	0.419675036	0.528	0.209	4.45E-93	Cd8 Hist+Mki67+	Cdca4
3.36E-97	0.401584337	0.981	0.82	5.38E-93	Cd8 Hist+Mki67+	Cox5b
4.27E-97	0.333576127	0.341	0.101	6.85E-93	Cd8 Hist+Mki67+	Nsd2
1.26E-96	0.301155362	0.352	0.106	2.02E-92	Cd8 Hist+Mki67+	Lmf2
3.12E-96	0.415072564	0.51	0.196	5.00E-92	Cd8 Hist+Mki67+	Dctpp1
4.68E-96	0.330511548	0.413	0.137	7.51E-92	Cd8 Hist+Mki67+	Pih1d1
3.82E-95	0.250486204	0.29	0.077	6.12E-91	Cd8 Hist+Mki67+	Rbbp8
5.40E-95	0.580716122	0.852	0.596	8.65E-91	Cd8 Hist+Mki67+	Calm2
1.87E-92	0.253284396	0.285	0.076	3.00E-88	Cd8 Hist+Mki67+	Odf2
1.19E-91	0.42967108	0.943	0.742	1.91E-87	Cd8 Hist+Mki67+	Snrpe
1.43E-91	0.385007777	0.659	0.292	2.30E-87	Cd8 Hist+Mki67+	Lbr
1.83E-91	0.31027967	0.354	0.11	2.94E-87	Cd8 Hist+Mki67+	Atad2
1.85E-91	0.397091143	0.262	0.069	2.96E-87	Cd8 Hist+Mki67+	Hist1h1c
1.88E-91	0.471045845	0.965	0.808	3.01E-87	Cd8 Hist+Mki67+	Tagln2
2.87E-90	0.473122077	0.909	0.612	4.60E-86	Cd8 Hist+Mki67+	Cmtm7
1.10E-89	0.392295722	0.626	0.275	1.76E-85	Cd8 Hist+Mki67+	Hdgf
1.41E-89	0.253807907	0.328	0.097	2.26E-85	Cd8 Hist+Mki67+	Oat
2.49E-89	0.451709619	0.478	0.194	3.99E-85	Cd8 Hist+Mki67+	Siva1
2.96E-89	0.305869839	0.296	0.085	4.74E-85	Cd8 Hist+Mki67+	Sap30
3.44E-89	0.326052586	0.466	0.171	5.51E-85	Cd8 Hist+Mki67+	Ilf2
3.90E-89	0.409162946	0.542	0.225	6.25E-85	Cd8 Hist+Mki67+	Rpa2
5.76E-89	0.333345498	0.492	0.187	9.24E-85	Cd8 Hist+Mki67+	Pbdc1
7.95E-89	0.383618987	0.506	0.201	1.27E-84	Cd8 Hist+Mki67+	Ranbp1
4.23E-88	0.415804618	0.485	0.192	6.78E-84	Cd8 Hist+Mki67+	Dnmt1
2.01E-87	0.411297743	0.972	0.792	3.23E-83	Cd8 Hist+Mki67+	Cox5a
2.30E-86	0.351302036	0.492	0.193	3.68E-82	Cd8 Hist+Mki67+	Nxt1
6.60E-85	0.337743637	0.982	0.871	1.06E-80	Cd8 Hist+Mki67+	Elob
1.81E-84	0.275153094	0.323	0.099	2.89E-80	Cd8 Hist+Mki67+	Agfg2
4.61E-84	0.341594905	0.305	0.093	7.39E-80	Cd8 Hist+Mki67+	1700097N02Rik
5.64E-84	0.323885406	0.4	0.141	9.05E-80	Cd8 Hist+Mki67+	Hat1
7.13E-84	0.264470422	0.324	0.1	1.14E-79	Cd8 Hist+Mki67+	Rhno1
1.33E-83	0.406843586	0.778	0.426	2.14E-79	Cd8 Hist+Mki67+	Mapre1
2.12E-83	0.367263219	0.636	0.292	3.40E-79	Cd8 Hist+Mki67+	Med30
7.09E-83	0.505510293	0.612	0.3	1.14E-78	Cd8 Hist+Mki67+	Cdkn2d
1.46E-82	0.526973586	0.752	0.427	2.34E-78	Cd8 Hist+Mki67+	Dap
1.00E-81	0.340995211	0.472	0.185	1.61E-77	Cd8 Hist+Mki67+	Mthfd2
3.09E-81	0.395150415	0.709	0.356	4.96E-77	Cd8 Hist+Mki67+	Dazap1
6.32E-81	0.344625246	0.462	0.181	1.01E-76	Cd8 Hist+Mki67+	Tuba4a

2.29E-80	0.35990094	0.961	0.785	3.67E-76	Cd8 Hist+Mki67+	Atp5j
6.53E-80	0.351009086	0.473	0.19	1.05E-75	Cd8 Hist+Mki67+	Dtymk
1.30E-79	0.391166131	0.945	0.721	2.08E-75	Cd8 Hist+Mki67+	Cox7a2
2.82E-79	0.400270834	0.873	0.559	4.53E-75	Cd8 Hist+Mki67+	Lsm4
4.89E-79	0.442280511	0.701	0.372	7.83E-75	Cd8 Hist+Mki67+	Rbbp7
6.35E-79	0.398908723	0.711	0.373	1.02E-74	Cd8 Hist+Mki67+	Lsm6
4.47E-78	0.388864477	0.767	0.426	7.16E-74	Cd8 Hist+Mki67+	Plp2
5.24E-78	0.355388491	0.609	0.28	8.39E-74	Cd8 Hist+Mki67+	Sae1
5.37E-78	0.374811312	0.97	0.863	8.61E-74	Cd8 Hist+Mki67+	Sumo2
5.44E-78	0.312581976	0.432	0.164	8.71E-74	Cd8 Hist+Mki67+	Cep57
6.53E-78	0.260436807	0.317	0.101	1.05E-73	Cd8 Hist+Mki67+	Mcm2
1.99E-77	0.362751142	0.982	0.837	3.19E-73	Cd8 Hist+Mki67+	Ybx1
3.15E-77	0.44638015	0.657	0.327	5.06E-73	Cd8 Hist+Mki67+	Hp1bp3
5.95E-77	0.398568303	0.833	0.502	9.54E-73	Cd8 Hist+Mki67+	Gltp
2.67E-76	0.396177192	0.658	0.335	4.28E-72	Cd8 Hist+Mki67+	Cenpx
3.80E-76	0.420796239	0.739	0.408	6.08E-72	Cd8 Hist+Mki67+	Ssrp1
3.11E-75	0.456938445	0.853	0.551	4.98E-71	Cd8 Hist+Mki67+	Hsp90aa1
2.17E-73	0.411801159	0.987	0.898	3.47E-69	Cd8 Hist+Mki67+	Crip1
2.41E-73	0.276244021	0.347	0.119	3.86E-69	Cd8 Hist+Mki67+	Miip
3.21E-73	0.414140389	0.772	0.452	5.14E-69	Cd8 Hist+Mki67+	Mrpl18
4.75E-73	0.445548997	0.842	0.492	7.61E-69	Cd8 Hist+Mki67+	Cd8b1
7.25E-73	0.321007312	0.5	0.211	1.16E-68	Cd8 Hist+Mki67+	Ubl4a
3.34E-72	0.317856855	0.462	0.19	5.35E-68	Cd8 Hist+Mki67+	Ppil1
6.51E-72	0.298071412	0.381	0.144	1.04E-67	Cd8 Hist+Mki67+	Nup62
9.67E-72	0.388816742	0.955	0.709	1.55E-67	Cd8 Hist+Mki67+	Anxa2
1.77E-71	0.277596505	0.392	0.145	2.83E-67	Cd8 Hist+Mki67+	A430005L14Rik
2.31E-71	0.349093657	0.603	0.29	3.70E-67	Cd8 Hist+Mki67+	Nubp1
3.13E-70	0.510601971	0.436	0.184	5.01E-66	Cd8 Hist+Mki67+	Trgv2
3.14E-68	0.288098451	0.458	0.189	5.04E-64	Cd8 Hist+Mki67+	Pcbd2
3.16E-68	0.288035377	0.334	0.119	5.07E-64	Cd8 Hist+Mki67+	Slc29a1
3.46E-68	0.394851638	0.698	0.377	5.55E-64	Cd8 Hist+Mki67+	Cdk4
1.75E-67	0.376493329	0.749	0.425	2.80E-63	Cd8 Hist+Mki67+	U2af1
2.76E-67	0.322663231	0.591	0.277	4.42E-63	Cd8 Hist+Mki67+	Impdh2
5.09E-67	0.316669335	0.978	0.853	8.16E-63	Cd8 Hist+Mki67+	Hnrnpf
5.66E-67	0.344591793	0.775	0.439	9.07E-63	Cd8 Hist+Mki67+	Hnrnpdl
5.70E-67	0.281481632	0.348	0.128	9.13E-63	Cd8 Hist+Mki67+	Pold2
7.52E-67	0.380352415	0.882	0.611	1.21E-62	Cd8 Hist+Mki67+	Sec11c
8.87E-67	0.250708133	1	0.992	1.42E-62	Cd8 Hist+Mki67+	Pfn1
1.10E-66	0.276199989	0.993	0.938	1.76E-62	Cd8 Hist+Mki67+	Cox8a

2.22E-66	0.276316979	0.329	0.119	3.57E-62	Cd8 Hist+Mki67+	Magohb
4.15E-66	0.270779023	0.369	0.138	6.65E-62	Cd8 Hist+Mki67+	Nudt4
5.17E-66	0.328192558	0.518	0.236	8.29E-62	Cd8 Hist+Mki67+	Smc3
1.68E-65	0.322328962	0.539	0.248	2.69E-61	Cd8 Hist+Mki67+	Stard3nl
1.81E-65	0.430785859	0.862	0.608	2.90E-61	Cd8 Hist+Mki67+	1810037I17Rik
3.37E-65	0.313571908	0.521	0.234	5.41E-61	Cd8 Hist+Mki67+	Rfc2
8.84E-65	0.315077527	0.528	0.24	1.42E-60	Cd8 Hist+Mki67+	Nutf2
1.30E-64	0.366660449	0.72	0.398	2.08E-60	Cd8 Hist+Mki67+	Acadl
3.12E-64	0.290620959	0.403	0.161	4.99E-60	Cd8 Hist+Mki67+	Ccng2
1.03E-63	0.339279232	0.944	0.716	1.65E-59	Cd8 Hist+Mki67+	Cox6a1
1.07E-63	0.313883001	0.613	0.301	1.71E-59	Cd8 Hist+Mki67+	Magoh
1.13E-63	0.260963993	0.352	0.133	1.81E-59	Cd8 Hist+Mki67+	Larp7
1.61E-63	0.322073924	0.987	0.871	2.57E-59	Cd8 Hist+Mki67+	Atp5g3
1.63E-63	0.360943506	0.903	0.657	2.62E-59	Cd8 Hist+Mki67+	Raly
3.48E-63	0.271687832	0.3	0.106	5.57E-59	Cd8 Hist+Mki67+	Eri1
6.35E-62	0.362427445	0.547	0.263	1.02E-57	Cd8 Hist+Mki67+	Acsl5
2.63E-61	0.262654196	0.404	0.166	4.21E-57	Cd8 Hist+Mki67+	Tceal9
4.57E-61	0.379817036	0.607	0.316	7.33E-57	Cd8 Hist+Mki67+	Mcm6
4.66E-61	0.331099297	0.667	0.353	7.47E-57	Cd8 Hist+Mki67+	Ssna1
9.38E-61	0.304745848	0.522	0.245	1.50E-56	Cd8 Hist+Mki67+	Dpy30
1.87E-60	0.292335567	0.498	0.229	3.00E-56	Cd8 Hist+Mki67+	CAAA01147332.1
8.45E-60	0.295476304	0.585	0.286	1.35E-55	Cd8 Hist+Mki67+	Idh3a
1.71E-59	0.272701109	0.466	0.206	2.75E-55	Cd8 Hist+Mki67+	Hnrnpd
4.18E-59	0.259282457	0.367	0.147	6.70E-55	Cd8 Hist+Mki67+	Creld2
6.07E-59	0.292317745	0.564	0.275	9.73E-55	Cd8 Hist+Mki67+	Prelid3b
7.27E-59	0.314861071	0.637	0.329	1.17E-54	Cd8 Hist+Mki67+	Nudc
4.49E-58	0.321207859	0.996	0.887	7.19E-54	Cd8 Hist+Mki67+	Lgals1
9.18E-58	0.283047366	0.475	0.213	1.47E-53	Cd8 Hist+Mki67+	Nt5c3b
1.37E-57	0.323804364	0.977	0.815	2.20E-53	Cd8 Hist+Mki67+	Sub1
5.23E-57	0.276165411	0.505	0.234	8.39E-53	Cd8 Hist+Mki67+	Maz
7.91E-57	0.270854615	0.502	0.233	1.27E-52	Cd8 Hist+Mki67+	Mrpl51
9.42E-57	0.348600528	0.734	0.433	1.51E-52	Cd8 Hist+Mki67+	Pa2g4
1.77E-56	0.295850724	0.422	0.183	2.84E-52	Cd8 Hist+Mki67+	Mcm4
3.78E-56	0.36666736	0.71	0.411	6.06E-52	Cd8 Hist+Mki67+	Sh2d1a
1.29E-55	0.265821409	0.337	0.133	2.07E-51	Cd8 Hist+Mki67+	Eomes
1.40E-55	0.336676162	0.83	0.548	2.24E-51	Cd8 Hist+Mki67+	Swi5
5.31E-55	0.284470333	0.996	0.981	8.51E-51	Cd8 Hist+Mki67+	Hspa8
5.23E-54	0.306141462	0.947	0.754	8.38E-50	Cd8 Hist+Mki67+	Psmb9
1.21E-53	0.317101778	0.803	0.491	1.94E-49	Cd8 Hist+Mki67+	Ddx39b

4.95E-53	0.284626556	0.593	0.307	7.93E-49	Cd8 Hist+Mki67+	Ptpa
6.71E-53	0.311659832	0.842	0.558	1.08E-48	Cd8 Hist+Mki67+	Psmb6
1.00E-52	0.26124272	0.478	0.223	1.60E-48	Cd8 Hist+Mki67+	Dynll2
1.70E-52	0.305128194	0.96	0.809	2.73E-48	Cd8 Hist+Mki67+	Prelid1
1.73E-52	0.376030734	0.731	0.459	2.77E-48	Cd8 Hist+Mki67+	Rbbp4
2.22E-52	0.335336076	0.766	0.455	3.55E-48	Cd8 Hist+Mki67+	Rps27l
6.49E-52	0.339715219	0.92	0.714	1.04E-47	Cd8 Hist+Mki67+	Ndufa4
1.66E-51	0.298813428	0.737	0.434	2.66E-47	Cd8 Hist+Mki67+	Snrpc
2.93E-51	0.256625456	0.558	0.283	4.70E-47	Cd8 Hist+Mki67+	Snrnp40
3.58E-51	0.29682603	0.908	0.603	5.73E-47	Cd8 Hist+Mki67+	Ctsw
9.24E-51	0.29144777	0.921	0.69	1.48E-46	Cd8 Hist+Mki67+	Gdi2
1.23E-50	0.273035602	0.561	0.288	1.97E-46	Cd8 Hist+Mki67+	Bex3
3.29E-50	0.301887711	0.604	0.326	5.27E-46	Cd8 Hist+Mki67+	Mrpl42
5.25E-50	0.285042706	0.621	0.334	8.42E-46	Cd8 Hist+Mki67+	Actn4
9.03E-50	0.300000521	0.893	0.643	1.45E-45	Cd8 Hist+Mki67+	Snrpb
1.67E-48	0.275492844	0.71	0.396	2.68E-44	Cd8 Hist+Mki67+	Cyc1
6.07E-48	0.284320738	0.948	0.732	9.73E-44	Cd8 Hist+Mki67+	Reep5
1.79E-47	0.281820069	0.93	0.704	2.86E-43	Cd8 Hist+Mki67+	Ndufb11
3.20E-47	0.278859869	0.551	0.289	5.13E-43	Cd8 Hist+Mki67+	Cntrl
5.79E-47	0.277497674	0.965	0.786	9.28E-43	Cd8 Hist+Mki67+	Psmb3
6.69E-47	0.253637372	0.983	0.876	1.07E-42	Cd8 Hist+Mki67+	Rhoa
2.86E-46	0.303245195	0.739	0.449	4.59E-42	Cd8 Hist+Mki67+	Cycs
3.74E-46	0.283794439	0.744	0.446	5.99E-42	Cd8 Hist+Mki67+	Tcp1
6.91E-46	0.267555551	0.511	0.261	1.11E-41	Cd8 Hist+Mki67+	Snrpa
1.44E-45	0.278944775	0.637	0.352	2.31E-41	Cd8 Hist+Mki67+	Casp3
2.06E-45	0.30822536	0.712	0.428	3.30E-41	Cd8 Hist+Mki67+	Fundc2
3.11E-45	0.252638163	0.433	0.21	4.98E-41	Cd8 Hist+Mki67+	Cisd1
4.45E-45	0.250693039	0.626	0.344	7.14E-41	Cd8 Hist+Mki67+	Naa38
6.38E-45	0.279129603	0.843	0.581	1.02E-40	Cd8 Hist+Mki67+	Sf3b5
7.65E-45	0.338634566	0.904	0.68	1.23E-40	Cd8 Hist+Mki67+	Dynll1
7.72E-45	0.291644623	0.603	0.341	1.24E-40	Cd8 Hist+Mki67+	Smc6
1.33E-44	0.278157841	0.75	0.466	2.13E-40	Cd8 Hist+Mki67+	Manf
1.42E-44	0.301358908	0.86	0.589	2.27E-40	Cd8 Hist+Mki67+	Snrpf
2.78E-44	0.280906794	0.92	0.688	4.46E-40	Cd8 Hist+Mki67+	Eif4a1
3.70E-44	0.308604878	0.979	0.815	5.93E-40	Cd8 Hist+Mki67+	Ms4a4b
5.55E-44	0.279173978	0.835	0.563	8.90E-40	Cd8 Hist+Mki67+	Ywhae
5.98E-44	0.277518997	0.947	0.732	9.58E-40	Cd8 Hist+Mki67+	Atp5f1
8.77E-44	0.261283953	0.701	0.413	1.41E-39	Cd8 Hist+Mki67+	Txnl1
1.15E-43	0.28650373	0.712	0.438	1.85E-39	Cd8 Hist+Mki67+	Hprt

1.86E-43	0.259841373	0.576	0.317	2.97E-39	Cd8 Hist+Mki67+	Dnajc2
2.15E-43	0.264445048	0.87	0.601	3.45E-39	Cd8 Hist+Mki67+	Sept7
3.15E-43	0.291756035	0.775	0.483	5.04E-39	Cd8 Hist+Mki67+	Ndufc2
1.84E-42	0.252540765	0.602	0.337	2.95E-38	Cd8 Hist+Mki67+	Smchd1
1.93E-42	0.271828407	0.727	0.44	3.10E-38	Cd8 Hist+Mki67+	Eloc
2.10E-42	0.386279237	0.593	0.349	3.36E-38	Cd8 Hist+Mki67+	Plac8
4.31E-42	0.291332363	0.834	0.571	6.90E-38	Cd8 Hist+Mki67+	Atp5o.1
2.37E-40	0.270020389	0.788	0.523	3.80E-36	Cd8 Hist+Mki67+	Snrpg
2.78E-40	0.260290764	0.666	0.393	4.46E-36	Cd8 Hist+Mki67+	Ilk
5.72E-40	0.250443777	0.559	0.306	9.17E-36	Cd8 Hist+Mki67+	Klrk1
7.10E-40	0.258825213	0.812	0.549	1.14E-35	Cd8 Hist+Mki67+	Tpm4
7.47E-40	0.260525218	0.833	0.556	1.20E-35	Cd8 Hist+Mki67+	Ndufb8
1.48E-39	0.251426124	0.824	0.537	2.37E-35	Cd8 Hist+Mki67+	Ewsr1
7.12E-39	0.260108687	0.938	0.787	1.14E-34	Cd8 Hist+Mki67+	Atp5b
1.29E-37	0.250007142	0.725	0.456	2.07E-33	Cd8 Hist+Mki67+	Nono
1.67E-37	0.271503238	0.841	0.602	2.67E-33	Cd8 Hist+Mki67+	Sdhb
6.48E-37	0.250961722	0.88	0.67	1.04E-32	Cd8 Hist+Mki67+	Apobec3
1.09E-35	0.263938929	0.96	0.824	1.75E-31	Cd8 Hist+Mki67+	Mif
4.25E-35	0.254373954	0.864	0.602	6.82E-31	Cd8 Hist+Mki67+	Hnrnpa1
6.37E-35	0.255521318	0.865	0.635	1.02E-30	Cd8 Hist+Mki67+	Hnrnpu
3.43E-29	0.557205479	0.257	0.125	5.50E-25	Cd8 Hist+Mki67+	Trbv19

Supplemental Table 4 : Hashtag Sequences per Treatment

IgG
**Hashtag 1**
TRA:CALGNQGGRALIF;TRA:CAVKGGRALIF;TRB:CTCSAAGAEVFF, 12
TRA:CAASEISGSFNKLTF;TRB:CGAREGAGQDTQYF, 9
TRA:CAVSYSNNRIFF;TRB:CASSQETFQDTQYF, 8
TRA:CAASRAGSALGRLHF;TRA:CATDMGYKLTF;TRB:CASSQEKGAGNERLFF, 6
TRA:CALTMTNSAGNKLTF;TRB:CASRQGWNQNTLYF, 6
**Hashtag 2**
TRA:CAVSRGSALGRLHF;TRB:CSSSQRGRGERLFF, 51
TRA:CAAKGNTGNYKYVF;TRB:CASSQEWGGEQYF,17
TRA:CAVNTNTGKLTF;TRB:CASSPDRGQDTQYF,15
TRA:CAAGLGAKLTF;TRA:CALGYAQGLTF;TRB:CASGDARQDTQYF,14
TRA:CAAYTGYQNFYF;TRB:CASGGWGVGEQYF,13
**Hashtag 3**
TRA:CSAKNAGAKLTF;TRB:CASGDGTISNERLFF, 13
TRA:CAASDGTNTGKLTF;TRB:CASSSGLGAETLYF, 8
TRA:CAAEAASSGQKLVF;TRB:CASSQAGHSGNTLYF, 7
TRA:CAASTGNYQLIW, 6
TRA:CALGDYNVLYF;TRB:CASGDSYNSPLYF, 6
**Hashtag 4**
TRA:CAVRTPDYANKMIF;TRB:CASSLNTGQLYF, 81
TRA:CAAKDYSNNRLTL;TRB:CASSVGLGEDTQYF, 71
TRA:CAATDYSNNRLTL;TRB:CASSLELGGLEQYF, 55
TRA:CILRVSGGNNKLTF;TRB:CASSLSGGWYAEQFF, 31
TRA:CAAEDYSNNRLTL;TRB:CASSQVQGSAETLYF, 20
**Hashtag 5**
TRA:CALGEDYSNNRLTL;TRB:CASSQTRDWAYEQYF, 103
TRA:CATAPSSGSWQLIF;TRB:CASGDDWGSQNTLYF, 16
TRA:CATDDYSNNRLTL;TRB:CASSLHTNSDYTF, 16
TRA:CVLSAGSNNRIFF;TRB:CASRQQGNTGQLYF, 16
TRA:CAASMMDSNYQLIW;TRB:CASGDWRKDTQYF, 13

Anti-GITR
**Hashtag 1**
TRA:CAVSVNYNQGKLIF;TRB:CASSRGGGHSGNTLYF, 14
TRA:CAAPNSGTYQRF;TRB:CASGEGQQDTQYF, 5
TRA:CAASATGYQNFYF;TRB:CASSPGTGGYEQYF, 5
TRA:CAAGDSGYNKLTF;TRB:CASSRTVYEQYF, 4
TRA:CAASVGYNQGKLIF;TRB:CASSGGQGIANSDYTF, 4
**Hashtag 2**
TRA:CAAKDYSNNRLTL;TRB:CASSQDKGANTEVFF, 73
TRA:CALSEPNSNNRIFF;TRA:CALDMNYNQGKLIF;TRB:CASSFGTGQLYF, 61
TRA:CAMERDYSNNRLTL;TRB:CASSLELGGTEQFF, 44
TRA:CALSGASSGSWQLIF;TRB:CASSGTISNERLFF, 20
TRA:CALGDRGSGSWQLIF;TRB:CASSITSQNTLYF, 14
**Hashtag 3**
TRA:CAMRDPGTQVVGQLTF;TRA:CALSDDYSNNRLTL;TRB:CASSPPGSSETLYF, 72
TRA:CSASNPDYSNNRLTL;TRB:CASSRTGGSQNTLYF, 33
TRA:CALSPDYSNNRLTL;TRB:CASSQVPDPNSDYTF, 25
TRA:CALSDDYSNNRLTL;TRB:CASSQGQGSQNTLYF, 21
TRA:CAAREDYSNNRLTL;TRB:CASSHPGAETLYF, 20
**Hashtag 4**
TRA:CAAGMSNYNVLYF;TRB:CTCSAENSGNTLYF, 3
TRA:CALGFGGSNAKLTF;TRB:CASSRLGGYEQYF, 3
TRA:CAAQGTGSKLSF;TRB:CASGDARVYEQYF, 2
TRA:CALARYGSSGNKLIF;TRB:CASSPRTAGNTLYF, 2
TRA:CARYNRIFF;TRA:CALGEANTNKVVF;TRB:CGAGLGPSQNTLYF, 2
**Hashtag 5**
TRA:CSAKDYSNNRLTL;TRB:CASSQVQGANERLFF, 292
TRA:CALNGNYGSSGNKLIF;TRA:CALRGTTASLGKLQF;TRB:CASSGGDQNTLYF, 34
TRA:CAAMDYSNNRLTL;TRB:CASSQGQGADTEVFF, 21
TRA:CAANTGANTGKLTF;TRB:CASSDVISYEQYF, 15
TRA:CALRDYNVLYF;TRB:CASSSGTGSQDTQYF, 14
**CTX+IgG**
**Hashtag 1**
TRA:CAAKDYSNNRLTL;TRB:CASSQVGNNERLFF, 85

TRA:CAAGGDTNAYKVIF;TRA:CALGADSGGSNAKLTF;TRB:CAWSGGSAETLYF, 57
TRA:CAAGGDTNAYKVIF;TRB:CAWSGGSAETLYF, 19
TRA:CAAKDYSNNRLTL;TRB:CASSPPGSQNTLYF, 16
TRA:CAVSPPDTNAYKVIF;TRB:CASSHRDWGGAENTLYF, 13
**Hashtag 2**
TRA:CAMESSGSWQLIF;TRB:CASSRQGSNSDYTF, 19
TRA:CAASEGTGNTGKLIF;TRB:CGASTNNERLFF, 16
TRA:CALSSNTNKVVF;TRB:CASGAGTGGQNTLYF, 15
TRA:CALSGSSGSWQLIF;TRB:CASRTANSDYTF, 14
TRA:CAMRDQGGSAKLIF;TRB:CASSDEGRIYEQYF, 14
**Hashtag 3**
TRA:CALGGDTNAYKVIF;TRA:CALVDLSGSGGKLTL;TRB:CASTPLSSNTEVFF, 107
TRA:CALGGDTNAYKVIF;TRB:CASTPLSSNTEVFF, 48
TRA:CAMREASSGSWQLIF;TRB:CASSLDPSSSAETLYF, 15
TRA:CAASGYNQGKLIF;TRA:CAPHGSSGNKLIF;TRB:CASSSTGGSYEQYF, 13
TRA:CAASRANSGTYQRF, 12
**Hashtag 4**
TRA:CALGHDTNAYKVIF;TRB:CASSIRGVTEVFF, 89
TRA:CALGHDTNAYKVIF;TRB:CASSYSGNTLYF, 52
TRB:CASSRTGFTEVFF, 63
TRA:CALGDPWQGGRALIF;TRB:CGARGQASAETLYF, 49
TRA:CALSSDTNAYKVIF;TRB:CASSRTGFTEVFF, 48
**Hashtag 5**
TRA:CAASEEGTQVVGQLTF;TRB:CASSFGLGGRQNTLYF, 16
TRA:CARLAQGLTF;TRB:CASSRLGGASAETLYF, 14
TRA:CANHNAGAKLTF;TRB:CASGDAWGSNERLFF, 12
TRA:CAASGANTGKLTF;TRB:CASSPQGDTGQLYF, 10
TRA:CALTGANTGKLTF;TRB:CGAVDWDQDTQYF, 8
**CTX+Anti-GITR**
**Hashtag 1**
TRA:CADTGNYKYVF;TRA:CALGGDTNAYKVIF;TRB:CASSIQGSNTEVFF, 72
TRA:CAVSPPDYSNNRLTL;TRB:CASSSGGQDTQYF;TRB:CGAREGGGYEQYF, 55
TRA:CALGHDTNAYKVIF;TRA:CALGGDTNAYKVIF;TRB:CASSLLGSQNTLYF, 52
TRA:CALGGDTNAYKVIF;TRB:CASSLLGSQNTLYF, 49

TRA:CAVSPPDYSNNRLTL;TRB:CASSSGGQDTQYF, 18
**Hashtag 2**
TRA:CALGHDTNAYKVIF;TRB:CASTINYAEQFF, 154
TRA:CALGHDTNAYKVIF;TRA:CALGGDTNAYKVIF;TRB:CASTINYAEQFF, 37
TRA:CALSEDYSNNRLTL;TRB:CGATGGGPYEQYF, 34
TRA:CAASPDYSNNRLTL;TRB:CASSLEGKGQQDTQYF, 32
TRA:CALGTQVVGQLTF;TRB:CASSGTISNERLFF, 32
**Hashtag 3**
TRA:CAMEQDYSNNRLTL;TRB:CASSLELGGLEQYF, 180
TRA:CAVSDSNYQLIW;TRB:CASRTGSDYTF, 73
TRA:CALEGDYANKMIF;TRB:CASSENSYEQYF, 63
TRA:CAASWSGGSNYKLTF;TRB:CASSQDRGYEQYF, 55
TRA:CAVSNSGTYQRF;TRA:CALGNYQLIW;TRB:CASSQDGVGQLYF, 42
**Hashtag 4**
TRA:CAASTLNSNNRIFF;TRB:CASTWTGYAEQFF, 101
TRA:CAASANTNKVVF;TRA:CALSGGSNAKLTF;TRB:CASRETGGYEQYF, 42
TRA:CAVSASSGSWQLIF;TRB:CASSQGGQGYNSPLYF, 34
TRA:CAVSISYNQGKLIF;TRB:CASSFSLSGNTLYF, 24
TRA:CAASPPSGTYQRF;TRB:CASSQTGGYTEVFF, 23
**Hashtag 5**
TRA:CAVMDSNYQLIW;TRB:CASSLVGTGNAEQFF, 56
TRA:CAASGDTNAYKVIF;TRB:CASSISGGDEQYF, 24
TRA:CALSRTQVVGQLTF;TRB:CASSAGTNSAETLYF, 16
TRA:CALGHDTNAYKVIF;TRB:CATTWVSGNTLYF, 11
TRA:CAVMDSNYQLIW;TRB:CASSLELGGLEQYF;TRB:CASSLVGTGNAEQFF, 10

Supplemental Table 5: Antibody List

	Flourophore	Clone	Manufacturer	Catalog #
anti-CD8a	PE-Texas red	5H10	Thermofisher	MCD0817
anti-CD4	V450	RM4-5	BD Horizon	560468
anti-Foxp3	Alexa700	FJK-16s	Thermofisher	56-5773-82
anti-Foxp3	APC	FJK-16s	Thermofisher	17-5773-82
anti-Ki67	FITC	B56	BD Bioscience	556026
anti-GITR	APC	DTA-1	Thermofisher	17-5874-81
anti-PD-1	eFlour450	RMP1-30	Thermofisher	48-9981-82
anti-CD25	PercpCy5.5	PC61	BD Bioscience	561112
anti-KLRG1	PE-CF594	2F1	BD Bioscience	565393
anti-Blimp-1	PE	5 E7	Thermofisher	12-9850-82
anti-Eomes	V450	Dan11mag	Thermofisher	48-4875-82
anti-T-bet	PercpCy5.5	O4-46	BD Bioscience	561316
anti-CD4	PE Texas red	RM4-5	BD Bioscience	MCD0417
anti-CD8	BV650	53-6.7	BD Bioscience	563234
anti-Thy1.1	PE-Cy7	HIS51	Thermofisher	25-0900-82
anti-CD44	Pacific blue	IM7	Biolegend	103020
anti-IFNg	PercpCy5.5	XMG1.2	Thermofisher	45-7311-82
anti-TNF	APC	MP6-XT22	BD Bioscience	554420
Ant-CD5	PE	53-7	BD Bioscience	553022
Fc-block		2.4G2	MSKCC Antibody core	
Anti-GITR	In vivo injection	DTA-1	Bioxcell	BE0063
rat IgG2b isotype control	In vivo injection	LTF-2	Bioxcell	BE0090
